# Angiostatic freeze or angiogenic move? Acute cold stress prevents angiokine secretion from murine myotubes but primes primary endothelial cells for greater migratory capacity

**DOI:** 10.3389/fphys.2022.975652

**Published:** 2022-10-17

**Authors:** Pierre Lemieux, Emilie Roudier, Olivier Birot

**Affiliations:** School of Kinesiology and Health Science, Muscle Health Research Center, Faculty of Health, York University, Toronto, ON, Canada

**Keywords:** angioadaptation, angiogenesis, angiokine, VEGF-A, TSP-1, thrombospondin, proteome

## Abstract

The skeletal muscle tissue can adapt to exercise and environmental stressors with a remarkable plasticity. Prolonged cold stress exposure has been associated to increased skeletal muscle capillarization. Angioadaptation refers to the coordinated molecular and cellular processes that influence the remodeling of skeletal muscle microvasculature. Two cell types are central to angioadaptation: the myocytes, representing an important source of angiokines; and the skeletal muscle endothelial cell (SMECs), targets of these angiokines and main constituents of muscle capillaries. The influence of cold stress on skeletal muscle angioadaptation remains largely unknown, particularly with respect to myocyte-specific angiokines secretion or endothelial cell angioadaptive responses. Here, we use an *in vitro* model to investigate the impact of cold stress (28°C *versus* 37°C) on C2C12 myotubes and SMECs. Our main objectives were to evaluate: 1) the direct impact of cold stress on C2C12 cellular expression of angiokines and their release in the extracellular environment; 2) the indirect impact of cold stress on SMECs migration *via* these C2C12-derived angiokines; and 3) the direct effect of cold stress on SMECs angioadaptive responses, including migration, proliferation, and the activation of the vascular endothelial growth factor receptor-2 (VEGFR2). Cold stress reduced the secretion of angiokines in C2C12 myotubes culture media irrespective their pro-angiogenic or angiostatic nature. In SMECs, cold stress abrogated cell proliferation and reduced the activation of VEGFR2 despite a greater expression of this receptor. Finally, SMECs pre-conditioned to cold stress displayed an enhanced migratory response when migration was stimulated in rewarming conditions. Altogether our results suggest that cold stress may be overall angiostatic. However, cold stress accompanied by rewarming may be seen as a pro-angiogenic stressor for SMECs. This observation questions the potential for using pre-cooling in sport-performance or therapeutic exercise prescription to enhance skeletal muscle angioadaptive responses to exercise.

## 1 Introduction

Cold is a fascinating environmental stressor. From acute to chronic exposure, from ambient cold air to ice water immersion, from accidental or occupational adverse effects to clinically- and performance-oriented beneficial applications, cold can challenge for good or bad the homeostasis of our whole body and tissues (Castellani and Tipton, 2015; Tipton, 2019). The skeletal muscle is one of our largest tissues. It participates in important functions such as locomotion, force generation and thermoregulation, as well as endocrine and paracrine functions through the secretion of myokines. The skeletal muscle has a remarkable metabolic, contractile, and microvascular plasticity in response to various physiological, environmental, or pathological conditions (Booth and Thomason, 1991; Hudlicka et al., 1992). Cold stress can alter muscle structure and function, hindering work tolerance and whole-body performance (Doubt, 1991; Oksa, 2002; Cheung et al., 2016). Conversely, cold is proposed as a post-exercise recovery aid to help reduce tissue damage, inflammation, and fatigue (D’Souza et al., 2018; Hohenauer et al., 2018; Ihsan et al., 2016; Joo et al., 2016).

The capillary network is a key determinant of skeletal muscle function (Hudlicka et al., 1992; Poole and Mathieu-Costello, 1996; Hudlicka, 2011; Lemieux and Birot, 2021). The capillary-to-myofiber interface represents the site of exchange for oxygen, nutrients, metabolic waste, myokines, and metabolic heat between the contracting myofibers and the microcirculation (Hudlicka et al., 1992; Poole and Mathieu-Costello, 1996; Hudlicka, 2011; Poole et al., 2020; Lemieux and Birot, 2021). The skeletal muscle capillarization correlates well with mitochondria density, muscle oxidative capacity, muscle oxygen conductance, and endurance exercise capacity (Hoppeler and Kayar, 1988; Poole and Mathieu-Costello, 1996; Howlett et al., 2003; Olfert et al., 2009; Hudlicka, 2011; Tang et al., 2016).

Skeletal muscle angioadaptation refers to the coordinated series of molecular and cellular events that support the plasticity of the skeletal muscle capillarization in the form of either capillary growth (angiogenesis) or regression (Lemieux and Birot, 2021). At the molecular level, angioadaptation is orchestrated by angiokines that can have pro-angiogenic and angiostatic (anti-angiogenic) properties. Pro-angiogenic signals stimulate the proliferation and migration of endothelial cells, the cells forming the capillary wall. Among the plethora of angiokines, the use of transgenic mouse models has identified the pro-angiogenic Vascular Endothelial Growth Factor-A (VEGF-A) and the angiostatic Thrombospondin-1 (THBS-1) as key molecules for regulating skeletal muscle angioadaptation. VEGF-A gene deletion during late gestation resulted in lower capillarization in mouse skeletal muscle and reduced animal exercise capacity (Olfert et al., 2009). Opposite observations were made with THBS-1 gene deletion (Malek and Olfert, 2009). In adult mice, VEGF-A gene inactivation impaired exercise-induced muscle angiogenesis (Breen et al., 2008; Delavar et al., 2014; Tang et al., 2016).

Many studies have investigated the angioadaptive impact of cold stress exposure on skeletal or cardiac muscle capillarization (Heroux and St. Pierre, 1956; Sillau et al., 1980; Wickler, 1981; Banchero et al., 1985; Duchamp et al., 1992; Snyder et al., 1992; Suzuki et al., 1997a; Suzuki et al., 1997b; Mathieu-Costello et al., 1998; Egginton et al., 2001; Deveci and Egginton, 2002; Egginton, 2002; Bae et al., 2003; Deveci and Egginton, 2003; Wagatsuma et al., 2006; Ihsan et al., 2014; Joo et al., 2016; Sugasawa et al., 2016; D’Souza et al., 2018; Al-horani et al., 2019; Opichka et al., 2019; Magalhães et al., 2020; Shute et al., 2020; Krapf et al., 2021). Comparing these studies can be challenging. Research was indeed conducted in human subjects and animals from different species (mice, rats, guinea pigs, pigeons) and different age (growing and juvenile *versus* adult animals). Studies also differed in the muscles or fiber-types analyzed, exposure duration (from a few weeks or months to seasonal variations to multi-generation cold-adapted colonies) and had a focus on cold *per se* or in combination with other stressors such as exercise, ambient hypoxia, and circadian rhythms alterations. Several studies have reported an increase in skeletal muscle capillarization in response to cold stress *per se* (Sillau et al., 1980; Wickler, 1981; Egginton et al., 2001; Deveci and Egginton, 2002; Bae et al., 2003; Deveci and Egginton, 2003).

**FIGURE 1:**
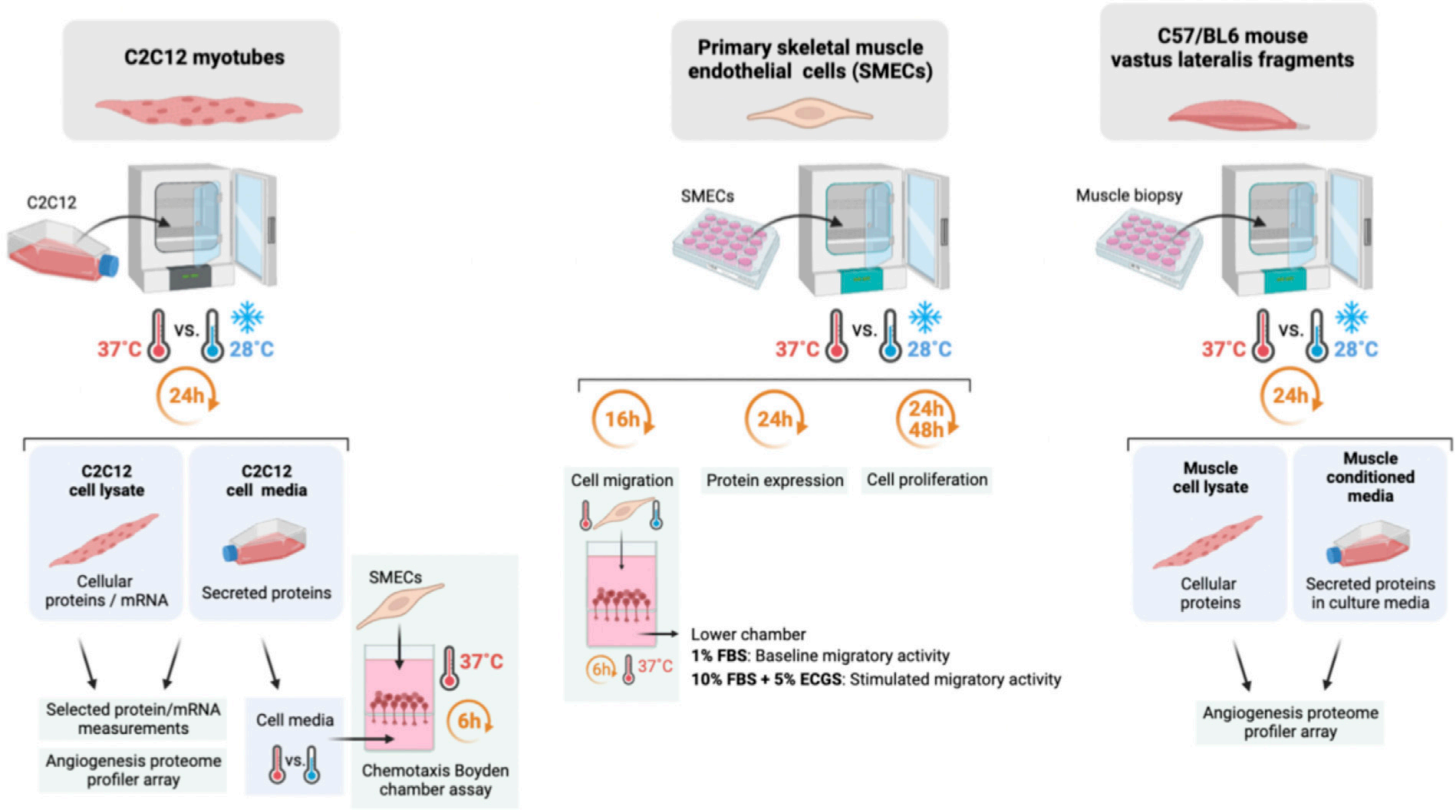
Summary of the methodology approach in mouse myotubes, primary mouse skeletal muscle endothelial cells, and mouse vastus lateralis fragments (EMI assay).

Skeletal muscle angioadaptation requires communication between endothelial cells, as main capillary constituents, and myofibers as an important source of local angiokines (Birot et al., 2003; Olfert and Birot, 2011; Hellsten and Hoier, 2014). While cold has been reported to alter the expression of myokines (Nascimento et al., 2020; Krapf et al., 2021), it remains largely unknown whether cold could regulate angioadaptation through the regulation of myocytes-specific angiokines. To date, a few studies have combined cold and exercise and measured expression levels of the pro-angiogenic VEGF-A. The results remain equivocal probably due to difference in the methodologies (mRNA *versus* protein measurement), exercise protocols (cycling, endurance running, high intensity interval training, resistance training), training durations and intensities, and in the nature of the cold stress (acclimatization to ambient cold air *versus* post-exercise cold water immersion) (D’Souza et al., 2018; Ihsan et al., 2014; Joo et al., 2016; Opichka et al., 2019; Shute et al., 2020; Sugasawa et al., 2016). In addition, these *in vivo* studies have measured VEGF-A expression in whole skeletal muscle biopsies, which does not necessarily reflect myocyte-derived VEGF-A. Yet, myofibers represent the major source of VEGF-A production at rest and in the context of exercise-induced angiogenesis (Birot et al., 2003; Breen et al., 2008; Olfert et al., 2009; Olfert et al., 2010; Delavar et al., 2014). Only two studies have assessed the direct impact of cold stress exposure on VEGF-A expression in isolated myoblasts or myotubes (Sugasawa et al., 2016; Krapf et al., 2021). Here, we propose 1) to evaluate in murine C2C12 myotubes the impact of cold stress exposure on the cellular expression of angiokines and their secretion in the extracellular environment, and 2) to assess whether this can indirectly affect primary murine skeletal muscle endothelial cells (SMECs); 3) to evaluate the direct impact of cold stress exposure on SMECs (Figure 1).

## 2 Methods

### 2.1 Cold stress temperature

C2C12 myotubes, primary skeletal muscle endothelial cells (SMECs), and mouse vastus lateralis muscle fragments were cultured under normothermic (37°C) or cold (28°C) conditions. These temperatures are in the range of what described *in vivo* with physiological exposure of skeletal muscle tissue to cold stress. Previous studies applying standard protocols of cold-water immersion have reported intra-muscular temperature drops from 37–38°C to 28–30°C (Barcroft and Edholm, 1946; Ihsan et al., 2014).

### 2.2 Cell cultures and cold conditionings

#### 2.2.1 Skeletal muscle cells

Mouse skeletal muscle myoblasts (C2C12) (ATCC, Cat. CRL-1772, Cedarlanes, Burlington, ON, Canada) were originally isolated from healthy control mice (Yaffe and Saxel, 1977). C2C12 myoblasts were cultured at 37°C in low glucose (1 g/L) DMEM containing L-glutamine and sodium pyruvate (Cat. 319-005-CL, Wiesent Bioproducts, St Bruno, QC, Canada) and supplemented with 1X penicillin-streptomycin solution (Cat. 450–201-EL, Wiesent Bioproducts, St Bruno, QC, Canada) and 10% Performance FBS (Cat. 098–150, lot 185717, Wiesent Bioproducts, St Bruno, QC, Canada). Upon reaching about 90–95% cell confluence, myoblast differentiation into myotubes was induced at 37°C within 5 days by replacing 10% FBS with 2% horse serum (Cat. 065-105, lot 52659, Wiesent Bioproducts, St Bruno, QC, Canada).

C2C12 myotubes were exposed to 37°C or 28°C for 6 h or 24 h before cell lysis and cell culture media collection. We ensured that C2C12 differentiation was not altered by these 24 h culture nor by the cold exposure (images available in Supplementary Material). The analysis of the impact of cold exposure was always made by comparing C2C12 myotubes differentiated from the same original myoblast batch and using the same lots of FBS and Horse serum. The impact of alternating cooling, re-warming, and re-cooling on THBS-1 protein expression was assessed in C2C12 by culturing the cells at 37°C for 36 h (normothermic control condition), at 37°C for 24 h followed by 12 h at 28°C (cold exposure), at 37°C for 12 h, 28°C for 12 h, and 37°C for 12 h (re-warming), and at 28°C for 12h, 37°C for 12 h, and again to 28°C for 12 h (re-cooling). The overall conditioning was of 36 h of cell culture in all four groups.

#### 2.2.2 Primary mouse skeletal muscle endothelial cells

Mouse C57BL/6 primary skeletal muscle microvascular endothelial cells (SMECs) (Cell Biologics Inc., Cat. C57-6220, Cedarlanes, Burlington, ON, Canada) were plated on gelatin-coated dishes (1.5% Gelatin Type A (BioShop, Cat. Gel771.500, BioShop Canada Inc., Burlington, ON, Canada) and cultured in complete endothelial cell media (Cell Biologics Inc., Cat. M1168, Cedarlanes, Burlington, ON, Canada). Cells were used between passages P4 and P8 at a density of about 350,000 cells/60-mm dish. The analysis of the impact of cold exposure was always made by comparing SMECs cultured from the same original SMEC batch, same passage, and same cell seeding density.

A first conditioning was performed to assess the impact of secreted proteins from C2C12 (cultured at 37°C or 28°C) on SMECs migratory activity. Additional SMEC conditionings were performed to assess the effect of cold exposure on cell proliferation (n = 6 independent experiments with *n* = 6 petri dishes per condition), cell migration, or protein expression (cold marker RBM3, VEGF receptor-2). The responsiveness of VEGF receptor-2 (VEGFR2) was evaluated by measuring its phosphorylation level on Tyrosine residue 1175 in response to stimulation with murine recombinant VEGF-A_165_ (mVEGF-A165, Peprotech, Cat. 450-32-10UG, lot. 031199 K1621, Cedarlanes, Burlington, ON, Canada). Briefly, SMECs were pre-conditioned at 37°C or 28°C for 23 h (12 h in complete endothelial cell media and 11 h in starved media with 1% FBS only) before being incubated with mVEGF-A165 (100 ng/ml) for 1 h.

### 2.3 Isolation of cellular and secreted proteins from cell conditionings

C2C12 and SMECs cellular protein extracts were collected after cell lysis in a buffer containing 50 mM Tris-base, 100 mM NaCl, 5 mM EDTA, 1% sodium deoxycholate, 1% triton X-100, 1 mM phenylmethylsulfonyl fluoride (PMSF), 1 mM NaF, 1 mM Na3VO4, protease inhibitor cocktail (Roche Complete Mini, Cat. 04906845001, Sigma-Aldrich, Oakville, ON, Canada), phosphatase inhibitor cocktail (Roche PhosSTOP, Cat. 11836153001, Sigma-Aldrich, Oakville, ON, Canada), pH 8. After 20 min lysis incubation at 4°C, cell lysates were centrifugated at 16,000 g and 4°C for 15 min, and supernatants were collected.

Secreted proteins from C2C12 myotubes were collected from cell media. Five hundred µl of cell media were loaded on 100 kDa (for THBS-1 detection) or 3 kDa (for the detection of VEGF-A and proteome array targets) MWCO protein concentrators (Pierce, Cat. 88503 and 88512, ThermoFisher Scientific, Burlington, ON, Canada) and centrifugated at 12,000 g for 10 and 30 min, respectively.

### 2.4 *Ex vivo* muscle incubation assay and protein isolation

Mouse vastus lateralis muscles were collected from six sedentary 11-weeks old C57B/L6 female mice (027C57Bl/6, Charles River Laboratory, Senneville, QC, Canada) accordingly to the guidelines of the Canadian Council on Animal Care and York University Committee on Animal Care (Approval #2020-05). Animals were kept in a 12:12 light:dark cycled, temperature-controlled environment with water and diet *ad libitum* in order to acclimatize to the animal facility. Mice were fasted for 2 h before anesthesia with isoflurane. Vastus lateralis muscles were collected from anaesthetized animals, weighed, and divided into two smaller fragments of about 40–60 mg. These fragments, obtained by cross sectioning the muscle in its mid-belly region, reflect the entire muscle cross-section to minimize regional different in the contractile and metabolic phenotype of the vastus lateralis muscle. Mice were euthanized by heart removal. *Ex vivo* muscle incubation (EMI) assay was adapted from Aiken et al. (2016). Briefly, fragments of vastus lateralis muscles were rinsed three times in cold PBS before *in vitro* incubation in low glucose (1 g/L) DMEM (Cat. 319-005-CL, Wiesent Bioproducts, St Bruno, QC, Canada) with 10% Performance FBS (Cat. 098-150, lot 185,717, Wiesent Bioproducts, St Bruno, QC, Canada), 1X penicillin-streptomycin solution (Cat. 450-201-EL, Wiesent Bioproducts, St Bruno, QC, Canada). Following incubation, muscle fragments were rinsed in cold PBS, homogenized in protein lysis buffer containing 50 mM Tris-base, 100 mM NaCl, 5 mM EDTA, 1% sodium deoxycholate, 1% triton X-100, 1 mM phenylmethylsulfonyl fluoride (PMSF), 1 mM NaF, 1 mM Na3VO4, protease inhibitor cocktail (Roche Complete Mini, Cat. 04906845001, Sigma-Aldrich, Oakville, ON, Canada), phosphatase inhibitor cocktail (Roche PhosSTOP, Cat. 11836153001, Sigma-Aldrich, Oakville, ON, Canada), pH 8 with a MM400 tissue lyser (30 pulse/sec., 4°C, Retsch GmbH, Haan, Germany). Following centrifugation (16,000 g, 4°C, 15 min) protein lysates were concentrated on 3 kDa MWCO protein concentrators (Pierce, Cat. 88512, ThermoFisher Scientific, Burlington, ON, Canada), and stored at −80°C until further analysis. For the detection of proteins secreted by the muscle fragments in the EMI assay, cell media was treated as previously described in paragraph 2.3. for secreted C2C12 proteins.

### 2.5 Immunoblotting of cellular and secreted proteins

Immunoblotting for cellular and secreted proteins was carried out on protein extracts from C2C12 myotubes, SMECs, vastus lateralis fragments (EMI assay), and cell media. Total protein concentration was determined in cell lysates and cell media by BCA assay (Sigma-Aldrich, Cat. B9643, Oakville, ON, Canada) as previously described (Roudier et al., 2012, 2010; Aiken et al., 2016; Lam et al., 2021). An amount of 30 µg of cellular C2C12 proteins, 100 µg of secreted C2C12 proteins, 30 µg of cellular SMECs proteins, or 30 µg of cellular EMI proteins were diluted in a 1/5 vol./vol. ratio in a loading buffer (2.69 mM sucrose, 0.28 M SDS, 2.84 M b-mercaptoethanol, 14 µM bromophenol blue, 0.5 M Tris-base pH 6.8) separated by SDS-PAGE, and then blotted onto nitrocellulose membranes (Whatman Cat. BA95; Sigma-Aldrich, Oakville, ON, Canada). Quality of the transfer was controlled by Ponceau S Red staining. After blocking with 5% fat-free milk in 0.1% tween tris-buffered saline buffer, membranes were probed with the following primary antibodies: Mouse monoclonal anti-thrombospondin-1 (1:200, clone A6.1, Invitrogen, Cat. MA513398, ThermoFisher Scientific, Burlington, ON, Canada); rabbit monoclonal anti-thrombospondin-1 (1:1000, clone D7E5F, Cell Signaling, Cat. 37879, New England Biolabs, Whitby, ON, Canada); rabbit monoclonal anti-Phospho-VEGF Receptor-2 (Tyr1175) (1:1000, clone D5B11, Cell Signaling, Cat. 3770, New England Biolabs, Whitby, ON, Canada); rabbit monoclonal anti-VEGF Receptor-2 (1:1000, clone D5B1, Cell Signaling, Cat. 9698, New England Biolabs, Whitby, ON, Canada); rabbit monoclonal anti-RBM3 (1:1000, clone EPR6061(2), Cat. ab134946, Abcam, Toronto, ON, Canada). β-actin and α/β-tubulin were detected as protein loading controls for the analysis of cellular proteins (mouse monoclonal anti-b-actin, 1:1000, clone C4, Cat. sc-47778, Santa-Cruz Biotechnology, Santa-Cruz, CA, United States; rabbit polyclonal anti-a/b-tubulin, 1:1000, Cell Signaling, Cat. 2148, New England Biolabs, Whitby, ON, Canada). The quality of loading was also controlled by Ponceau S staining in addition to β-actin and α/β-tubulin detection (Supplementary Material). For secreted proteins, the Ponceau S staining was used as a loading control. Membranes were then incubated with the following secondary HRP-conjugated antibodies: Goat anti-rabbit IgG (1:1000, Cell Signaling, Cat. 7074, New England Biolabs, Whitby, ON, Canada), Rabbit anti-mouse IgG (1:1000, Dako, Cat. P0260). Proteins were visualized with enhanced chemiluminescence (Pierce ECL Western Blotting Substrate, Cat. 32106, ThermoFisher Scientific, Burlington, ON, Canada; SuperSignal West Atto Ultimate Sensitivity Chemiluminescent Substrate, Cat. A38554, ThermoFisher Scientific, Burlington, ON, Canada; SuperSignal West Dura Extended Duration Substrate, Cat. 34075, ThermoFisher Scientific, Burlington, ON, Canada). Images were obtained at several exposure durations on an imaging station (Kodak 4000 MM Pro; Carestream Health, Concord, ON, Canada) equipped with a CCD camera. Images were analyzed and protein expression levels quantified using NIH ImageJ imaging software.

### 2.6 VEGF-A protein determination by ELISA

Cellular and secreted VEGF-A protein concentration was quantified respectively in cell lysates and cell media from C2C12 myotubes cultured for 24 h at 37°C or 28°C with a mouse VEGF Quantikine ELISA kit (R&D Systems, Cat. MMV00, Cedarlanes, Burlington, ON, Canada) accordingly to the manufacturer’s instructions and using respectively 20 μg and 75 µg of total cellular and secreted proteins.

### 2.7 Total RNA isolation and real-time qPCR

Total RNAs were isolated from C2C12 myotubes lysate following 6h and 24 h exposure to 37°C or 28°C (n = 3 independent experiments with n = 6 samples per condition) using the QIAzol Lysis Reagent (Qiagen, Cat. 79306, Toronto, ON, Canada) as previously described (Lam et al., 2021). RNA concentration was determined by measuring absorbance at 260 and 280 nm. mRNAs were then reverse-transcribed into cDNAs using the High-Capacity RNA-to-cDNA kit (Applied Biosystems, Cat. 4387406, ThermoFisher Scientific, Burlington, ON, Canada). Semi-quantitative real-time PCR was performed for each sample in triplicate using TaqMan^®^ Gene Expression assay probes and TaqMan^®^ Universal Master Mix II (Applied Biosystems, Cat. 4440042, ThermoFisher Scientific, Burlington, ON, Canada) for 40 cycles of denaturation at 95°C for 3 s, followed by annealing and extension at 60°C for 30 s. Thrombospondin-1 (*THBS-1*) was measured and the ∆∆Ct method was used to express the relative changes. mRNA changes were normalized to *HPRT* gene. Data and expressed as $2^{-∆∆Ct}$ . Reference numbers and probe sequences were Mm01335418_m1, GAA​ATA​CGA​GTG​TCG​AGA​TTC​CTA for THBS1, and Mm01545399_m1, GGA​CTG​ATT​ATG​GAC​AGG​ACT​GAA​A for HPRT.

### 2.8 Mouse angiogenesis proteome profiler array

C2C12 myotubes and vastus lateralis fragments from the EMI assay were exposed for 24 h to either 37°C or 28°C. Cellular and secreted proteins were isolated as previously described (*n* = 6 per conditions: cellular vs. secreted proteins, 37°C vs. 28°C). Samples from each group were pooled and total protein concentrations of each pool were assessed by BCA assay. The assay was run accordingly to the manufacturer’s instructions (R&D Systems, Cat. ARY015, Cedarlanes, Burlington, ON, Canada) and as previously described (Roudier et al., 2012). An amount of 250–300 μg and 400 µg of total protein from C2C12 myotubes and EMI, respectively, were incubated with the array nitrocellulose membranes pre-probed with primary antibodies against 53 pro-angiogenic or angiostatic molecules. Target protein spots were visualized by incubating each membrane with a cocktail of biotinylated secondary antibodies and streptavidin-HRP and using an enhanced chemiluminescence procedure (SuperSignal West Dura Extended Duration Substrate, Cat. 34075, ThermoFisher Scientific, Burlington, ON, Canada). Protein spots were quantified using a Kodak Imaging station 4000 MM Pro equipped with Carestream software and a CCD camera and analyzed using NIH ImageJ Software.

### 2.9 Boyden chamber migration assay

Endothelial cell migratory response was assessed by performing Boyden chamber chemotaxis assays as previously described (Aiken et al., 2016). Following trypsinization, SMECs were loaded in the upper chamber at a density of 500 cells/µl. Cells migrated through an 8-μm polycarbonate membrane filter (NeuroProbe, Inc., Cat. PFB8, Cedarlanes, Burlington, ON, Canada) coated with 50 μg/ml collagen (Gibco, Cat. A10438-01, ThermoFisher Scientific, Burlington, ON, Canada) in 0.02 M acetic acid (Sigma-Aldrich # 320,099). In each Boyden chamber experiment, the basal level of SMECs migration was assessed by loading the bottom chamber with 28 µL of DMEM supplemented with 1% FBS. A positive control for migration was incorporated on each assay by loading the bottom chamber with 28 µL of DMEM supplemented with 10% FBS and 5% endothelial cell growth supplement (ECGS 100x, ScienCell, Cat.1052, Cedarlanes, ON, Canada). SMECs migration was then assessed in response to stimulation by C2C12-secreted proteins collected after 24 h exposure to 37°C or 28°C (indirect C2C12-mediated impact of cold) or by pre-conditioning SMECs for 16 h at 37°C or 28°C (direct impact of cold on SMEC). For indirect and direct evaluation of cold exposure, 6 and 5 independent Boyden chamber experiments were run, respectively (n = 6 migration wells per condition). After 6 h of migration time at 37°C, the nitrocellulose membrane filter was fixed in cold methanol and stained with 10% Giemsa stain diluted in water (Cat. R03055-74, Sigma-Aldrich, Burlington, ON, Canada), and mounted on a glass microscope slide. For each experiment, four individual fields of view per well were counted under ×10 magnification, and results were presented as the average of all fields of view.

### 2.10 Skeletal muscle endothelial cell proliferation

SMECs were seeded on a 96-wells plate at 1,000 cells/well density at day 0 and cultured at 37°C or 28°C (direct cold exposure). Cell proliferation was quantified at days 0, 1, and 2 by using the cyQUANT^®^ NF Cell proliferation Assay kit (Invitrogen, Cat. C35007, ThermoFisher Scientific, Burlington, ON, Canada) accordingly to the manufacturer’s instructions. Briefly, the assessment of cell proliferation is based on quantifying the fluorescence emitted at 485 nm by a cell-permeant DNA-binding dye. Three independent cell conditionings were performed with *n* = 4-8 cell culture wells per condition (proliferation at 37°C *versus* 28°C) and per time-point (days 0, 1, and 2). In a second experiment, cells were preconditioned to cold exposure (28°C) for 16 h before returning at 37°C (day 0). Cell proliferation was quantified at days 0, 1, and 2 as previously described. Three independent cell conditionings were performed with *n* = 6-7 cell culture wells per condition (preconditioning at 37°C *versus* 28°C) and per time-point (days 0, 1, and 2).

### 2.11 Statistical analysis

Statistical analyses were performed using unpaired student’s t-test, 1-way or 2-way ANOVA using Prism9 (GraphPad software), Tukey multiple comparison post-tests for ANOVA analyses. *p* < 0.05 was considered to be statistically significant. Evaluating the impact of alternating cooling, rewarming, and re-cooling on THBS1 protein expression in C2C12 was conducted on 2 independent experiments (*n* = 6 samples per condition). VEGF-A quantification by ELISA in C2C12 was performed on *n* = 6 samples per temperature condition. Other protein detection in C2C12 and SMECs was performed by western blotting in *n* = 3–4 independent experiments (*n* = 3–8 samples per condition). Proliferation assays were conducted on *n* = 3 independent experiments (*n* = 6–8 samples per condition). Boyden chamber assays included *n* = 6 (stimulation with C2C12 secreted proteins) or *n* = 5 (cold pre-conditioning) independent experiments (*n* = 6 samples per condition). Protein measurements in EMI vastus lateralis fragments were performed on *n* = 6 samples per condition. Detailed statistical analyses are available in the Statistical table in Supplementary Material.

## 3 Results

### 3.1 Exposure of C2C12 myotubes to 28°C led to a cold stress response and reduced secreted VEGF-A protein concentration

**FIGURE 2:**
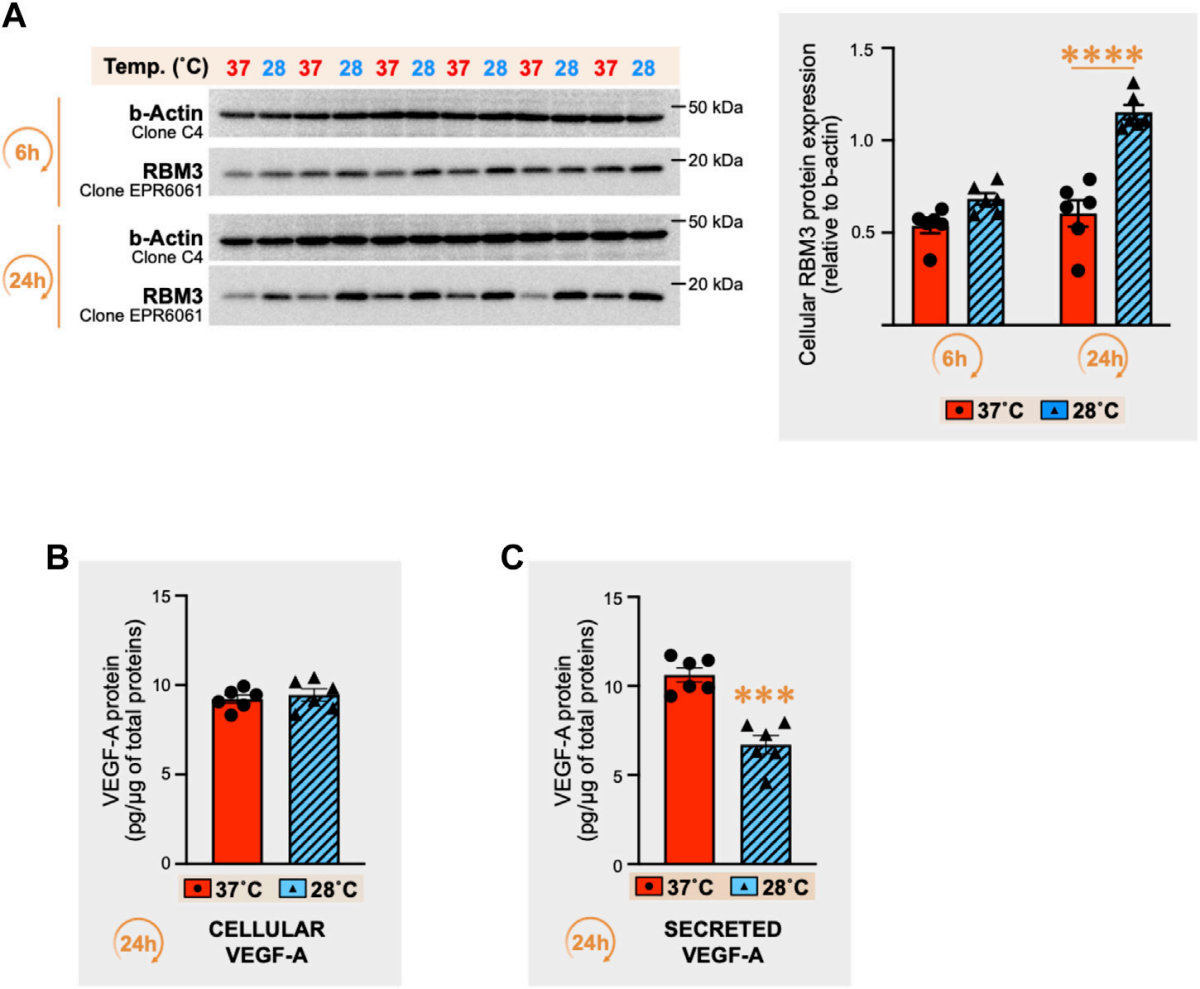
Effect of cold stress exposure on RNA-binding motif 3 protein and vascular endothelial growth factor-A protein expression in C2C12 myotubes. **(A)** Representative immunoblots and densitometry analyses for cellular protein levels of the cold-shock RNA-binding motif 3 protein (RBM3) in mouse C2C12 myotubes exposed for 6h and 24 h to cold stress (28°C) or maintained at 37°C. β-actin is used as a loading control. Significant difference between groups: ****, *p* ≤ 0.0001. Data is represented as means ± SEM from three independent experiments, each with *n* = 6 samples per condition of exposure and temperature. **(B, C)** ELISA quantification of cellular **(B)** and secreted **(C)** protein levels for Vascular Endothelial Growth Factor-A (VEGF-A) in the C2C12 samples exposed for 24h to 37°C or 28°C (pg of VEGF-A per µg of total proteins). Data is represented as means ± SEM from *n* = 6 samples. Significant difference: ***, *p* ≤ 0.001.

The RNA-binding motif 3 (RBM3) protein is a cold stress marker (Wellmann et al., 2004; Dupont-Versteegden et al., 2008; Ferry et al., 2011; Zhu et al., 2016; Cuthbert et al., 2019). RBM3 protein was detected in C2C12 myotubes exposed for 6h and 24 h to cold stress (28°C) or maintained at 37°C (Figure 2A). Whereas no significant change was observed at 6h, a strong increase in RBM3 protein expression was observed after 24 h of cold (respectively +75% (*p* ≤ 0.0001), +90% (*p* ≤ 0.0001), and +20% (*p* = 0.004) in three independent experiments). We therefore quantified cellular and secreted VEGF-A protein levels in C2C12 myotube culture after 24 h of cold stress exposure. Whereas cellular VEGF-A protein levels remained unchanged (Figure 2B), cold exposure led to a significant reduction (-51%) of secreted VEGF-A protein concentrations (Figure 2C, *p* = 0.0001).

### 3.2 Cold stress downregulates the level of a cleaved secreted THBS-1 fragment in the cell media but upregulates cellular THBS-1 protein in C2C12 myotubes

THBS-1 and VEGF-A are respectively defined as key angiostatic and pro-angiogenic molecules in the skeletal muscle (Breen et al., 2008; Malek and Olfert, 2009; Lemieux and Birot, 2021). In the extracellular matrix, active processes cleave THBS-1 to generate bioactive THBS-1 fragments with lower molecular weights (Kessler et al., 2015). Therefore, we analyzed cellular and secreted THBS-1 protein levels from the above set of samples (Figures 2B,C) through immunodetection with the mouse monoclonal anti-THBS-1 clone A6.1, as previously described (Roudier et al., 2010; Roudier et al., 2012; Aiken et al., 2016; Aiken et al., 2019).

**FIGURE 3:**
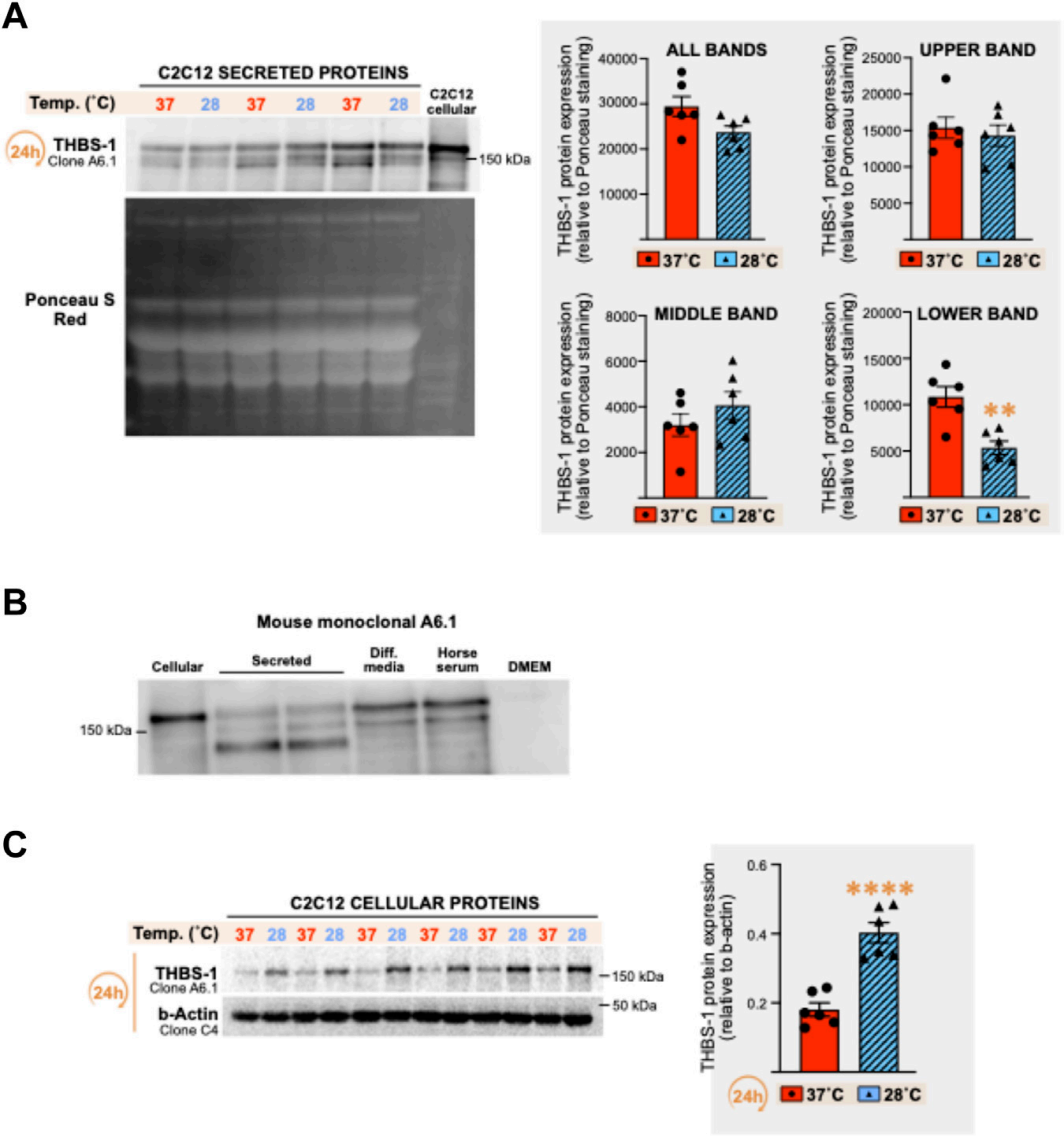
Effect of cold stress exposure on thrombospondin-1 protein expression pattern in C2C12 myotubes. **(A)** Representative immunoblots and densitometry analysis for thrombospondin-1 (THBS-1) protein expression levels in conditioned cell media from C2C12 myotubes cultured at 37°C or 28°C for 24 h. Ponceau S Red staining is used as a loading control. Significant difference: **, *p* ≤ 0.01. Data is represented as means ± SEM from the *n* = 6 samples presented in Figure 2 for VEGF-A protein quantification and is representative of four independent experiments conducted with *n* = 3–6 samples per condition. **(B)** Representative immunoblots for THBS-1 protein detected with the mouse monoclonal anti-THBS-1 clone A6.1 in cell lysate (cellular), cell media (secreted) from C2C12 myotubes, in the differentiation media containing no cell (Diff. media), in the horse serum, and in DMEM media alone (with no cells). **(C)** Representative immunoblots and densitometry analysis THBS-1 protein expression levels in cell lysates (cellular) from the C2C12 myotubes presented in Panel **(A)**. β-actin is used as a loading control. Significant difference: ****, *p* ≤ 0.0001. Data is represented as means ± SEM from the *n* = 6 samples presented in Figure 2 for VEGF-A protein quantification.

The pattern of secreted THBS-1 expression revealed three bands, with either a significant reduction or a trend toward a lower expression of the lower one (Figure 3A: -49%, *p* = 0.0019; -29%, *p* = 0.002; -52%, *p* = 0.059; -56%, *p* = 0.0096) or when combining all observed bands (Figure 3A: -16%, n.s; -18%, *p* = 0.043; -27%, *p* = 0.003; -20%, n.s). Surprisingly, the two top bands were also detected in the differentiation media alone (no cells) and in the horse serum (Figure 3B). No band was detected in DMEM alone (Figure 3B). The two bands detected in the cell differentiation media could result from cross-reactivity of the antibody with horse THBS-1 protein. Yet and since the lower band was previously reported as a secreted, cleaved, and bioactive C-terminal fragment of THBS-1 (Kessler et al., 2015), our data supports the notion that this THBS-1 fragment is specific to the extracellular compartment and was negatively regulated by 24 h of cold stress (Figure 3A).

Conversely to cellular VEGF-A expression, which was not altered by cold exposure (Figure 2B), we observed a strong increase in cellular THBS-1 signal in response to 24 h of cold exposure (Figure 3C, +122%, *p* ≤ 0.0001).

### 3.3 Cold stress exposure stimulates expression of both THBS-1 mRNA and protein in C2C12 cell lysates

**FIGURE 4:**
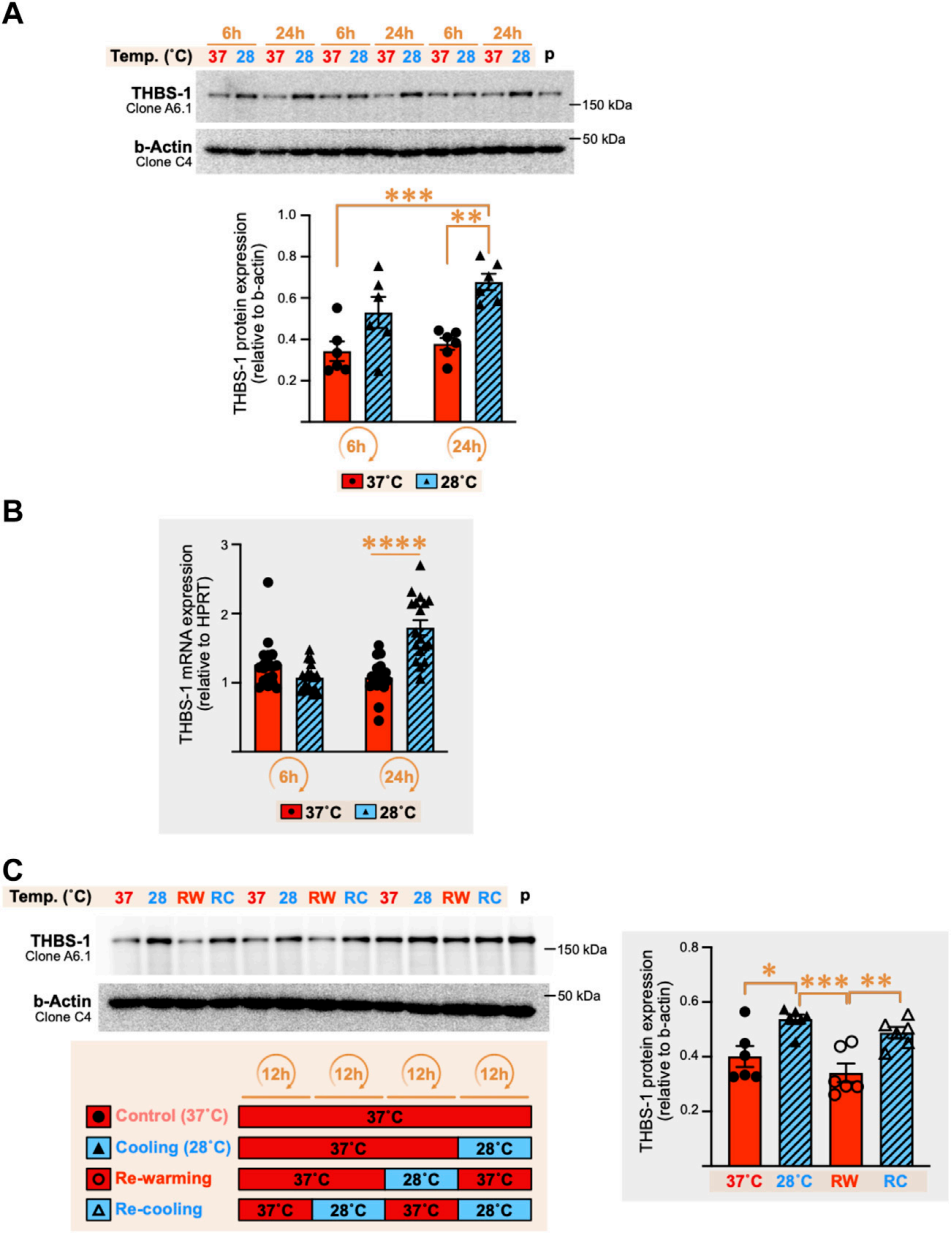
Cold stress exposure increases thrombospondin-1 mRNA and protein expression in C2C12 myotubes. **(A)** Representative immunoblots and densitometry analyses for cellular THBS-1 protein levels in C2C12 myotubes exposed for 6 h and 24 h to cold stress (28°C) or maintained at 37°C. β-actin is used as a loading control. p, pool of all sample loaded as a calibrator for inter-gel comparison when required. Significant difference between groups: **, *p* ≤ 0.01; ***, *p* ≤ 0.001. Data is represented as means ± SEM from three independent experiments, each with *n* = 6 samples per condition of exposure and temperature. **(B)** THBS-1 mRNA expression levels in C2C12 myotubes exposed to 37°C or 28°C for 6 h or 24 h. Hypoxanthinephosphoribosyltransferase-1 (HPRT) is used as a housekeeping gene. Significant differences: ****, *p* ≤ 0.0001. Data is represented as means ± SEM from three independent experiments, each with *n* = 6 samples per condition of exposure and temperature. **(C)** Representative immunoblots and densitometry analyses for cellular THBS-1 protein expression levels in C2C12 myotubes maintained at 37°C (Control), exposed for 12 h to cold (Cooling, 28°C), subjected to 12 h re-warming (RW) post-cold exposure, or 12 h re-cooling (RC) post-rewarming. β-actin is used as a loading control. p, pool of all sample loaded as a calibrator for inter-gel comparison when required. Significant differences: *, *p* ≤ 0.05; **, *p* ≤ 0.01, ***, *p* ≤ 0.001. Data is represented as means ± SEM from two independent experiments, each with *n* = 6 samples per condition.

The increase in cellular THBS-1 expression in response to cold stress prompted us to explore further whether this could be a true physiological response to cold or simply an alteration of THBS-1 secretion in the extracellular environment. Three independent experiments (*n* = 6 culture dishes in each) were conducted with C2C12 myotubes exposed to 28°C or 37°C for 6 h and 24 h, and cellular THBS-1 protein levels were measured (Figure 4A). Confirming the results obtained on our first set of samples (Figure 3), cellular THBS-1 protein expression was strongly increased by 80–120% after 24 h of exposure to cold stress (+79%, *p* = 0.0023; +126%, *p* ≤ 0.0001; +99%, *p* = 0.0003 in three independent experiments) whereas a shorter cold incubation (6 h) resulted in a modest increase or just in a trend for an increase (Figure 4A: +54%, ns; +39%, *p* = 0.0249; +42%, ns).

mRNA levels for *THBS-1* gene expression were significantly increased after 24 h of cold stress exposure by respectively +72% (*p* = 0.04), +111% (*p* ≤ 0.0001), and +28% (*p* = 0.0003) in three independent experiments, whereas no significant change was observed at 6 h (Figure 4B).

### 3.4 The effect of cold stress exposure on cellular THBS-1 protein expression in C2C12 myotubes is reversible and re-inducible

The stimulatory effect of cold on cellular THBS-1 protein expression in mouse C2C12 myotubes appears as a robust physiological response. We wondered whether this response was retained when cycles of cooling were repeated. In these experiments, C2C12 myotubes were cultured for an overall duration of 36 h in all groups to ensure cells were all collected after the same duration of culture. Lowering the temperature from 37°C to 28°C for only 12 h resulted in a significant increase by 34–52% in cellular THBS-1 protein levels in two independent experiments (*p* = 0.0077, and *p* = 0.0167, see representative experiment in Figure 4C). Re-warming the cells for 12 h at 37°C (RW) after cooling (12 h of 28°C) reversed the cold-induced increase in THBS-1 protein (*p* = 0.0006 and *p* = 0.0047 in two independent experiments, Figure 4C). Finally, the cellular expression of THBS-1 was higher after an episode of re-cooling (RC) for 12 h at 28°C post re-warming (Figure 4C, +43% (*p* = 0.0092) and +27% (*p* = 0.2996) in two independent experiments). This suggested that cold retained the capacity to increase the cellular level of THBS1 after two cycles of cooling.

### 3.5 Screening for angioadaptive proteins in C2C12 myotubes exposed to cold stress is unconclusive

**FIGURE 5:**
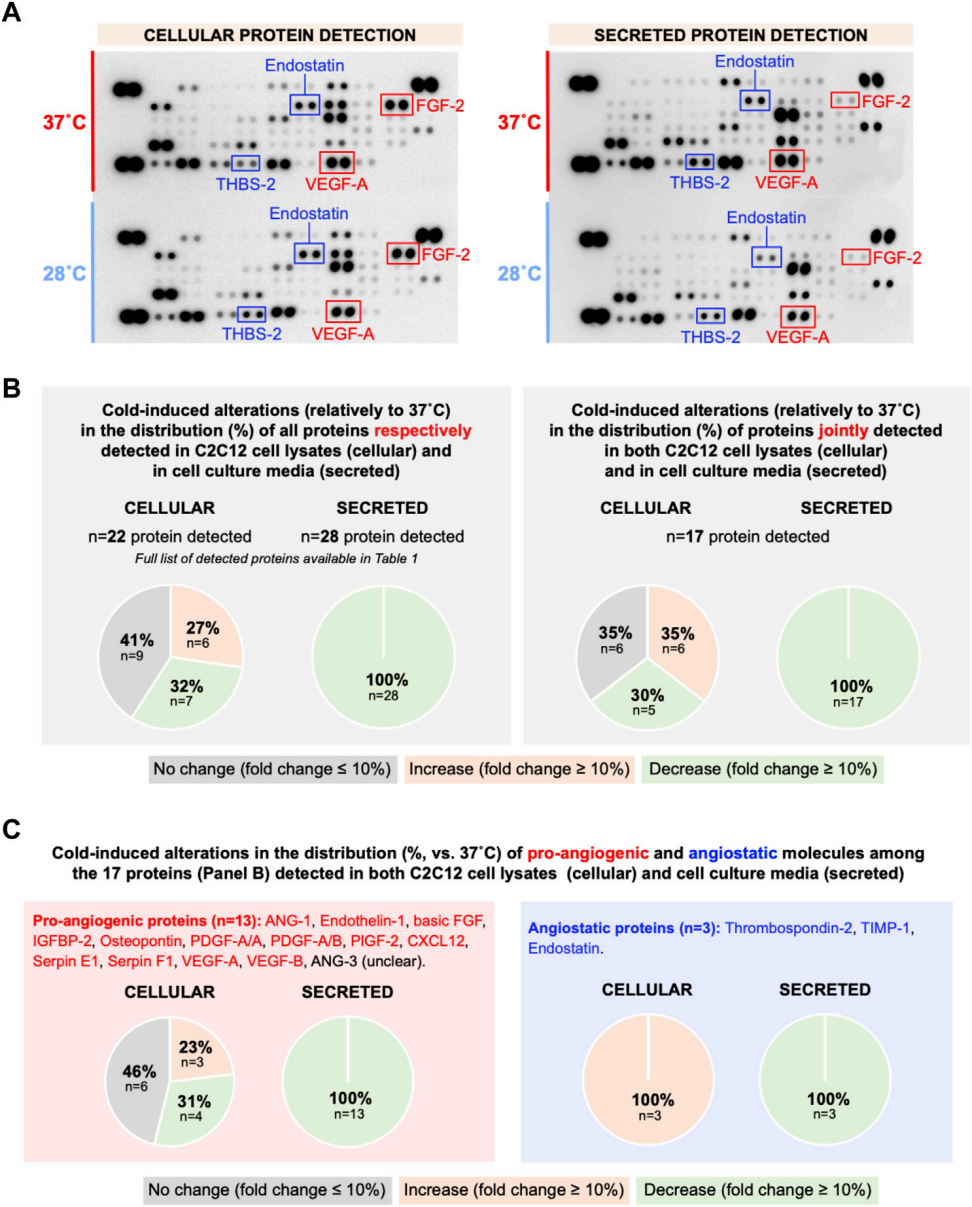
Effect of cold stress exposure on cellular and secreted protein levels of pro- and angiostatic molecules in C2C12 myotubes. **(A)** Representative proteome profiler immunoblotting membranes for cell lysates (cellular proteins) and cell media samples (secreted proteins) from mouse C2C12 myotubes exposed for 24 h to 37°C or 28°C. The angiostatic endostatin and thrombospondin-2 (THBS-2) and the pro-angiogenic vascular endothelial growth factor-A (VEGF-A) and fibroblast growth factor-2 (FGF-2) are highlighted. Each highlighted square shows duplicate spots for a given protein. **(B)** Distribution in percentage of cold-induced alterations in proteins detected in the proteome profiler array in cell lysates and cell media samples from C2C12. **(C)** Specific distribution in percentage of cold-induced alterations in pro-angiogenic and angiostatic proteins detected in cell lysates and cell media samples from C2C12.

Whether cold stress promotes an angiostatic or a pro-angiogenic microenvironment in the extracellular compartment of C2C12 myotube culture could not be answered from VEGF-A and THBS-1 measurements, the two showing reduced expression levels (Figures 2–4). Using these same set of samples presented in Figures 2–3 where both VEGF-A and THBS-1 were quantified, we screened for 53 angiokines using an angiogenesis proteome profiler array (Figure 5A). A total of 22 cellular and 28 secreted proteins were detected respectively (Figure 5B and Table 1). Among these proteins, 17 were found in both cell lysate (cellular) and cell media (secreted) samples. We distinguished increases or decreases with the magnitude of change between 37°C and 28°C exceeding +/-10% (Figure 5B; Table 1). Interestingly, whereas cellular angiokines showed a balanced distribution between no change, increase, or decrease, all angiokines detected in cell media samples had their expression levels reduced with cold exposure (Figure 5B). In Figure 5C, we distinguished angiokines based on their pro-angiogenic or angiostatic properties. Pro-angiogenic molecules showed a balance distribution at the cellular level and 100% of them revealed reduced expression levels among secreted proteins (Figure 5C). When considering angiostatic molecules (endostatin, TIMP-1 and THBS-2), all cellular levels increased and all secreted levels decreased (Figure 5C), similarly to the observations made for THBS-1. Finally, VEGF-A was also present on the proteome array, with respectively no change (5–6%) in cell lysate samples and lower expression levels (-50%) in cell media samples (Table 1), an observation in accordance with our ELISA measurements (Figure 2).

**TABLE 1:** Angiokines expression in cell lysates (cellular proteins) and cell culture media (secreted proteins) from C2C12 myotubes exposed to cold.

**Proteins detected in C2C12 cell lysate**	**Cold-induced changes (in % *versus* 37°C)**	**Proteins detected in C2C12 cell media**	**Cold-induced changes (in % *versus* 37°C)**
Angiopoientin-1	+29.27	Angiopoietin-1	−30.01
Endostatin	+29.68	Angiopoietin-3	−25.44
IGFBP-2	+45.06	CXCL16	−25.25
CXCL12	+10.08	Endostatin	−45.94
THBS-2	+81.70	Endothelin-1	−18.10
IMP-1	+29.64	Basic FGF	−30.34
Angiopoietin-3	−29.13	CX3CL1	−27.35
HB-EGF	−24.85	HGF	−25.45
NOV	−10.01	IGFBP-2	−15.79
PDGF-A/B	−16.73	IGFBP-3	−41.93
PIGF-2	−35.35	IL-1a	−32.82
Serpin F1	−25.91	MCP-1	−42.28
VEGF-B	−41.43	MMP-8	−28.55
ADAMTS-1	−7.01	MMP-9	−37.62
Coagulation factor-3	−0.80	NOV	−48.02
Cyr61	+6.81	Osteopontin	−18.51
Endothelin-1	−7.46	PDGF-A/A	−45.23
Basic FGF	+0.08	PDGF-A/B	−42.63
Osteopontin	−0.73	PlGF-2	−60.16
PDGF-A/A	−6.45	Prolactin	−37.68
Serpin E1	+3.72	Proliferin	−35.78
VEGF-A	−5.67	CXCL12	−20.50
		Serpin E1	−24.32
Colour coding		Serpin F1	−40.11
Change ≤10%		THBS-2	−42.76
Increase (fold change ≥10%)		TIMP-1	−17.17
Decrease (fold change ≥10%)		VEGF-A	−50.04
		VEGF-B	−38.71

### 3.6 Cold-conditioned C2C12 cell media does not alter primary skeletal muscle endothelial cells migratory activity

**FIGURE 6:**
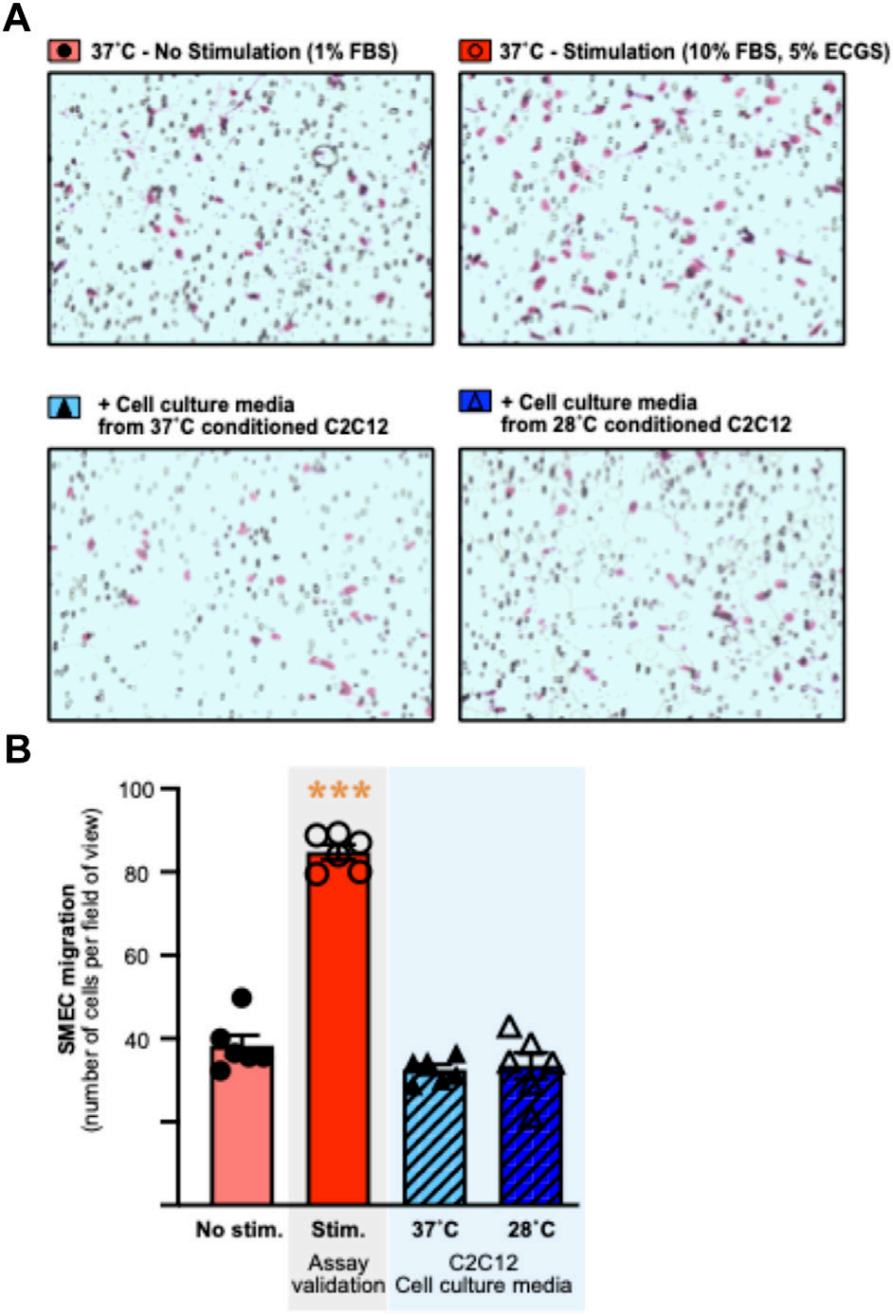
Culture media from cold-conditioned C2C12 myotubes does not alter the migratory activity of primary mouse skeletal muscle endothelial cells. Primary mouse skeletal muscle endothelial cells (SMECs) migratory activity was assessed in the Boyden chamber chemotaxis assay. **(A)** Representative pictures of the Boyden chamber chemotaxis assay in response to non-stimulatory (1% Fetal Bovine Serum (FBS)) and stimulatory (10% FBS, 5% Endothelial Cell Growth Supplement (ECGS)) conditions, and stimulation with cell culture media from 37°C or 28°C conditioned C2C12. SMECs are visualized and counted after Giemsa staining. **(B)** Representative histogram of SMECs migration, assessed by number of cells per field of view, in response to experimental conditions. Data is represented as means ± SEM of a single experiment. Six independent experiments were conducted with *n* = 6 migration wells per condition. Significant effect of stimulation: ***, *p* ≤ 0.001.

Since cold resulted in lower secreted levels of all angiokines measured, our proteome approach did not bring any consensus regarding whether cold stress would promote a pro-angiogenic or angiostatic extracellular microenvironment. We therefore aimed to address whether the cold-induced alterations in C2C12 secretion of angiokines could alter the migratory activity of primary skeletal muscle endothelial cells (SMECs). SMECs were stimulated *in vitro* in the Boyden chamber assay with cell media samples collected from C2C12 exposed for 24 h to 37°C or 28°C. Six independent Boyden chamber experiments were conducted as previously described (with *n* = 6 chemotaxis wells for each condition and independent experiment) (Aiken et al., 2016). The validity of each experiment was assessed by incorporating a positive control condition (no stimulation vs. stimulation with FBS and ECGS) (Figure 6A). In all 6 experiments, migration was greater in cells stimulated with 10%FBS and 5% ECGS (positive control), validating the assays (Figure 6AB: +60 to +122%, significant at *p* ≤ 0.001 in EXP3 and *p* ≤ 0.0001 in EXP1-2-4-5-6). The cold-conditioned C2C12 cell media had no significant impact on SMEC migratory activity (Figure 6A,B).

### 3.7 Cold increases the cellular levels of RBM3 protein in primary skeletal muscle endothelial cells and stops their proliferation

**FIGURE 7:**
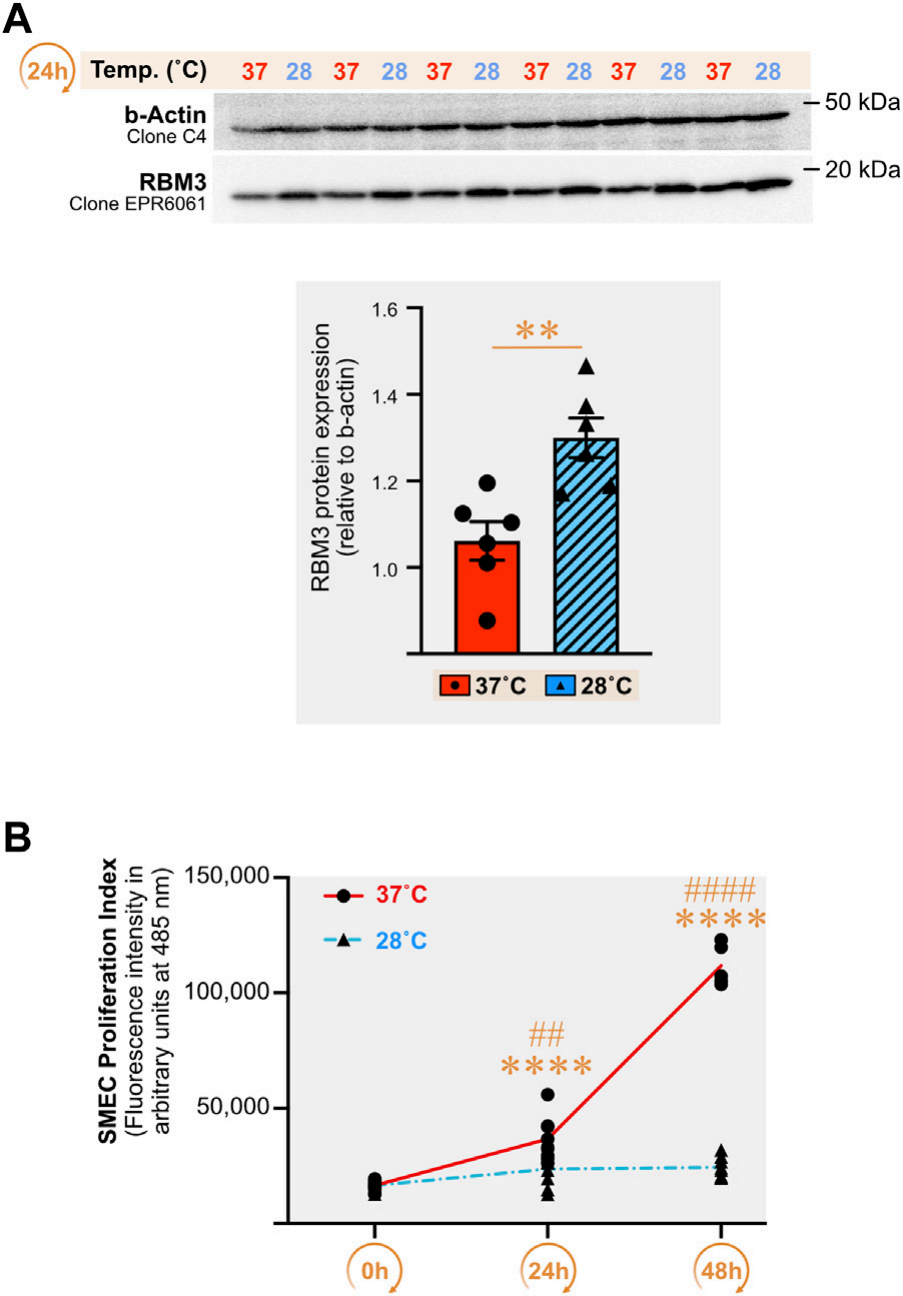
Direct cold stress exposure increases RNA-binding motif 3 protein expression in primary mouse skeletal muscle endothelial cells and inhibits cell proliferation. **(A)** Representative immunoblots and densitometry analyses for cellular protein levels of the cold-shock RNA-binding motif 3 protein (RBM3) in primary mouse skeletal muscle endothelial cells (SMECs) exposed for 24 h to cold stress (28°C) or maintained at 37°C. β-actin is used as a loading control. Significant difference between groups: **, *p* ≤ 0.01. Data is represented as means ± SEM from three independent experiments, each with *n* = 6 samples per condition of exposure and temperature. **(B)** SMECs proliferation at 37°C and 28°C was quantified at Days 0, 1 and 2. Data illustrates one representative proliferation assay out of three independent experiments with *n* = 4–8 samples per time-point in each. Significantly different from Day 0: ****, *p* ≤ 0.0001. Significantly different from the corresponding 28°C time-point measurement: ##, *p* ≤ 0.01; ####, *p* ≤ 0.0001.

Direct cold exposure (24 h at 28°C) led to a higher expression of the cold biomarker RBM3 protein in SMECs (Figure 7A, +14% (*p* = 0.03), +23% (*p* = 0.004), and +18% (*p* = 0.06) in three independent experiments. We assessed the impact of 24h and 48 h of cold exposure on SMECs proliferation (*n* = 3 independent experiments with n = 4–8 samples per condition) (Figure 7B). Whereas 37°C-cultivated cells proliferated normally, SMEC cultivated at 28°C showed a complete loss of their proliferative capacity.

### 3.8 Cold pre-conditioning of skeletal muscle endothelial cells increases their migratory responsiveness but does not affect cell proliferation

**FIGURE 8:**
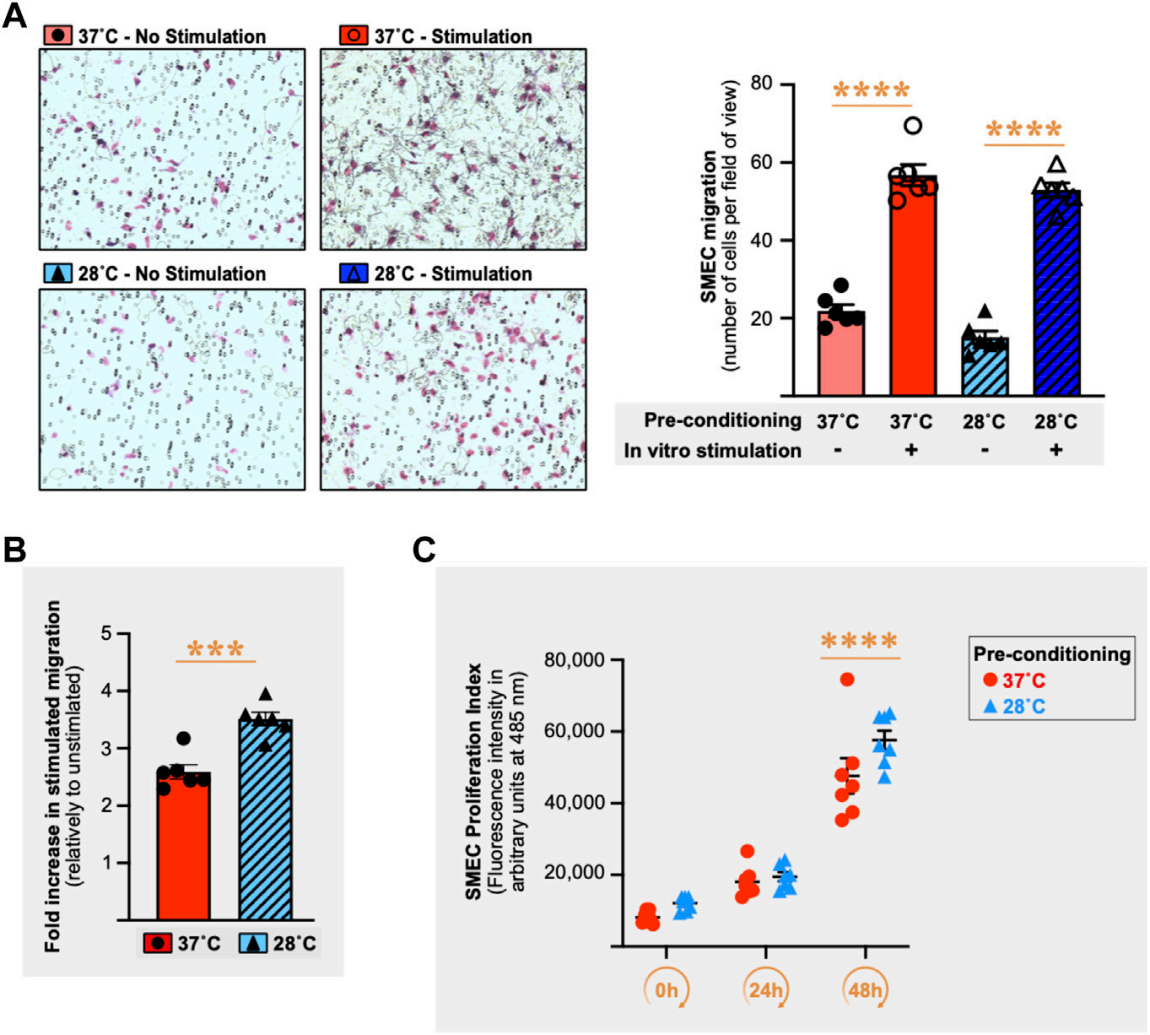
Cold stress pre-conditioning enhances migratory responsiveness in primary mouse skeletal muscle endothelial cells. **(A)** Left panel shows representative pictures of primary mouse skeletal muscle endothelial cells (SMECs) migration in the Boyden chamber chemotaxis assay. SMECs were pre-conditioned for 16 h at 37°C or 28°C prior to 6 h incubation in the Boyden chamber at 37°C with or without stimulation (10% Fetal Bovine Serum (FBS) + 5% Endothelial Cell Growth Supplement (ECGS) *versus* 1% FBS only), cold pre-conditioning (28°C) with or without *in vitro* stimulation (10% FBS +5% ECGS *versus* 1% FBS). Right panel histograms show one representative Boyden chamber assay experiment (EXP3) out of the five performed. Data is expressed as number of cells per field of view. Data is represented as means ± SEM (*n* = 6 migration wells per condition). Significant difference between non-stimulation and stimulation: ****, *p* ≤ 0.0001. **(B)** Histogram showing the difference between 37°C and 28°C conditions for the fold increase in stimulated migration relative to unstimulated migration for the representative EXP3 shown in Panel **(A)**. Significant difference in fold increase in stimulated migration between 37°C and 28°C: ***, *p* ≤ 0.001. Data is represented as means ± SEM (n = 6 migration wells per condition). **(C)** SMECs proliferation following 16 h of pre-conditioning at either 37°C or 28°C. Cell proliferation was quantified at Days 0, 1, and 2. Data illustrates one representative proliferation assay out of 3 independent experiments with *n* = 6–7 samples per time-point in each. Significantly different from Day 0: ****, *p* ≤ 0.0001.

Next, we tested whether cold pre-conditioning could impact endothelial migratory capacity. SMECs migratory activity was assessed *in vitro* in the Boyden chemotaxis assay (6 h migration at 37°C) after cell pre-conditioning for 16 h at 28°C or 37°C. Figure 8A shows a representative Boyden chemotaxis assay from *n* = 5 independent experiments (*n* = 6 chemotaxis wells per condition and per experiment). In the absence of stimulation, SMECs had similar basal migration capacity when pre-conditioned at 28°C or 37°C (Figure 8A). Stimulation of SMECs increased the number of migrating cells by 259% when pre-conditioning was done at 37°C and by +351% with a pre-conditioning at 28°C (Figure 8B, *p* ≤ 0.0001 for both). When expressing stimulated migration as the fold of change compared to no stimulation, the 28°C-conditioned SMECs displayed a significant increase in their migratory responsiveness compared to 37°C-conditioned cells (Figure 8B) in 4 out of the 5 independent Boyden assays (+17%, *p* = 0.007; +49%, *p* ≤ 0.0001; +36%, *p* = 0.0003; +45%, *p* = 0.0002; +13%, *p* = 0.054). We next assessed if cold pre-conditioning (16 h at 28°C or 37°C) could also affect endothelial cell proliferation. Figure 8C shows a representative experiment from the three performed independently (n = 6-7 cell culture wells per condition per experiment). Unlike migration, cold pre-conditioning did not affect SMECs proliferation.

### 3.9 Cold stress exposure alters VEGF receptor-2 expression and responsiveness to VEGF-A in skeletal muscle endothelial cells

**FIGURE 9:**
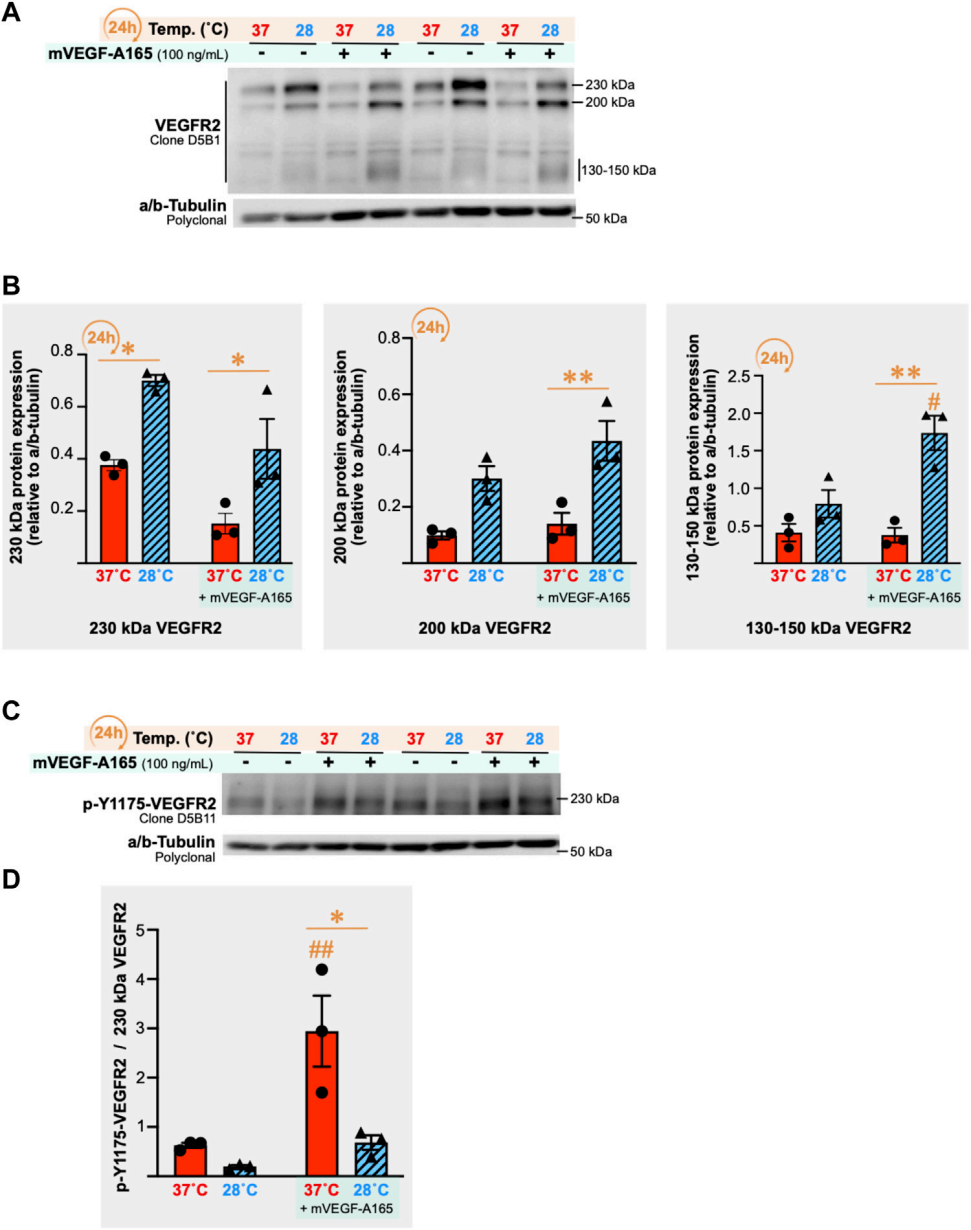
Impact of cold stress exposure on VEGF receptor-2 expression and phosphorylation in primary mouse skeletal muscle endothelial cells. **(A)** Representative immunoblots for VEGF receptor-2 (VEGFR2) protein expression in primary mouse skeletal muscle endothelial cells (SMECs) following 24 h exposure to 37°C or 28°C, with or without stimulation with mouse recombinant VEGF-A_165_ (mVEGF-165A, 100 ng/ml, 1 h). α/β-Tubulin was used as a loading control. **(B)** Densitometry analyses for the 230 kDa, 200 kDa, and 130–150 kDa forms of the VEGFR2 detected in **(A)**. Histograms are of a single independent experiment representative of three independently conducted experiments (*n* = 3 samples per condition in each). Significant difference between 37°C and 28°C: *, *P* ≤ 0.05; **, *p* ≤ 0.01. Significant difference between -mVEGF-A165 and +mVEGF-A165: #, *p* ≤ 0.05. Data is represented as means ± SEM (*n* = 3 samples per condition). **(C)** Representative immunoblots for VEGFR2 phosphorylated on residue tyrosine 1175 (p-Y1175-VEGFR2) in samples described in **(A)**. α/β-Tubulin was used as a loading control. **(D)** Densitometry analyses for p-Y1175-VEGFR2 protein expression normalized to the 230 kDa form of VEGFR2 in samples presented in panels (A, 230 kDa VEGFR2) and (C, p-Y1175-VEGFR2). Histograms are a single experiment that is representative of three independent experiments conducted (with *n* = 3 samples per condition). Significant difference between 37°C and 28°C: *, *p* ≤ 0.05. Significant difference between -mVEGF-A165 and +mVEGF-A165: ##, *p* ≤ 0.01. Data is represented as means ± SEM (*n* = 3 samples per condition).

VEGF receptor-2 (VEGFR2) signaling plays an important role in promoting endothelial cell proliferation and migration, two important steps of the angiogenic process (Wang et al., 2020). We measured VEGFR2 protein expression in SMECs after 24 h of cold stress exposure (28°C) with and without stimulation for 1 h with recombinant murine VEGF-A_165_ (mVEGF-A165, 100 ng/ml) in three independent experiments (*n* = 3 per condition per experiment). Multiple bands were detected when immunoblotting for VEGFR2 (Figure 9A). All VEGFR2 bands showed higher expression levels after 24 h of cold stress exposure (Figure 9B). From the three detected VEGFR2 forms, the 230 kDa form is described as the only one expressed in the cell membrane to be phosphorylated by VEGF-A (Takahashi and Shibuya, 1997). Next, VEGFR2 responsiveness to mVEGF-A165 stimulation was assessed by measuring the phosphorylation of the receptor on its tyrosine residue 1175 (p-Y1175-VEGFR2) (Figures 9C,D). Cold decreased the ratio of p-Y1175-VEGFR2 signal to 230 kDa VEGFR2 signal in VEGF-A stimulated SMECs (-30%, n.s.; -77%, *p* = 0.0006; and -76%, *p* = 0.0107).

### 3.10 *Ex-vivo* incubation of skeletal muscle fragments confirms that cold upregulates cellular levels of THBS1 despite no cold-induced inhibition of angiokine secretion

**FIGURE 10:**
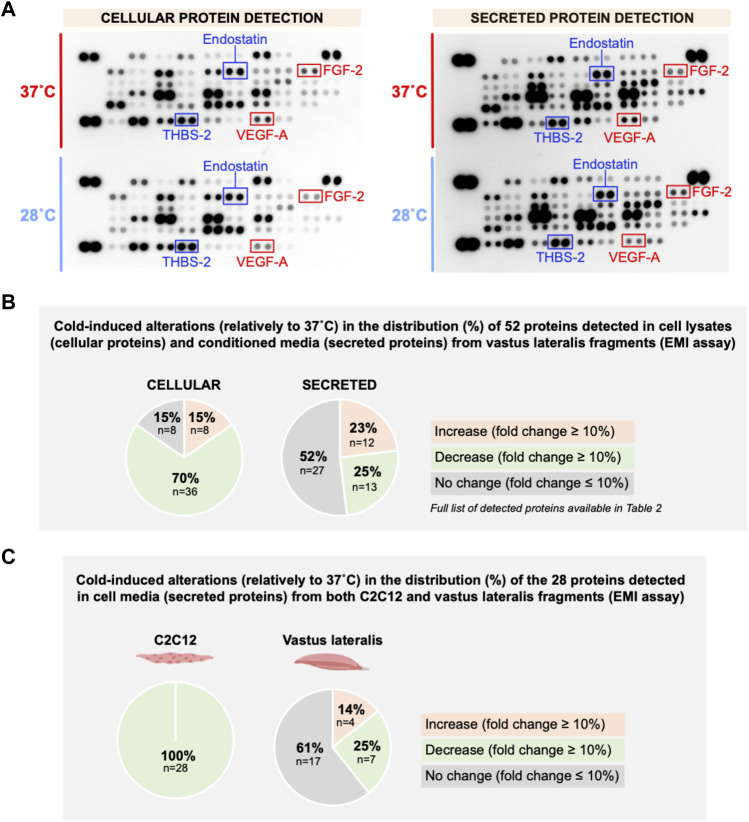
*In vitro* effect of cold stress exposure on cellular and secreted angioadaptive protein expression levels in mouse vastus lateralis muscle fragments (EMI assay). **(A)** Representative proteome profiler immunoblotting membranes for cellular and secreted angiokines from mouse vastus lateralis muscle fragments incubated for 24 h at 37°C or 28°C (EMI assay). The angiostatic endostatin and thrombospondin-2 (THBS-2) and the pro-angiogenic vascular endothelial growth factor-A (VEGF-A) and fibroblast growth factor-2 (FGF-2) are highlighted. Each highlighted square shows duplicate spots for a given protein. **(B)** Distribution in percentage of cold-induced alterations in proteins detected in the proteome profiler array in EMI muscle cell lysates (cellular proteins) and conditioned media (secreted proteins). **(C)** Distribution in percentage of cold-induced alterations in the 28 secreted proteins detected in both C2C12 (Figure 5) and EMI muscle.

**FIGURE 11:**
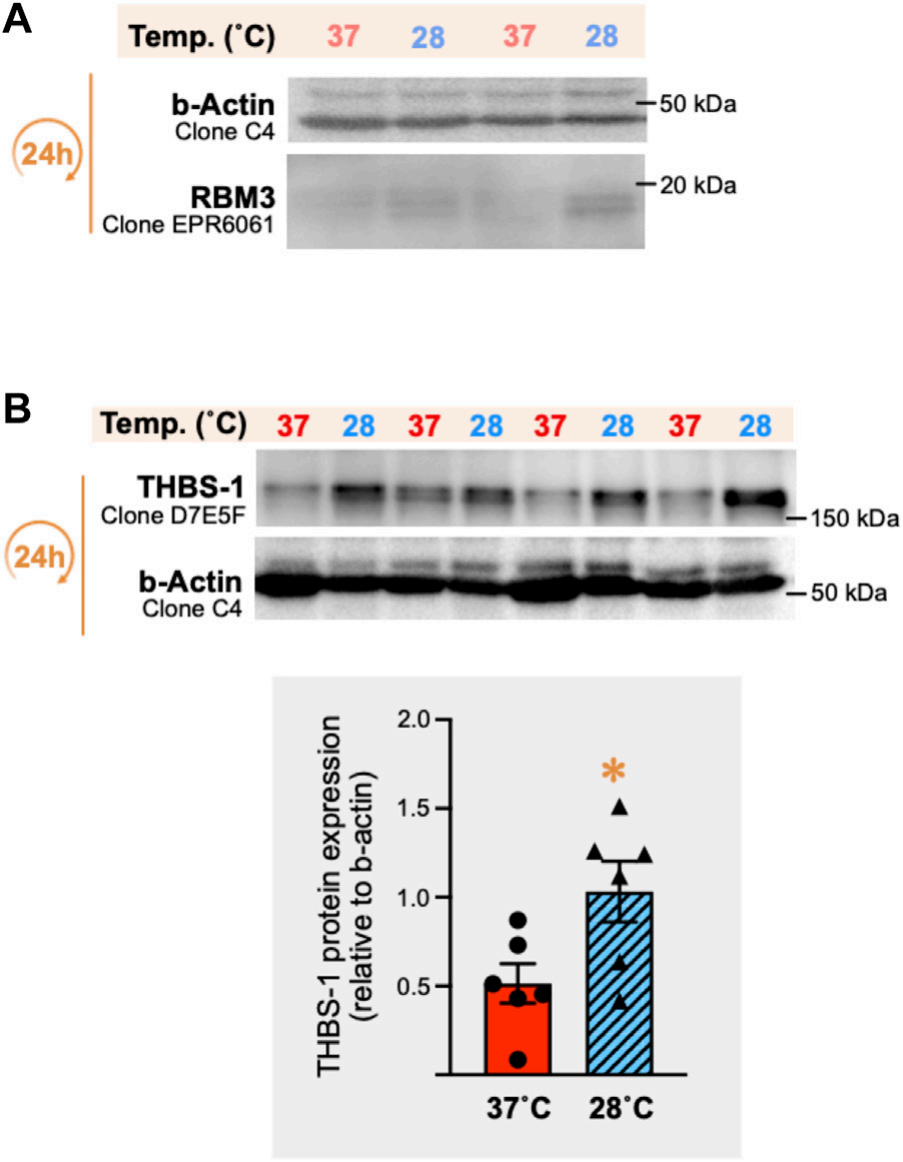
*In vitro* cold stress exposure increases RBM3 and thrombospondin-1 protein expression in mouse vastus lateralis muscle biopsies. **(A)** Representative immunoblots for the cellular expression of the cold-shock RNA-binding motif 3 protein (RBM3) in mouse vastus lateralis muscle fragments (EMI assay) incubated for 24 h *in vitro* at 37°C or 28°C. β-actin was used as a loading control. **(B)** Representative immunoblots and densitometry analysis of thrombospondin-1 (THBS-1) protein expression in these same muscle samples. β-actin was used as a loading control. Significant difference between 37°C and 28°C: *, *p* ≤ 0.05 (data represented are means ± SEM from *n* = 6 samples).

To gain insight to the angioadaptive impact of cold stress on the skeletal muscle tissue, we screened for 53 angiokines using the angiogenesis proteome array on mouse vastus lateralis biopsies exposed *in vitro* to 37°C or 28°C for 24 h (*ex vivo* muscle incubation (EMI) assay, Figure 10A). A total of 52 out of 53 proteins were detected both in muscle cell lysates and conditioned media (Figure 10B; Table 2). At the cellular level, we observed a mixed distribution with 15% of proteins showing an increase or no change, and 70% showing a lower expression (Figure 10B). A mixed distribution of secreted proteins was more pronounced in conditioned media with 23% of proteins increasing, 25% decreasing, and 52% showing no change (Figure 10B). Among these secreted proteins, 28 were found both in muscle biopsies (Figure 10) and C2C12 (Figure 5). The mixed distribution of these 28 secreted proteins in muscle biopsies contrasts with C2C12 where they were all decreased (Figure 10C). To ensure the effect of our cold stress conditioning in our vastus lateralis EMI model, we qualitatively assessed cellular protein levels for RBM3 (Figure 11A). Finally, thrombospondin-1 (THBS-1) is not present on the proteome array. The quantification of its expression level in EMI samples by immunoblotting showed 100% increase in cold-conditioned muscle biopsies (Figure 11B, *p* = 0.029).

**TABLE 2:** Angiokines expression in cell lysates (cellular proteins) and cell media (secreted proteins) from mouse vastus lateralis muscle fragments (EMI assay) exposed to cold.

Proteins detected in vastus lateralis cell lysate	Cold-induced changes (in % *versus* 37°C)	Proteins detected in vastus lateralis cell media	Cold-induced changes (in % *versus* 37°C)
ADAMTS-1	+35.53	ADAMTS-1	+34.64
Angiogenin	+33.42	Angiogenin	+13.25
Coagulation factor-3	+20.35	Cyr61	+36.35
Endogolin	+23.80	Acidic FGF	+14.85
Pentraxin-3	+42.00	Basic FGF	+21.22
Platelet factor-4	+19.19	FGF7	+12.91
Serpin F1	+17.85	IGFBP-2	+15.82
THBS-2	+32.32	KC	+12.92
Angiopoietin-3	−12.12	Leptin	+21.51
CXCL16	−41.92	NOV	+10.48
DLL4	−28.01	Platelet factor-4	+36.39
EGF	−13.38	THBS-2	+29.59
Endothelin-1	−29.25	Endoglin	−10.40
Acidic FGF	−41.55	GM-CSF	−28.97
Basic FGF	−35.49	IP-10	−12.43
FGF7	−28.84	MIP-1a	−11.11
Fractalkine	−26.46	MMP-3	−16.34
GM-CSF	−10.73	MMP-8	−20.70
HB-EGF	−30.63	MMP-9	−13.64
HGF	−26.11	Osteopontin	−52.63
IGFBP-1	−30.93	Pentraxin-3	−10.95
IGFBP-2	−42.61	PIGF-2	−55.79
IGFBP-3	−22.51	Serpin E1	−48.45
IL-1a	−55.25	TIMP-1	−46.31
IL-10	−32.75	VEGF-A	−34.59
Leptin	−23.09	Amphiregulin	+6.79
MIP-1a	−20.11	Angiopoietin-1	+1.11
MMP-3	−29.01	Angiopoietin-3	+3.28
MMP-8	−52.45	Coagulation factor-3	+6.01
MMP-9	−51.83	CXCL16	−5.19
NOV	−23.56	DLL4	+7.58
Osteopontin	−66.7	CD26	+3.62
PD-ECGF	−36.95	EGF	+6.95
PDGF-AA	−33.95	Endostatin	−8.27
PDGF-AB/PDGF-BB	−15.16	Endothelin-1	−1.58
PIGF-2	−51.82	Fractalkine	+2.24
Prolactin	−53.89	HB-EGF	+7.29
Proliferin	−55.20	HGF	+4.21
SDF-1	−24.26	IGFBP-1	+4.86
Serpin E1	−21.54	IGFBP-3	−1.69
TIMP-1	−56.96	IL-1a	−6.06
TIMP-4	−35.57	IL-10	−2.39
VEGF-A	−30.68	MCP-1	+0.26
VEGF-B	−57.66	PD-ECGF	−6.63
Amphiregulin	+2.60	PDGF-AA	+6.77
Angiopoietin-1	+5.13	PDGF-AB/PDGF-BB	+3.29
Cyr61	−1.48	Prolactin	+1.22
CD26	−8.60	Proliferin	−3.51
Endostatin	+5.95	SDF-1	+2.85
IP-10	+8.88	Serpin F1	−2.34
KC	−3.23	TIMP-4	+3.37
MCP-1	+4.73	VEGF-B	+0.74
Colour coding			
Change ≤10%			
Increase (fold change ≥10%)			
Decrease (fold change ≥10%)			

## 4 Discussion

Here, we evaluated how cold stress *per se* might impact endothelial cells by modulating the release of angiokines from muscle cells, and we compared this indirect effect to the direct exposure of endothelial cells to cold. Our results identified cold stress as a powerful tool to hinder the secretion of angiokines by myotubes. Yet, this did not shift angioadaption. Pro-angiogenic and angiostatic molecules were equally affected, and the secretome from cold-treated myotubes had no impact on endothelial cell migration. Conversely, the direct exposure of primary skeletal muscle endothelial cells to cold blunted their proliferation despite higher protein levels of VEGFR2. Interestingly, when pre-cooled endothelial cells were cultured back into a warm environment, they proliferated similarly to control warm pre-conditioned cells but displayed a stronger migratory response to pro-angiogenic stimuli. This pre-cooling effect contrasts with direct cold exposure. If cold “freezes” the myotubes-specific secretion of angiokines and SMECs proliferation, pre-cooling could “prime” endothelial cells for greater migratory responsiveness. Based on these observations, cold could then exert an intriguing dichotomic angioadaptive effect, from “angiostatic freeze to angiogenic move” during cold-rewarming cycles. Although being appealing, this perspective is however based on SMECs migration *in vitro* in a Boyden chamber assay. Future research should investigate the pro-angiogenic potential of pre-cooling conditioning by using more functional assays such as evaluating SMECs migration and vascular tube formation in a tri-dimensional matrigel assay *in vitro* and *in vivo*.

To assess the impact of cold on muscle angioadaptation, several studies have analyzed VEGF-A expression in whole cardiac or skeletal muscle tissue in rodents or human subjects exposed to cold (Kim et al., 2005; Ihsan et al., 2014; Joo et al., 2016; D’Souza et al., 2018; Al-horani et al., 2019; Opichka et al., 2019; Magalhães et al., 2020; Shute et al., 2020). Most of these studies combined cold and exercise in the context of cold acclimatization or in the context of post-exercise cooling strategies. This resulted in discrepancy with some studies showing no impact of cold on VEGF-A expression (Ihsan et al., 2014; Opichka et al., 2019; Magalhães et al., 2020; Shute et al., 2020) and others reporting higher protein expression mostly when combining exercise and post-exercise cooling (Joo et al., 2016; D’Souza et al., 2018; Al-horani et al., 2019). This highlights the need to design well-controlled experiments to ascertain the impact of cold *per se* on muscle-specific angiokines and more broadly on its angioadaptation to this environmental stressor.

In our study, we have investigated the impact of cold on a broader range of angiokines combining *ex vivo* and *in vitro* models with well-controlled temperatures. Intramuscular temperature can vary significantly based on muscle type, cooling protocol, temperature measurement methodology, depth of tissue insertion of the thermal probe (Barcroft and Edholm, 1946; Clarke et al., 1958; Ihsan et al., 2014; Castellani and Young, 2016; Cuthbert et al., 2019; Freitag et al., 2021). We chose to drop our cell culture temperature by 9°C based on previous studies documenting drops in human intramuscular temperature from 37–38°C to 28–30°C after cold water immersion (Barcroft and Edholm, 1946; Ihsan et al., 2014). Although the RNA-binding motif protein-3 (RBM3) was reported to also sense hypoxia and contractile activity (Wellmann et al., 2004; Zhu et al., 2016; Cuthbert et al., 2019), it was reported as a good marker of cold stress in C2C12 myoblasts (Cuthbert et al., 2019), in the skeletal muscle of hibernating black bears facing extreme environmental temperatures (Fedorov et al., 2009), as well as in human endothelial cells (Zieger et al., 2011). Here, the 9°C drop in temperature efficiently increased the expression of RBM3 in C2C12 myotubes, primary skeletal muscle endothelial cells, and vastus lateralis biopsies, confirming that our experimental conditions elicited a cold stress response.

To our knowledge, this work represents the first study to simultaneously measure intracellular and secreted angiokines in cultured myotubes exposed to cold, and the first to characterize THBS-1 expression in this context. Exposing C2C12 myotubes to cold stress led to a similar reduction in VEGF-A and THBS-1 proteins secretion. Changes in cellular protein expression fails to explain this lower expression level. Indeed, cold stress did not alter VEGF-A protein and even significantly increased THBS-1 protein and mRNA within the myotubes. To our knowledge, only two studies have previously analyzed VEGF-A expression in muscle cells (Sugasawa et al., 2016; Krapf et al., 2021). Sugasawa and colleagues exposed rat L6 myoblasts to different cold temperatures (0, 4 and 17°C) and observed higher cellular VEGF-A protein expression only at 4°C (Sugasawa et al., 2016). Krapf and colleagues reported higher VEGF-A mRNA levels but could not detect cellular VEGF-A protein in human myotubes exposed to 37°C or 18°C for 18 h (Krapf et al., 2021). However, these authors reported a decreased expression of secreted VEGF-A protein, in line with our findings. Our proteome array revealed that cold exposure randomly impacted the cellular expression of pro-angiogenic molecules, with some higher or lower expressions levels, or no change in response to cold. Since all pro-angiogenic molecules do not respond similarly to cold, we could question whether cold could recruit multiple pathways to modulate their cellular protein levels. Interestingly, all angiostatic proteins measured in our study, TIMP-1, endostatin, thrombospondin-1 and 2, showed a similar expression pattern to cold, increasing in cell lysate samples and decreasing in cell media samples. The underlying mechanisms of such cold-induced upregulation of angiostatic molecules are unclear. Cold was previously reported to increase cellular THBS-1 protein expression in human coronary arterial endothelial cells (Zieger et al., 2011). We have shown that THBS1 is a transcriptional target of the transcription factor Forkhead box O protein 1 (FoxO1) in the skeletal muscle endothelium (Roudier et al., 2013; Slopack et al., 2014). Interestingly, cold decreases the phosphorylation of FoxO1 on the Ser246 in the muscle tissue, a modification that could stabilize this transcription factor (Manfredi et al., 2013). It would therefore be interesting to investigate in the future if cold could recruit FoxO1 to upregulate THBS1 in muscle cells.

The observations made here regarding the lower secretion of angiokines in C2C12 myotube culture contrasted with our *ex vivo* vastus lateralis muscle biopsies model that showed random increases, decreases and no change in secreted protein levels. These differences could be attributed to our cellular model. Differences can indeed exist in the proteolytic processing and release of angiokines from the extracellular matrix between our two-dimensional model of C2C12 cells and our *ex-vivo* muscle model where the tri-dimensional structure is maintained and where proteases could be released from damaged myofibers during muscle fragments preparation. Whole muscle fragments also contain different cell types. C2C12 myotubes are very distinct from skeletal myofibers that can display various contractile and metabolic phenotypes. Angioadaptive responses, particularly in the context of cold exposure, could vary significantly between muscle-types or fiber-types (Heroux and St Pierre, 1957; Wickler, 1981; Banchero et al., 1985; Suzuki et al., 1997a; Egginton et al., 2001; Deveci and Egginton, 2002; Deveci and Egginton, 2003). Here, we used muscle fragments from mouse vastus lateralis muscles. Several studies have examined the skeletal muscle angioadaptation to cold using this muscle (Snyder et al., 1992; Bae et al., 2003; Ihsan et al., 2014; Joo et al., 2016; D'Souza et al., 2018; Opichka et al., 2019; Magalhães et al., 2020; Shute et al., 2020). Discrepancies exist between these studies, but it remains difficult to discern if this results from specie differences between rodents and humans or from different conditionings.

Regarding the impact of cold stress on the skeletal muscle angioadaptive microenvironment, our intra- and extracellular measurements led to no clear consensus. This agrees with a previous observation from D'Souza and colleagues who have quantified cellular mRNA levels of VEGF-A and the angiostatic SPRED-1 in vastus lateralis biopsies from human subjects engaging in post-exercise active recovery at room temperature or post-exercise cold water immersion (D’Souza et al., 2018). Both VEGF-A and SPRED-1 mRNA levels were increased with cold immersion leaving the question unanswered at the molecular level. Our work brings additional information showing at the cellular level no effect of the secretome from cold-treated myotubes on skeletal muscle endothelial cells migratory activity. This supports the notion that cold stress itself does not promote a pro-angiogenic microenvironment at the level of the myotubes’ secretion of angiokines. Additionally, cold abolished the proliferation of primary skeletal muscle endothelial cells within 24h, and potentially attenuated their responsiveness to VEGF-A. Indeed, signaling through VEGFR2 receptor plays an important role in the angiogenic process (Wang et al., 2020) and the phosphorylated form of VEGFR2 on tyrosine 1175 (p-Y1175-VEGFR2) is critical for VEGFR2 activation and its downstream signalling involved in cell proliferation, migration, and survival (Takahashi, 2001; Wang et al., 2020). Different molecular sizes of the VEGFR2 have been described: a glycosylated 230 kDa form, an intermediated glycosylated 200 kDa form, and a 130–150 kDa non-glycosylated form (Takahashi and Shibuya, 1997; Takahashi, 2001). The 230 kDa form of VEGFR2 has been described as the only one expressed on the cell membrane surface capable of being phosphorylated by VEGF-A (Takahashi and Shibuya, 1997). Here, cold stress decreased the capacity of endothelial cells to phosphorylate VEGFR2 on tyrosine 1175 in response to VEGF-A stimulation, as shown by the changes in the ratio of p-Y1175-VEGFR2 to 230 KDa VEGFR2. This reduced responsiveness to VEGF-A could partly explained the observed impact of cold stress on endothelial cell proliferation. Overall, our study suggests that cold stress might promote angiostasis by “freezing” the secretion of angiokines from myotubes and preventing endothelial cell proliferation.

Since cold decreased the level of all secreted angiokines in C2C12 myotube culture, future research will be needed to understand the physiological relevance, if any, of increasing cellular expression levels of angiostatic molecules in response to cold. These cellular changes might only impact muscle angioadaption after rewarming, when angiokines secretion resumes to post-cold exposure. This hypothesis is supported by some of our results and by the work of other groups.

Krapf et al. (2021) have analyzed secreted VEGF-A protein expression in human myotubes immediately after 18 h of cold exposure as well as after 3 h and 24 h of post-cooling rewarming. Secreted VEGF-A levels were initially decreased by cold exposure, similar to our findings. However, the rate of secretion of VEGF-A in the rewarming recovery period was faster than in control cells. Joo and colleagues have analyzed VEGF-A mRNA expression in vastus lateralis muscle biopsies from subjects exposed to passive cold water immersion and have reported higher VEGF-A mRNA levels after 3 h and 6 h of post-cooling recovery compared to pre-immersion levels (Joo et al., 2016). These studies have focused on the pro-angiogenic VEGF-A and it remains unknown whether angiostatic molecules would also be secreted at a faster rate during rewarming. In our study, we reported that cold exposure resulted in higher expression levels of all molecular forms of VEGFR2 (230, 200, and 130–150 kDa). This could contribute to the greater SMECs migratory response observed during rewarming. Here, SMECs were pre-conditioned to cold at 28°C but cell migration assay was performed at 37°C. Despite similar basal and stimulated migratory levels between control and cold pre-conditioned cells, the migratory responsiveness (fold of change compared to basal migration of corresponding temperature) was strongly enhanced in four out of five assays in cold pre-conditioned SMECs. Interestingly, cold pre-conditioning did not alter SMECs proliferation during rewarming contrasting with the inhibitory effect of direct cold exposure on cell proliferation. We could then hypothesize that cold pre-conditioning might “prime” endothelial cells for greater responsiveness to pro-angiogenic signals when the secretion of angiokines resumes during rewarming.

Altogether, results from our study and from other colleagues suggest that a direct and immediate effect of cold stress exposure on muscle and endothelial cells could rather be angiostatic whereas cold pre-conditioning might conversely enhance pro-angiogenic molecular and cellular responses once returned to warm conditions. Cold is already used as a post-exercise muscle recovering strategy to reduce local inflammation, to limit exercise-induced muscle damages and fatigue, and to restore performance (Bouzigon et al., 2021; D’Souza et al., 2018; Hohenauer et al., 2018; Kwiecien and McHugh, 2021). Here, we suggest that cold could also be considered as a pre-exercise approach to “prime” the skeletal muscle tissue for greater exercise adaptive responses, particularly exercise-induced angiogenesis. Muscle cells might indeed secrete more VEGF-A whereas SMECs might express more VEGFR2 and show better migratory responsiveness. This potential of cold stress to exacerbate the pro-angiogenic stimulus of exercise shares similitudes with the strategy of hypoxia training (Millet et al., 2010; Girard et al., 2013; Lemieux and Birot, 2021).

In conclusion, our study has contributed to better understand how cold, as an environmental stressor, can impact cellular and molecular angioadaptive responses in skeletal muscle tissue. Some of our observations will deserve further investigations to determine for example why angiostatic molecules such as THBS-1 show higher cellular expression levels in muscle cells, what could be the respective contribution of different cell types such as pericytes, myofibers or satellite cells, or whether cold-induced responses differ between muscle types. The most exciting might be to confirm whether cold could serve as a pro-angiogenic pre-conditioner, opening new avenues for athletes’ performance optimization as well as new clinical applications.

## Data Availability

The original contributions presented in the study are included in the article/Supplementary Material, further inquiries can be directed to the corresponding author.
