# Supplementary material for: Angiostatic freeze or angiogenic move? Acute cold stress prevents angiokine secretion from murine myotubes but primes primary endothelial cells for greater migratory capacity
Source: Front Physiol. 2022 Oct 17;13:975652. doi: 10.3389/fphys.2022.975652 (PMC9618727; doi:10.3389/fphys.2022.975652)
Supplement: Supplementary file 1 [file DataSheet1.docx]

Supplementary Material

# Statistical analyses.

| **Figure** | **Experiment Description** | **Experiment #** | **(n) value** | **Mean +/- SEM** | **Statistical method** | **P-value** |
| --- | --- | --- | --- | --- | --- | --- |
| Figure 2A | RBM3 protein expression in C2C12 myotubes cellular fraction.  4 conditions:  **(1) 37˚C-6h**  **(2) 28˚C-6h**  **(3) 37˚C-24h**  **(4) 28˚C-24h** | Experiment 1 | n=6 per condition. | **(1) 37˚C-6h**  1.108±0.065  **(2) 28˚C-6h**  1.149±0.076  **(3) 37˚C-24h**  0.834±0.063  **(4) 28˚C-24h**  1.459±0.067 | Two-way ANOVA  Tukey post hoc | 37˚C-24h v. 28˚C-24h  p<0.0001, **** |
| Figure 2A | RBM3 protein expression in C2C12 myotubes cellular fraction.  4 conditions:  **(1) 37˚C-6h**  **(2) 28˚C-6h**  **(3) 37˚C-24h**  **(4) 28˚C-24h** | **Experiment 2**  **(Representative)** | n=6 per condition. | **(1) 37˚C-6h**  0.537±0.039  **(2) 28˚C-6h**  0.683±0.031  **(3) 37˚C-24h**  0.605±0.072  **(4) 28˚C-24h**  1.152±0.039 | Two-way ANOVA  Tukey post hoc | 37˚C-24h v. 28˚C-24h  p<0.0001,**** |
| Figure 2A | RBM3 protein expression in C2C12 myotubes cellular fraction.  4 conditions:  **(1) 37˚C-6h**  **(2) 28˚C-6h**  **(3) 37˚C-24h**  **(4) 28˚C-24h** | Experiment 3 | n=6 per condition. | **(1) 37˚C-6h**  1.265±0.063  **(2) 28˚C-6h**  1.193±0.045  **(3) 37˚C-24h**  1.207±0.044  **(4) 28˚C-24h**  1.441±0.039 | Two-way ANOVA  Tukey post hoc | 37˚C-24h v. 28˚C-24h  p<0.0092,** |
| Figure 2B | VEGF-A protein expression in C2C12 myotubes cellular fraction.  2 conditions:  **(1) 37˚C-24h**  **(2) 28˚C-24h** | **Experiment 1**  **(Representative)** | n=6 per condition. | **(1) 37˚C-24h**  9.212±0.2346  **(2) 28˚C-24h**  9.449±0.3392 | Unpaired student’s  t-test | 37˚C-24h v. 28˚C-24h  p=0.5791, n.s |
| Figure 2C | VEGF-A protein expression in C2C12 myotubes secreted fraction.  2 conditions:  **(1) 37˚C-24h**  **(2) 28˚C-24h** | **Experiment 1**  **(Representative)** | n=6 per condition. | **(1) 37˚C-24h**  9.426±0.3929  **(2) 28˚C-24h**  4.576±0.5159 | Unpaired student’s  t-test | 37˚C-24h v. 28˚C-24h  p=0.0001, *** |
| Figure 3A | THBS1 protein expression in C2C12 myotubes secreted fraction.  2 conditions:  **(1) 37-24h**  **(2) 28-24h** | Experiment 1 | n=3 per condition. | *Total band:*  **(1) 37-24h**  32330±265  **(2) 28-24h**  27156±1061  *Top band:*  **(1) 37-24h**  19390±2032  **(2) 28-24h**  16580±1584  *Middle band:*  **(1) 37-24h**  6673±1244  **(2) 28-24h**  8755±1202  *Lower band:*  **(1) 37-24h**  6266±465.6  **(2) 28-24h**  1822±409.1 | Unpaired student’s  t-test | *Total band:*  37-24h v. 28-24h  p=0.0984, n.s  *Top band:*  37-24h v. 28-24h  p=0.3367, n.s  *Middle band:*  37-24h v. 28-24h  p=0.2951, n.s  *Lower band:*  37-24h v. 28-24h  p=0.0020, ** |
| Figure 3A | THBS1 protein expression in C2C12 myotubes secreted fraction.  2 conditions:  **(1) 37-24h**  **(2) 28-24h** | Experiment 2 | n=3 per condition. | *Total band:*  **(1) 37-24h**  45607±2564  **(2) 28-24h**  37286±1217  *Top band:*  **(1) 37-24h**  22815±1630  **(2) 28-24h**  20576±2909  *Middle band:*  **(1) 37-24h**  9318±2652  **(2) 28-24h**  9620±1598  *Lower band:*  **(1) 37-24h**  13474±2253  **(2) 28-24h**  7090±971.7 | Unpaired student’s  t-test | *Total band:*  37-24h v. 28-24h  p=0.0428, *  *Top band:*  37-24h v. 28-24h  p=0.5388, n.s  *Middle band:*  37-24h v. 28-24h  p=0.9269, n.s  *Lower band:*  37-24h v. 28-24h  p=0.0599, n.s |
| Figure 3A | THBS1 protein expression in C2C12 myotubes secreted fraction.  2 conditions:  **(1) 37-24h**  **(2) 28-24h** | Experiment 3 | n=3 per condition. | *Total band:*  **(1) 37-24h**  53035±1196  **(2) 28-24h**  38595±1927  *Top band:*  **(1) 37-24h**  27065±1503  **(2) 28-24h**  20575±1356  *Middle band:*  **(1) 37-24h**  11183±2172  **(2) 28-24h**  9785±599.7  *Lower band:*  **(1) 37-24h**  14787±957.5  **(2) 28-24h**  8235±1029 | Unpaired student’s  t-test | *Total band:*  37-24h v. 28-24h  p=0.0031, **  *Top band:*  37-24h v. 28-24h  p=0.0327, *  *Middle band:*  37-24h v. 28-24h  p=0.5685, n.s  *Lower band:*  37-24h v. 28-24h  p=0.0096, ** |
| Figure 3A | THBS1 protein expression in C2C12 myotubes secreted fraction.  2 conditions:  **(1) 37-24h**  **(2) 28-24h** | **Experiment 4**  **(Representative)** | n=6 per condition. | *Total band:*  **(1) 37-24h**  29434±2189  **(2) 28-24h**  23708±1404  *Top band:*  **(1) 37-24h**  15380±1443  **(2) 28-24h**  14285±1455  *Middle band:*  **(1) 37-24h**  3199±489.2  **(2) 28-24h**  4063±602.4  *Lower band:*  **(1) 37-24h**  10584±1106  **(2) 28-24h**  5362±711.8 | Unpaired student’s  t-test | *Total band:*  37-24h v. 28-24h  p=0.0523, n.s  *Top band:*  37-24h v. 28-24h  p=0.6047, n.s  *Middle band:*  37-24h v. 28-24h  p=0.2916, n.s  *Lower band:*  37-24h v. 28-24h  p=0.0019, ** |
| Figure 3C | THBS1 protein expression in C2C12 myotubes cellular fraction.  2 conditions:  **(1) 37-24h**  **(2) 28-24h** | **Experiment 1**  **(Representative)** | n=6 per condition. | **(1) 37-24h**  0.1808±0.01938  **(2) 28-24h**  0.4031±0.02941 | Unpaired student’s  t-test | 37-24h v. 28-24h  p<0.0001, **** |
| Figure 4A | THBS1 protein expression in C2C12 myotubes cellular fraction.  4 conditions:  **(1) 37-6h**  **(2) 28-6h**  **(3) 37-24h**  **(4) 28-24h** | **Experiment 1**  **(Representative)** | n=6 per condition. | **(1) 37-6h**  0.343±0.048  **(2) 28-6h**  0.530±0.074  **(3) 37-24h**  0.378±0.028  **(4) 28-24h**  0.677±0.040 | Two-way ANOVA  Tukey post hoc | 37-6h v. 28-24h  p=0.0007, ***  37-24h v. 28-24h  p=0.0023, ** |
| Figure 4A | THBS1 protein expression in C2C12 myotubes cellular fraction.  4 conditions:  **(1) 37-6h**  **(2) 28-6h**  **(3) 37-24h**  **(4) 28-24h** | Experiment 2 | n=6 per condition. | **(1) 37-6h**  0.306±0.015  **(2) 28-6h**  0.426±0.022  **(3) 37-24h**  0.281±0.022  **(4) 28-24h**  0.637±0.042 | Two-way ANOVA  Tukey post hoc | 37-6h v. 28-6h  p=0.0249, *  37-6h v. 28-24h  p<0.0001, ****  37-24h v. 28-6h  p=0.0059, **  37-24h v. 28-24h  p<0.0001, **** |
| Figure 4A | THBS1 protein expression in C2C12 myotubes cellular fraction.  4 conditions:  **(1) 37-6h**  **(2) 28-6h**  **(3) 37-24h**  **(4) 28-24h** | Experiment 3 | n=6 per condition. | **(1) 37-6h**  0.393±0.042  **(2) 28-6h**  0.557±0.072  **(3) 37-24h**  0.374±0.034  **(4) 28-24h**  0.747±0.049 | Two-way ANOVA  Tukey post hoc | 37-6h v. 28-24h  p=0.0005, ***  37-24h v. 28-24h  p=0.0003, *** |
| Figure 4B | THBS1 mRNA expression in C2C12 myotubes cellular fraction.  4 conditions:  **(1) 37-6h**  **(2) 28-6h**  **(3) 37-24h**  **(4) 28-24h** | Experiment 1 | n=6 per condition. | **(1)37-6h**  1.550±0.187  **(2)28-6h**  **1.212**±0.090  **(3)37-24h**  1.047±0.119  **(4)28-24h**  1.797±0.278 | Two-way ANOVA  Tukey post hoc | 37-24h v. 28-24h  p=0.0412, * |
| Figure 4B | THBS1 mRNA expression in C2C12 myotubes cellular fraction.  4 conditions:  **(1) 37-6h**  **(2) 28-6h**  **(3) 37-24h**  **(4) 28-24h** | **Experiment 2**  **(Representative)** | n=6 per condition. | **(1) 37-6h**  1.187±0.079  **(2) 28-6h**  1.117±0.046  **(3) 37-24h**  0.955±0.108  **(4) 28-24h**  2.012±0.145 | Two-way ANOVA  Tukey post hoc | 37-6h v. 28-24h  p<0.0001, ****  28-6h v. 28-24h  p<0.0001, ****  37-24h v. 28-24h  p<0.0001, **** |
| Figure 4B | THBS1 mRNA expression in C2C12 myotubes cellular fraction.  4 conditions:  **(1) 37-6h**  **(2) 28-6h**  **(3) 37-24h**  **(4) 28-24h** | Experiment 3 | n=6 per condition. | **(1) 37-6h**  1.058±0.055  **(2) 28-6h**  0.893±0.023  **(3) 37-24h**  1.230±0.065  **(4) 28-24h**  1.577±0.039 | Two-way ANOVA  Tukey post hoc | 37-6h v. 28-24h  p<0.0001, ****  37-24h v. 28-6h  p=0.0004, ***  28-6h v. 28-24h  p<0.0001, ****  37-24h v. 28-24h  p=0.0003, *** |
| Figure 4C | THBS1 protein expression in C2C12 myotubes cellular fraction.  4 conditions:  **(1) Control (37)**  **(2) Cooling (28)**  **(3) Re-warming**  **(4) Re-cooling** | **Experiment 1**  **(Representative)** | n=6 per condition. | **(1) Control (37)**  0.4007±0.03862  **(2) Cooling (28)**  0.5369±0.01745  **(3) Re-warming**  0.3408±0.03403  **(4) Re-cooling**  0.4880±0.02047 | Ordinary one-way ANOVA  Tukey’s multiple comparisons test | Control v. Cooling  p=0.0167, *  Cooling v. Re-warming  p=0.0006, ***  Re-warming v. Re-cooling  p=0.0092, ** |
| Figure 4C | THBS1 protein expression in C2C12 myotubes cellular fraction.  4 conditions:  **(1) Control (37)**  **(2) Cooling (28)**  **(3) Re-warming**  **(4) Re-cooling** | Experiment 2 | n=6 per condition. | **(1) Control (37)**  0.4357±0.03077  **(2) Cooling (28)**  0.6636±0.04257  **(3) Re-warming**  0.4223±0.04064  **(4) Re-cooling**  0.5346±0.05760 | Ordinary one-way ANOVA  Tukey’s multiple comparisons test | Control v. Cooling  p=0.0077, **  Cooling v. Re-warming  p=0.0047, ** |
| Figure 6AB | mSMECs migration, number of cells per field of view, in response to stimulation with C2C12-conditioned media.  4 conditions:  **(1) Non-stim**  **(2) Stim**  **(3) 37**  **(4) 28** | Experiment 1 | n=6 per condition. | **(1) Non-stim**  80.07±5.997  **(2) Stim**  154.7±5.366  **(3) 37**  32.47±2.054  **(4) 28**  43.63±2.732 | Ordinary one-way ANOVA  Tukey’s multiple comparisons test | Non-stim v. Stim  p<0.0001, ****  37 v. 28  p=0.3066, n.s |
| Figure 6AB | mSMECs migration, number of cells per field of view, in response to stimulation with C2C12-conditioned media.  4 conditions:  **(1) Non-stim**  **(2) Stim**  **(3) 37**  **(4) 28** | Experiment 2 | n=6 per condition. | **(1) Non-stim**  39.46±3.812  **(2) Stim**  75.72±2.422  **(3) 37**  36.03±1.346  **(4) 28**  43.40±5.948 | Ordinary one-way ANOVA  Tukey’s multiple comparisons test | Non-stim v. Stim  p<0.0001, ****  37 v. 28  p=0.5289, n.s |
| Figure 6AB | mSMECs migration, number of cells per field of view, in response to stimulation with C2C12-conditioned media.  4 conditions:  **(1) Non-stim**  **(2) Stim**  **(3) 37**  **(4) 28** | **Experiment 3**  **(Representative)** | n=6 per condition. | **(1) Non-stim**  26.63±3.662  **(2) Stim**  57.54±2.891  **(3) 37**  29.78±3.318  **(4) 28**  24.50±6.016 | Ordinary one-way ANOVA  Tukey’s multiple comparisons test | Non-stim v. Stim  p=0.0002, ***  37 v. 28  p=0.8055, n.s |
| Figure 6AB | mSMECs migration, number of cells per field of view, in response to stimulation with C2C12-conditioned media.  4 conditions:  **(1) Non-stim**  **(2) Stim**  **(3) 37**  **(4) 28** | Experiment 4 | n=6 per condition. | **(1) Non-stim**  89.45±7.740  **(2) Stim**  142.9±3.685  **(3) 37**  105.0±6.118  **(4) 28**  92.17±5.038 | Ordinary one-way ANOVA  Tukey’s multiple comparisons test | Non-stim v. Stim  p<0.0001, ****  37 v. 28  p=0.4256, n.s |
| Figure 6AB | mSMECs migration, number of cells per field of view, in response to stimulation with C2C12-conditioned media.  4 conditions:  **(1) Non-stim**  **(2) Stim**  **(3) 37**  **(4) 28** | Experiment 5 | n=6 per condition. | **(1) Non-stim**  53.13±5.744  **(2) Stim**  97.04±3.038  **(3) 37**  31.70±2.481  **(4) 28**  36.82±5.096 | Ordinary one-way ANOVA  Tukey’s multiple comparisons test | Non-stim v. Stim  p<0.0001, ****  37 v. 28  p=0.8348, n.s |
| Figure 6AB | mSMECs migration, number of cells per field of view, in response to stimulation with C2C12-conditioned media.  4 conditions:  **(1) Non-stim**  **(2) Stim**  **(3) 37**  **(4) 28** | Experiment 6 | n=6 per condition. | **(1) Non-stim**  38.25±2.513  **(2) Stim**  84.75±1.751  **(3) 37**  32.56±1.221  **(4) 28**  33.50±3.096 | Ordinary one-way ANOVA  Tukey’s multiple comparisons test | Non-stim v. Stim  p<0.0001, ****  37 v. 28  p=0.9907, n.s |
| Figure 7A | RBM3 protein expression in mSMEC cellular fraction.  2 conditions:  **(1)37-24h**  **(2)28-24h** | **Experiment 1**  **(Representative)** | n=6 per condition. | **(1)37-24h**  1.061±0.04477  **(2)28-24h**  1.300±0.04630 | Unpaired student’s  t-test | 37-24h v. 28-24h  p=0.0041, ** |
| Figure 7A | RBM3 protein expression in mSMEC cellular fraction.  2 conditions:  **(1)37-24h**  **(2)28-24h** | Experiment 2 | n=6 per condition. | **(1)37-24h**  1.275±0.05246  **(2)28-24h**  1.454±0.04762 | Unpaired student’s  t-test | 37-24h v. 28-24h  p=0.0303, * |
| Figure 7A | RBM3 protein expression in mSMEC cellular fraction.  2 conditions:  **(1)37-24h**  **(2)28-24h** | Experiment 3 | n=6 per condition. | **(1)37-24h**  1.051±0.06923  **(2)28-24h**  1.238±0.05614 | Unpaired student’s  t-test | 37-24h v. 28-24h  p=0.0624, n.s |
| Figure 7B | mSMEC proliferation in either control (37) or cold (28) conditions.  3 timepoints analyzed: Day0, Day1, Day2.  5 conditions:  **(1) Day0**  **(2) Day1-37**  **(3) Day1-28**  **(4) Day2-37**  **(5) Day2-28** | **Experiment 1**  **(Representative)** | n=8 per conditions (1), (2), (3), (5).  n=5 per condition (4). | **(1) Day0**  16538.375±716.125  **(2) Day1-37**  36672.620±3488.868  **(3) Day1-28**  23651.394±2769.046  **(4) Day2-37**  111832.120±3995.202  **(5) Day2-28**  24403.701±1582.401 | Two-way ANOVA  Tukey post hoc | Day0 v. Day1-37  p<0.0001, ****  Day0 v. Day2-37  p<0.0001, ****  Day1-37 v. Day1-28  p=0.0032, **  Day2-37 v. Day2-28  p<0.0001, **** |
| Figure 7B | mSMEC proliferation in either control (37) or cold (28) conditions.  3 timepoints analyzed: Day0, Day1, Day2.  5 conditions:  **(1) Day0**  **(2) Day1-37**  **(3) Day1-28**  **(4) Day2-37**  **(5) Day2-28** | Experiment 2 | n=8 per condition. | **(1) Day0**  16683.875±1829.976  **(2) Day1-37**  54183.824±5434.26  **(3) Day1-28**  30995.811±1006.853  **(4) Day2-37**  108599.115±8182.196  **(5) Day2-28**  22594.660±1706.450 | Two-way ANOVA  Tukey post hoc3 | Day0 v. Day1-37  p<0.0001, ****  Day0 v. Day2-37  p<0.0001, ****  Day1-37 v. Day1-28  p=0.0046, **  Day2-37 v. Day2-28  p<0.0001, **** |
| Figure 7B | mSMEC proliferation in either control (37) or cold (28) conditions.  3 timepoints analyzed: Day0, Day1, Day2.  5 conditions:  **(1) Day0**  **(2) Day1-37**  **(3) Day1-28**  **(4) Day2-37**  **(5) Day2-28** | Experiment 3 | n=8 per conditions (1), (2), (3), (5).  n=4 per condition (4). | **(1) Day0**  16302.000±1048.748  **(2) Day1-37**  55455.363±4525.666  **(3) Day1-28**  31682.126±2288.485  **(4) Day2-37**  83267.125±14925.931  **(5) Day2-28**  37742.983±4975.329 | Two-way ANOVA  Tukey post hoc | Day0 v. Day1-37  p<0.0001, ****  Day0 v. Day2-37  p<0.0001, ***  Day1-37 v. Day2-28  p=0.0046, **  Day2-37 v. Day2-28  p<0.0001, **** |
| Figure 8A | mSMEC non-stimulated (Non-stim) and stimulated (Stim) rates of migration, number of cells per field of view, following preconditioning in either control (37) or cold (28) conditions.  4 conditions:  **(1) Non-stim-37**  **(2) Non-stim-28**  **(3) Stim-37**  **(4) Stim-28** | Experiment 1 | n=6 per condition. | **(1) Non-stim-37**  53.444±3.424  **(2) Non-stim-28**  37.875±2.916  **(3) Stim-37**  93.875±3.108  **(4) Stim-28**  77.736±2.477 | Two-way ANOVA  Tukey post hoc | Non-stim 37 v. Stim 37  p<0.0001, ****  Non-stim 28 v. Stim 28  p<0.0001, ****  Non-stim 37 v. Non-stim 28  p=0.0076, **  Stim 37 v. Stim 28  p=0.0057, ** |
| Figure 8A | mSMEC non-stimulated (Non-stim) and stimulated (Stim) rates of migration, number of cells per field of view, following preconditioning in either control (37) or cold (28) conditions.  4 conditions:  **(1) Non-stim-37**  **(2) Non-stim-28**  **(3) Stim-37**  **(4) Stim-28** | Experiment 2 | n=6 per condition. | **(1) Non-stim-37**  29.111±2.265  **(2) Non-stim-28**  17.250±1.982  **(3) Stim-37**  86.042±2.740  **(4) Stim-28**  76.125±3.418 | Two-way ANOVA  Tukey post hoc | Non-stim 37 v. Stim 37  p<0.0001, ****  Non-stim 28 v. Stim 28  p<0.0001, ****  Non-stim 37 v. Non-stim 28  p=0.0236, *  Stim 37 v. Stim 28  p=0.0691, n.s |
| Figure 8A | mSMEC non-stimulated (Non-stim) and stimulated (Stim) rates of migration, number of cells per field of view, following preconditioning in either control (37) or cold (28) conditions.  4 conditions:  **(1)Non-stim-37**  **(2)Non-stim-28**  **(3)Stim-37**  **(4)Stim-28** | **Experiment 3**  **(Representative)** | n=6 per condition. | **(1)Non-stim-37**  21.917±1.616  **(2)Non-stim-28**  15.083±1.621  **(3)Stim-37**  56.792±2.737  **(4)Stim-28**  52.958±1.787 | Two-way ANOVA  Tukey post hoc | Non-stim 37 v. Stim 37  p<0.0001, ****  Non-stim 28 v. Stim 28  p<0.0001, ****  Non-stim 37 v. Non-stim 28  p=0.1050, n.s  Stim 37 v. Stim 28  p=0.5383, n.s |

| Figure 8A | mSMEC non-stimulated (Non-stim) and stimulated (Stim) rates of migration, number of cells per field of view, following preconditioning in either control (37) or cold (28) conditions.  4 conditions:  **(1)Non-stim-37**  **(2)Non-stim-28**  **(3)Stim-37**  **(4)Stim-28** | Experiment 4 | n=6 per condition. | **(1)Non-stim-37**  37.125±3.460  **(2)Non-stim-28**  32.861±1.308  **(3)Stim-37**  73.167±5.108  **(4)Stim-28**  93.681±2.617 | Two-way ANOVA  Tukey post hoc | Non-stim 37 v. Stim 37  p<0.0001, ****  Non-stim 28 v. Stim 28  p<0.0001, ****  Non-stim 37 v. Non-stim 28  p=0.8135, n.s  Stim 37 v. Stim 28  p=0.0021, ** |
| --- | --- | --- | --- | --- | --- | --- |
| Figure 8A | mSMEC non-stimulated (Non-stim) and stimulated (Stim) rates of migration, number of cells per field of view, following preconditioning in either control (37) or cold (28) conditions.  4 conditions:  **(1)Non-stim-37**  **(2)Non-stim-28**  **(3)Stim-37**  **(4)Stim-28** | Experiment 5 | n=6 per condition. | **(1)Non-stim-37**  36.722±2.668  **(2)Non-stim-28**  34.639±2.640  **(3)Stim-37**  74.625±2.303  **(4)Stim-28**  79.417±3.537 | Two-way ANOVA  Tukey post hoc | Non-stim 37 v. Stim 37  p<0.0001, ****  Non-stim 28 v. Stim 28  p<0.0001, ****  Non-stim 37 v. Non-stim 28  p=0.9529, n.s  Stim 37 v. Stim 28  p=0.6340, n.s |
| Figure 8B | mSMEC fold increase in stimulated migration relative to unstimulated conditions.  2 conditions:  **(1)** 37 Stim / 37 Non-stim  **(2)** 28 Stim / 28 Non-stim | Experiment 1 | n=6 per condition. | **(1) 37 Stim / 37 Non-stim**  1.756±0.05816  **(2) 28 Stim / 28 Non-stim**  2.052±0.06539 | Unpaired student’s  t-test | 37 v. 28  p=0.0070, ** |
| Figure 8B | mSMEC fold increase in stimulated migration relative to unstimulated conditions.  2 conditions:  **(1)** 37 Stim / 37 Non-stim  **(2)** 28 Stim / 28 Non-stim | Experiment 2 | n=6 per condition. | **(1) 37 Stim / 37 Non-stim**  2.956±0.09414  **(2) 28 Stim / 28 Non-stim**  4.413±0.1981 | Unpaired student’s  t-test | 37 v. 28  p<0.0001, **** |
| Figure 8B | mSMEC fold increase in stimulated migration relative to unstimulated conditions.  2 conditions:  **(1)** 37 Stim / 37 Non-stim  **(2)** 28 Stim / 28 Non-stim | **Experiment 3**  **(Representative)** | n=6 per condition. | **(1) 37 Stim / 37 Non-stim**  2.591±0.1249  **(2) 28 Stim / 28 Non-stim**  3.511±0.1185 | Unpaired student’s  t-test | 37 v. 28  p=0.0003, *** |
| Figure 8B | mSMEC fold increase in stimulated migration relative to unstimulated conditions.  2 conditions:  **(1)** 37 Stim / 37 Non-stim  **(2)** 28 Stim / 28 Non-stim | Experiment 4 | n=6 per condition. | **(1) 37 Stim / 37 Non-stim**  1.971±0.1376  **(2) 28 Stim / 28 Non-stim**  2.851±0.07964 | Unpaired student’s  t-test | 37 v. 28  p=0.0002, *** |
| Figure 8B | mSMEC fold increase in stimulated migration relative to unstimulated conditions.  2 conditions:  **(1)** 37 Stim / 37 Non-stim  **(2)** 28 Stim / 28 Non-stim | Experiment 5 | n=6 per condition. | **(1) 37 Stim / 37 Non-stim**  2.032±0.06271  **(2) 28 Stim / 28 Non-stim**  2.293±0.07964 | Unpaired student’s  t-test | 37 v. 28  p=0.0548, n.s |
| Figure 8C | mSMEC proliferation in response to preconditioning in either control (37) or cold (28) conditions.  6 conditions:  **(1) T0-37**  **(2) T0-28**  **(3) T24-37**  **(4) T24-28**  **(5) T48-37**  **(6) T48-28** | Experiment 1 | n=6-7 per condition. | **(1) T0-37**  8169.143±650.337  **(2) T0-28**  12087.571±747.131  **(3) T24-37**  18107.674±1598.931  **(4) T24-28**  19505.903±1242.522  **(5) T48-37**  47634.640±4958.407  **(6) T48-28**  57621.211±2630.831 | Two-way ANOVA  Tukey post hoc | T0-37 v. T24-37  p=0.0727, n.s  T0-37 v. T48-37  p<0.0001, ****  T0-28 v. T24-28  p=0.2982, n.s  T0-28 v. T48-28  p<0.0001, **** |
| Figure 8C | mSMEC proliferation in response to preconditioning in either control (37) or cold (28) conditions.  6 conditions:  **(1) T0-37**  **(2) T0-28**  **(3) T24-37**  **(4) T24-28**  **(5) T48-37**  **(6) T48-28** | Experiment 2 | n=6-7 per condition. | **(1) T0-37**  11809.533±1226.298  **(2) T0-28**  11684.476±704.436  **(3) T24-37**  20887.000±1884.091  **(4) T24-28**  17522.500±1067.984  **(5) T48-37**  38021.053±5268.859  **(6) T48-28**  43451.337±4124.183 | Two-way ANOVA  Tukey post hoc | T0-37 v. T24-37  p=0.2780, n.s  T0-37 v. T48-37  p<0.0001, ****  T0-28 v. T24-28  p=0.7613, n.s  T0-28 v. T48-28  p<0.0001, **** |
| Figure 8C | mSMEC proliferation in response to preconditioning in either control (37) or cold (28) conditions.  6 conditions:  **(1) T0-37**  **(2) T0-28**  **(3) T24-37**  **(4) T24-28**  **(5) T48-37**  **(6) T48-28** | Experiment 3 | n=6-7 per condition. | **(1) T0-37**  12403.500±1088.278  **(2) T0-28**  10396.833±825.534  **(3) T24-37**  23810.309±3014.776  **(4) T24-28**  18709.522±1944.749  **(5) T48-37**  47260.700±8192.986  **(6) T48-28**  34529.847±4597.213 | Two-way ANOVA  Tukey post hoc | T0-37 v. T24-37  p=0.3452, n.s  T0-37 v. T48-37  p<0.0001, ****  T0-28 v. T24-28  p=0.6766, n.s  T0-28 v. T48-28  p<0.0029, ** |
| Figure 9BD | Basal (230kda, 210kda, 150kda) and phosphorylated (Y1175) protein expression of VEGFR2 in mSMEC.  4 conditions:  **(1)-VEGF-37**  **(2)-VEGF-28**  **(3)+VEGF-37**  **(4)+VEGF-28** | Experiment 1 | n=3 per condition. | *230kda*  **(1)-VEGF-37**  0.541±0.135  **(2)-VEGF-28**  0.640±0.149  **(3)+VEGF-37**  0.323±0.071  **(4)+VEGF-28**  0.513±0.062  *210kda*  **(1)-VEGF-37**  0.217±0.056  **(2)-VEGF-28**  0.475±0.060  **(3)+VEGF-37**  0.313±0.047  **(4)+VEGF-28**  0.545±0.040  *150kda*  **(1)-VEGF-37**  0.121±0.034  **(2)-VEGF-28**  0.570±0.128  **(3)+VEGF-37**  0.209±0.062  **(4)+VEGF-28**  0.949±0.195  *Y1175*  **(1)-VEGF-37**  0.139±0.017  **(2)-VEGF-28**  0.093±0.031  **(3)+VEGF-37**  0.563±0.096  **(4)+VEGF-28**  0.612±0.048  *Y1175/230kda*  **(1)-VEGF-37**  0.279±0.047  **(2)-VEGF-28**  0.164±0.060  **(3)+VEGF-37**  1.793±0.203  **(4)+VEGF-28**  1.250±0.248 | Two-way ANOVA  Tukey post hoc | ***230kda***  *n.s*  ***210kda***  -VEGF-37 v. -VEGF-28  p=0.0303, *  +VEGF-37 v. +VEGF-28  p=0.0495,*  ***150kda***  +VEGF-37 v. +VEGF-28  p=0.0115, *  ***Y1175***  -VEGF-37 v. +VEGF-37  p=0.0031, **  -VEGF-28 v. +VEGF-28  p=0.0008, ***  ***Y1175/230kda***  -VEGF-37 v. +VEGF-37  p=0.0009, ***  -VEGF-28 v. +VEGF-28  p=0.0070, ** |
| Figure 9BD | Basal (230kda, 210kda, 150kda) and phosphorylated (Y1175) protein expression of VEGFR2 in mSMEC.  4 conditions:  **(1)-VEGF-37**  **(2)-VEGF-28**  **(3)+VEGF-37**  **(4)+VEGF-28** | **Experiment 2**  **(Representative)** | n=3 per condition. | *230kda*  **(1)-VEGF-37**  0.376±0.021  **(2)-VEGF-28**  0.700±0.021  **(3)+VEGF-37**  0.152±0.040  **(4)+VEGF-28**  0.438±0.114  *210kda*  **(1)-VEGF-37**  0.099±0.015  **(2)-VEGF-28**  0.301±0.044  **(3)+VEGF-37**  0.140±0.039  **(4)+VEGF-28**  0.435±0.071  *150kda*  **(1)-VEGF-37**  0.410±0.116  **(2)-VEGF-28**  0.794±0.183  **(3)+VEGF-37**  0.377±0.098  **(4)+VEGF-28**  1.736±0.229  *Y1175*  **(1)-VEGF-37**  0.238±0.030  **(2)-VEGF-28**  0.138±0.016  **(3)+VEGF-37**  0.398±0.032  **(4)+VEGF-28**  0.268±0.019  *Y1175/230kda*  **(1)-VEGF-37**  0.626±0.050  **(2)-VEGF-28**  0.198±0.028  **(3)+VEGF-37**  2.945±0.721  **(4)+VEGF-28**  0.684±0.147 | Two-way ANOVA  Tukey post hoc | ***230kda***  -VEGF-37 v. -VEGF-28  p=0.0258, *  +VEGF-37 v. +VEGF-28  p=0.0470, *  ***210kda***  +VEGF-37 v. +VEGF-28  p=0.0090, **  ***150kda***  -VEGF-28 v. +VEGF-28  p=0.0159, *  +VEGF-37 v. +VEGF-28  p=0.0018, **  ***Y1175***  -VEGF-37 v. +VEGF-37  p=0.0090, **  -VEGF-28 v. +VEGF-28  p=0.0272, *  +VEGF-37 v. +VEGF-28  p=0.0279, *  ***Y1175/230kda***  -VEGF-37 v. +VEGF-37  p=0.0093, **  +VEGF-37 v. +VEGF-28  p=0.0107, * |
| Figure 9BD | Basal (230kda, 210kda, 150kda) and phosphorylated (Y1175) protein expression of VEGFR2 in mSMEC.  4 conditions:  **(1)-VEGF-37**  **(2)-VEGF-28**  **(3)+VEGF-37**  **(4)+VEGF-28** | Experiment 3 | n=3 per condition. | *230kda*  **(1)-VEGF-37**  0.296±0.063  **(2)-VEGF-28**  0.537±0.066  **(3)+VEGF-37**  0.099±0.013  **(4)+VEGF-28**  0.335±0.098  *210kda*  **(1)-VEGF-37**  0.097±0.022  **(2)-VEGF-28**  0.285±0.070  **(3)+VEGF-37**  0.114±0.018  **(4)+VEGF-28**  0.322±0.070  *150kda*  **(1)-VEGF-37**  0.138±0.055  **(2)-VEGF-28**  0.422±0.119  **(3)+VEGF-37**  0.191±0.034  **(4)+VEGF-28**  0.806±0.255  *Y1175*  **(1)-VEGF-37**  0.556±0.135  **(2)-VEGF-28**  0.420±0.034  **(3)+VEGF-37**  0.981±0.207  **(4)+VEGF-28**  0.722±0.185  *Y1175/230kda*  **(1)-VEGF-37**  2.185±0.806  **(2)-VEGF-28**  0.811±0.144  **(3)+VEGF-37**  9.817±1.120  **(4)+VEGF-28**  2.370±0.634 | Two-way ANOVA  Tukey post hoc | ***230kda***  *n.s*  ***210kda***  n.s  ***150kda***  n.s  ***Y1175***  n.s  ***Y1175/230kda***  -VEGF-37 v. +VEGF-37  p=0.0005, ***  +VEGF-37 v. +VEGF-28  p=0.0006, *** |
| Figure 11B | THBS1 protein expression in ex-vivo muscle incubation assay samples exposed to control (37) or cold (28) conditions.  2 conditions:  **(1)37-24h**  **(2)28-24h** | **Experiment 1**  **(Representative)** | n=6 per condition. | **(1)37-24h**  0.5144±0.1107  **(2)28-24h**  1.032±0.1703 | Unpaired student’s  t-test | 37 v. 28  p=0.0290, * |

# Differentiated C2C12 myotubes (after 5 days of differentiation) and cultured up to 6h and 24h at 37˚C or 28˚C.

**C2C12, 37˚C, 5 days + 0h, 10x**


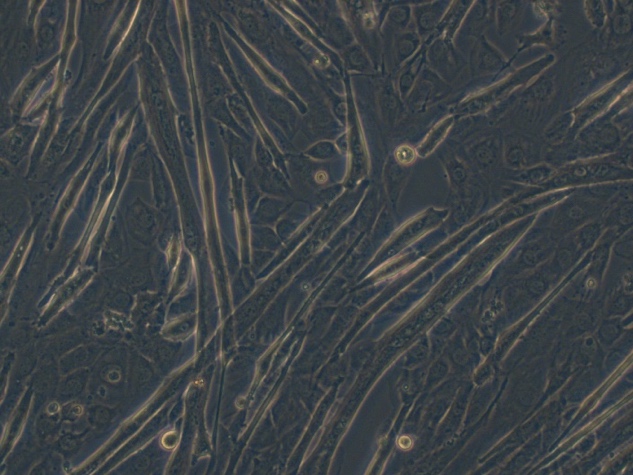

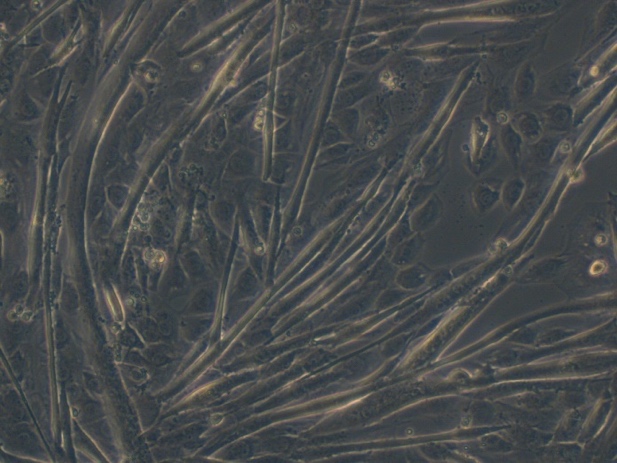


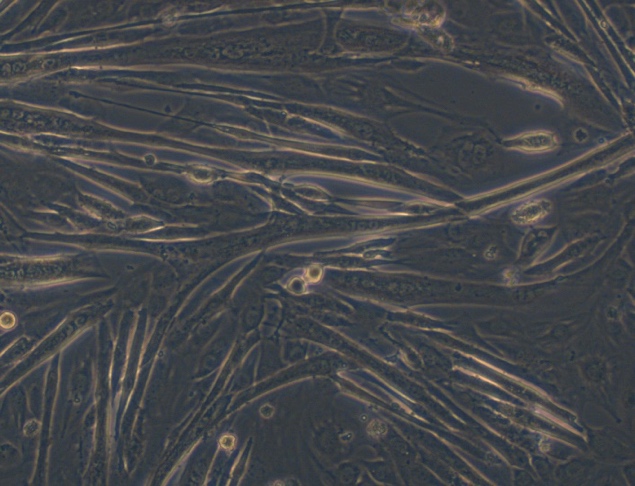

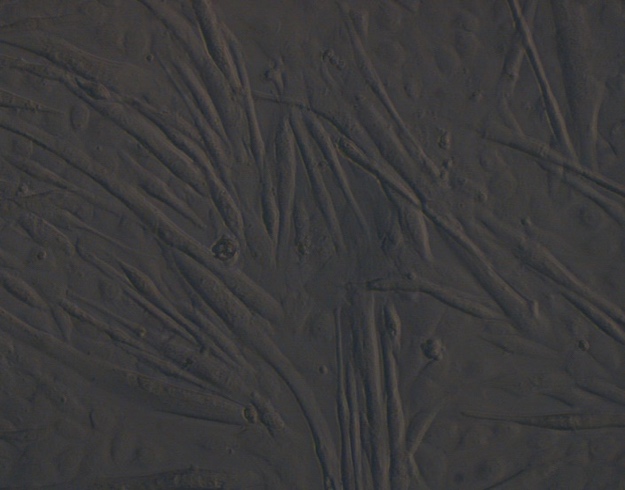


**C2C12, 37˚C, 5 days + 0h, 4x**


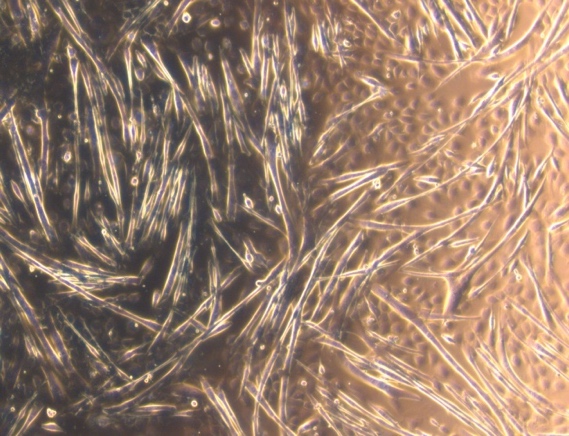

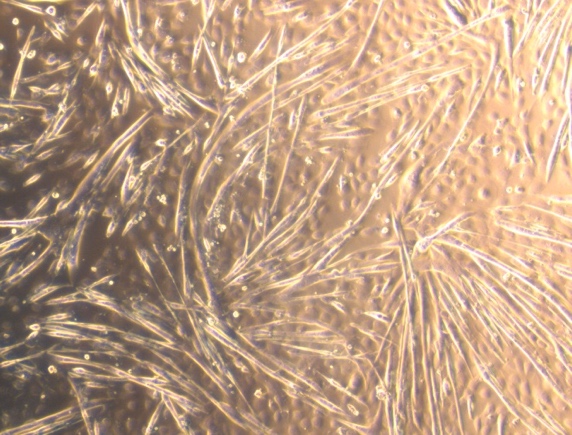

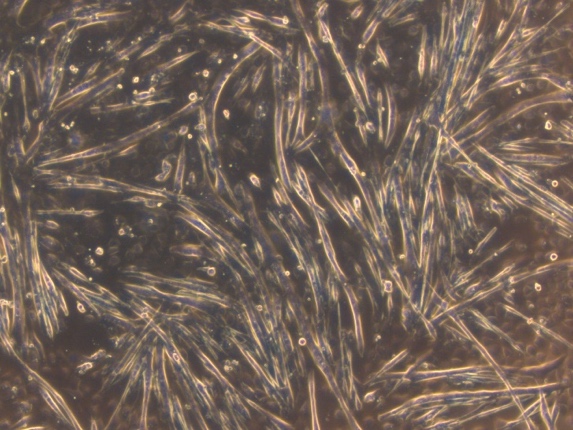

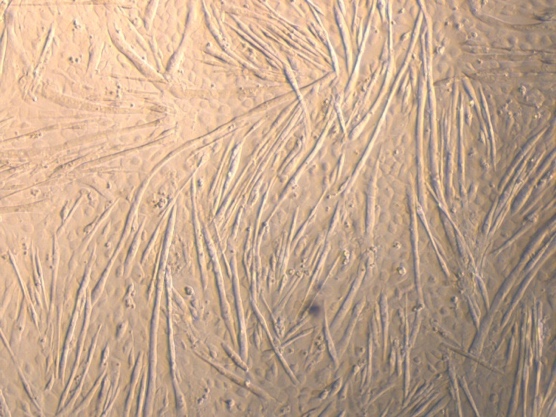


**C2C12, 28˚C, 5 days + 0h, 10x**


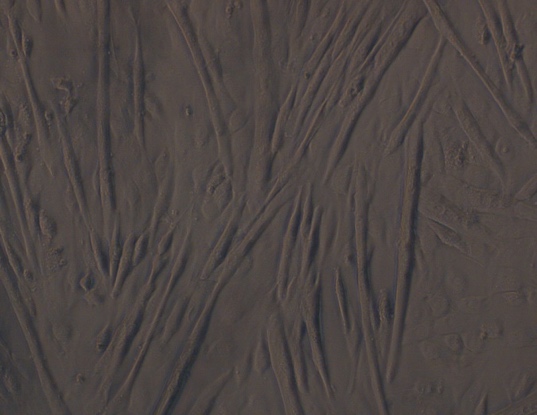

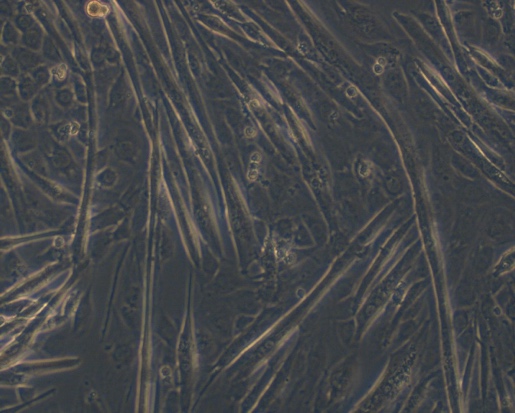


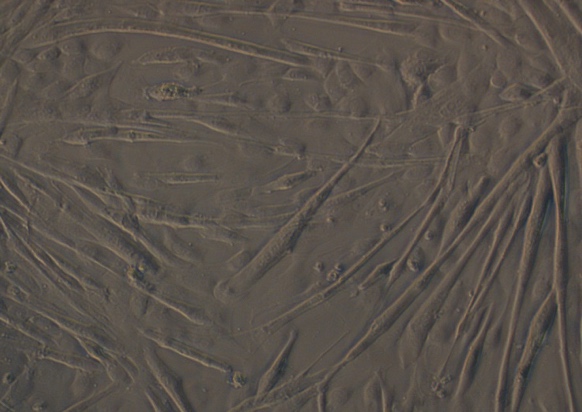

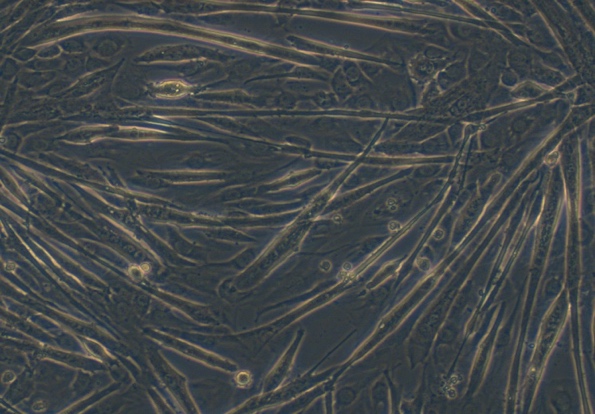


**C2C12, 28˚C, 5 days + 0h, 4x**


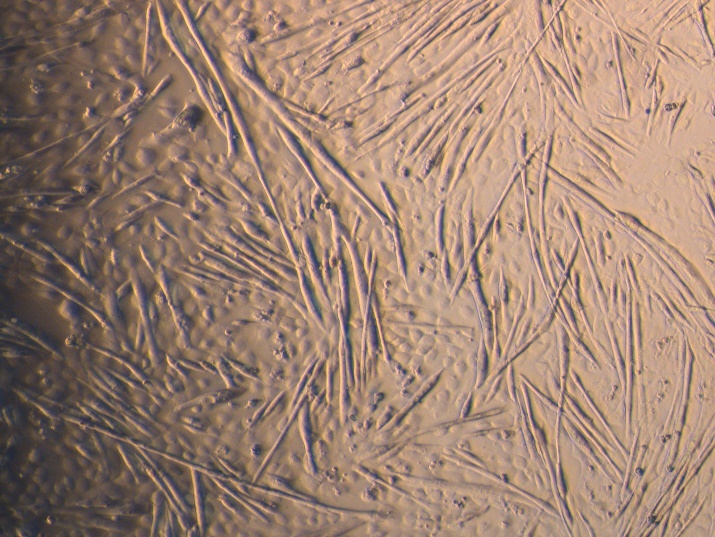

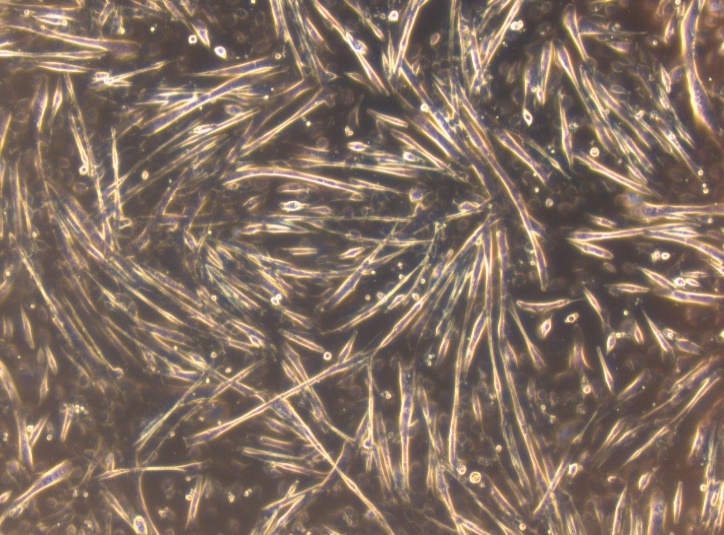


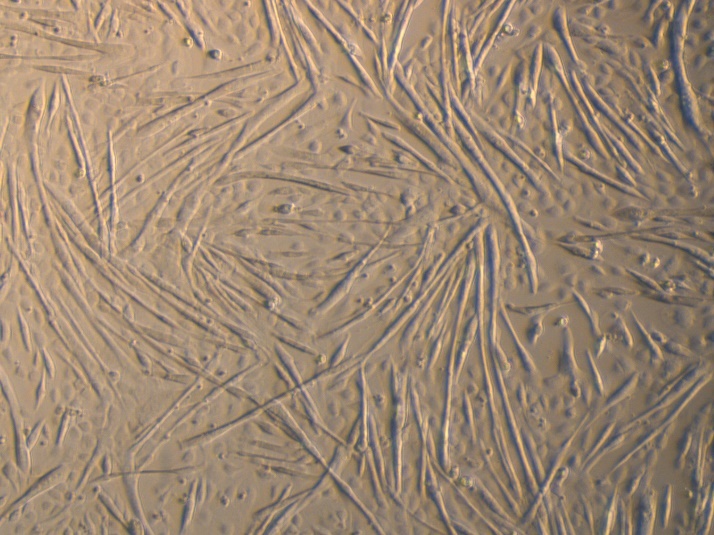

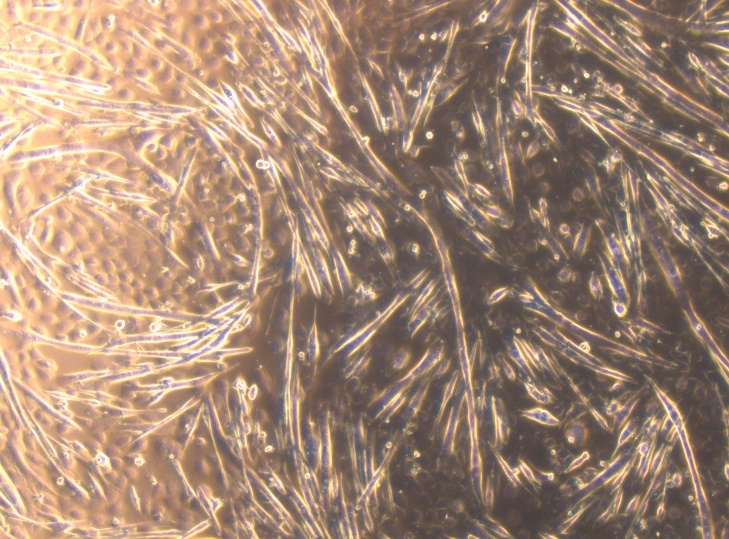


**C2C12, 37˚C, 5 days + 6h, 10x**


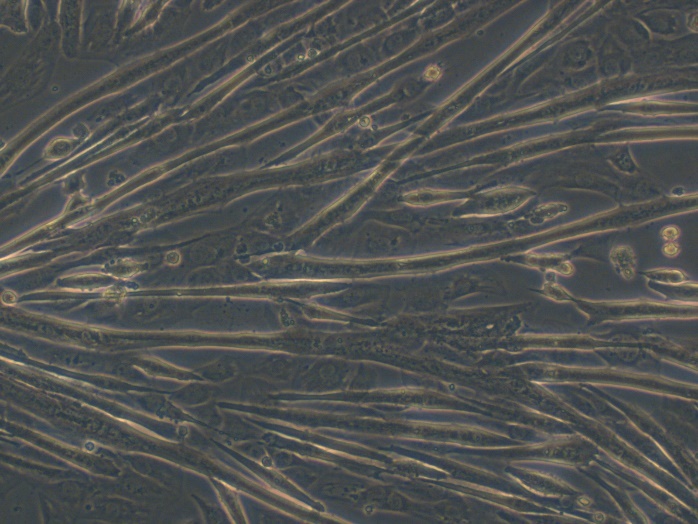

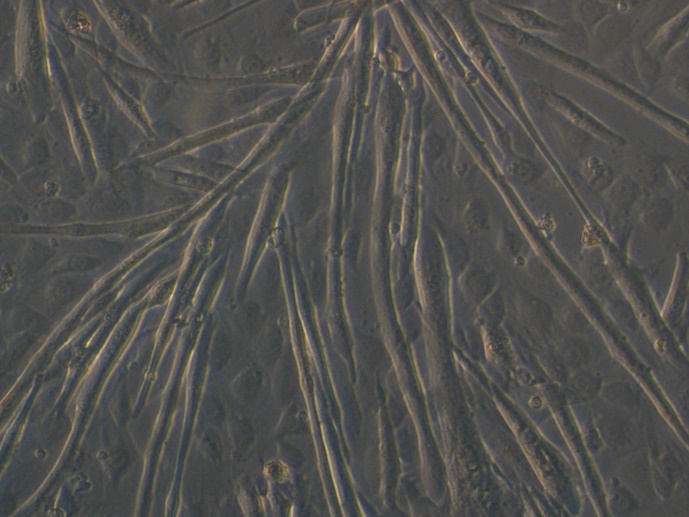


**C2C12, 37˚C, 5 days + 6h, 4x**


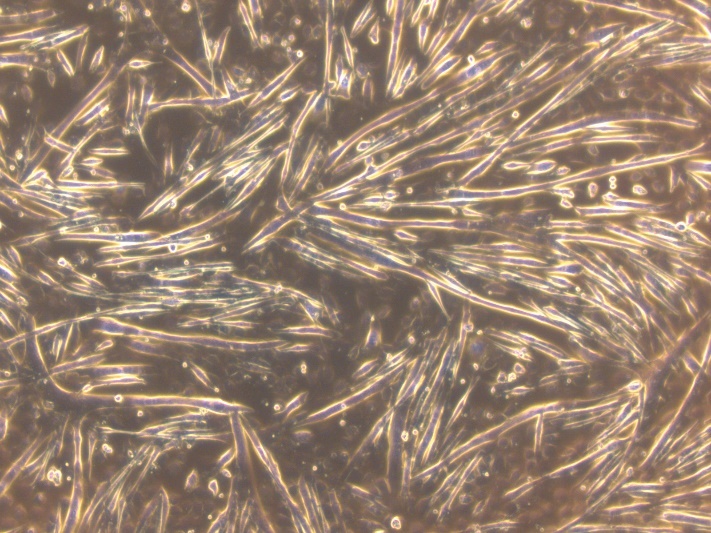

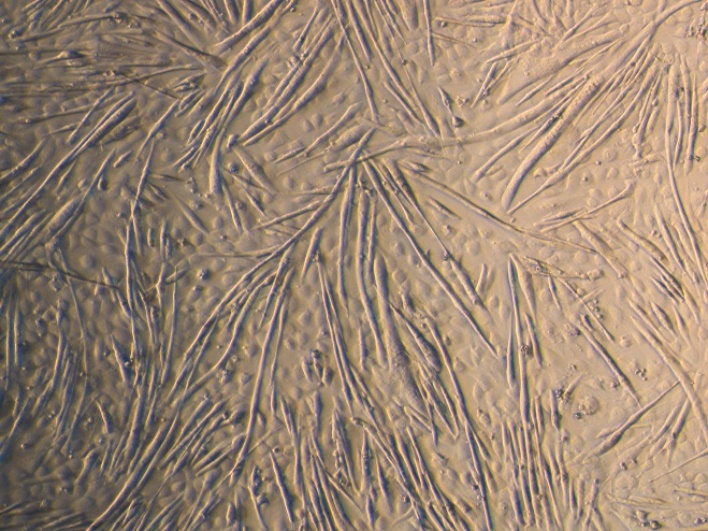


**C2C12, 28˚C, 5 days + 6h, 10x**


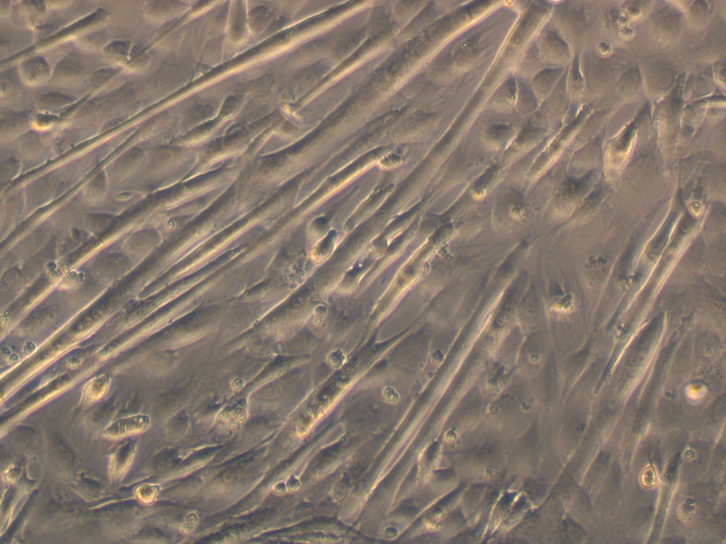

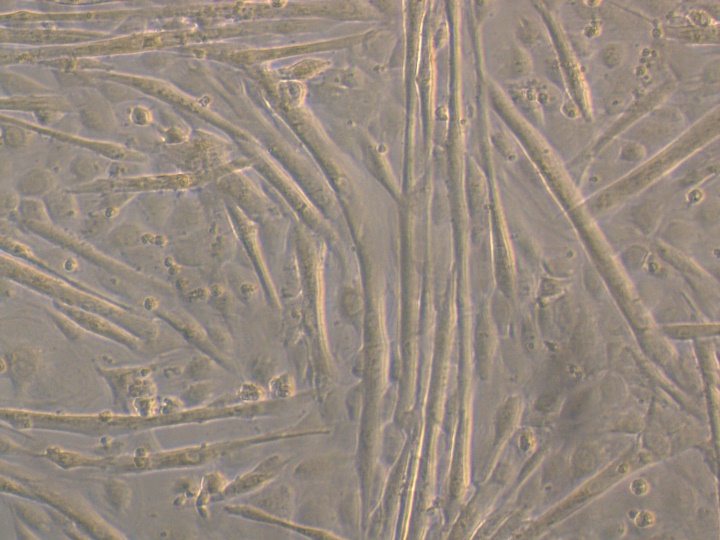


**C2C12, 28˚C, 5 days + 6h, 4x**


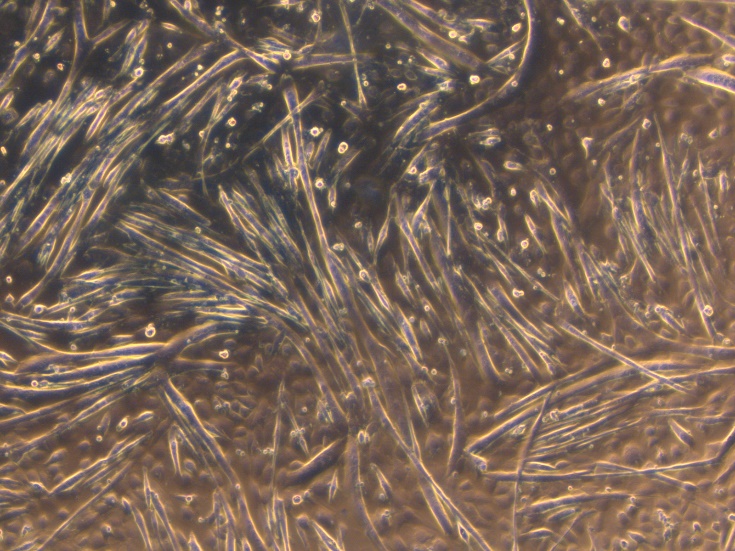

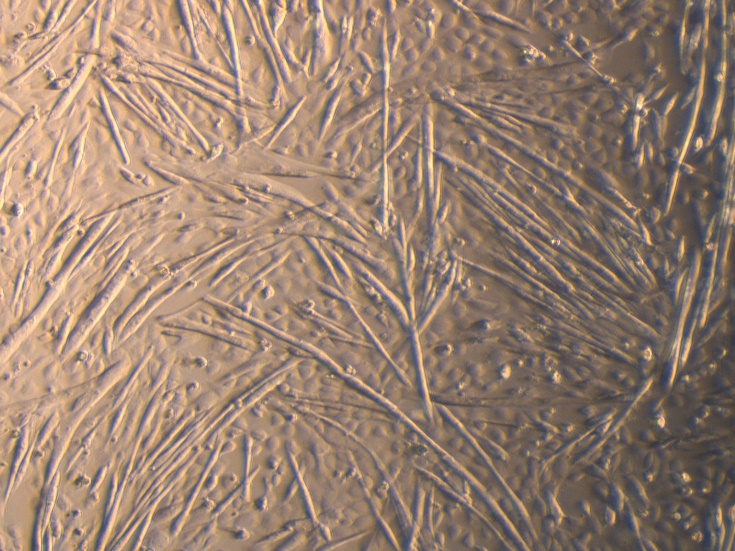


**C2C12, 37˚C, 5 days + 24h, 10x**


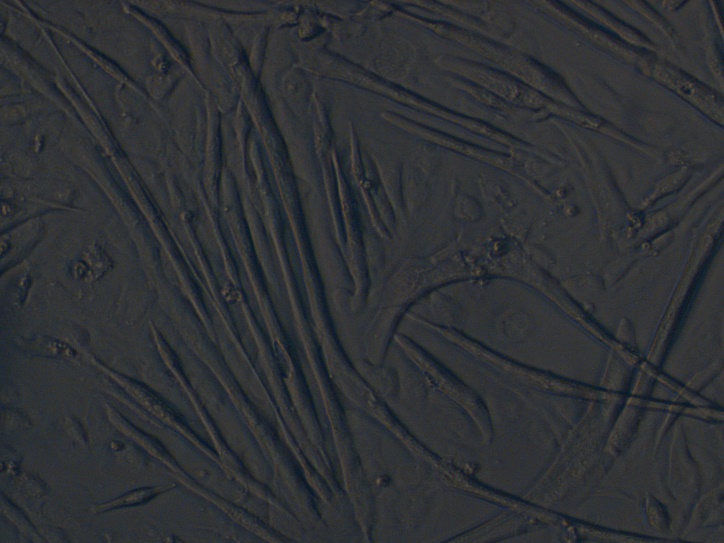

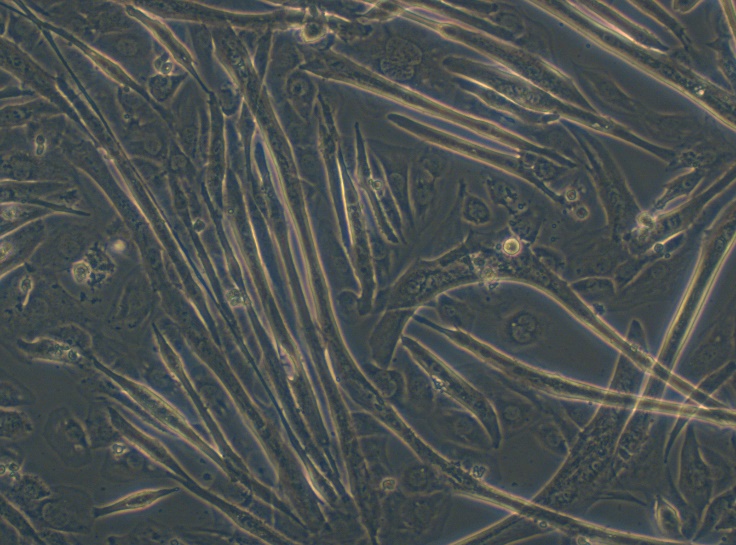


**C2C12, 37˚C, 5 days + 24h, 4x**


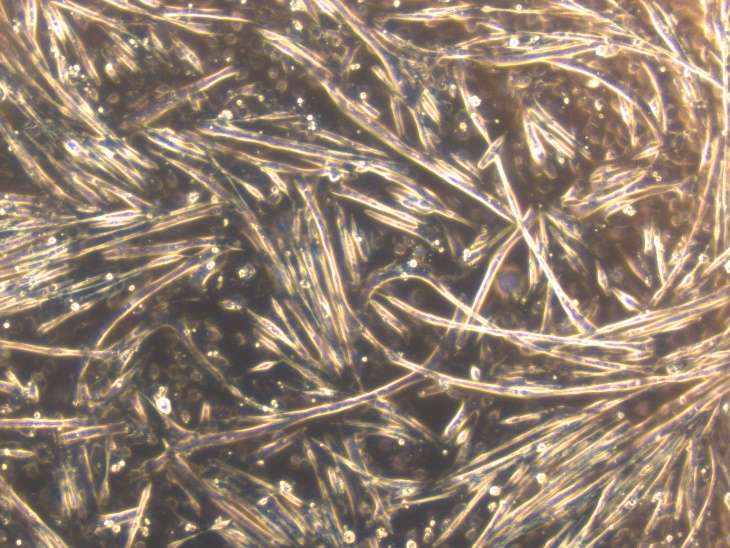

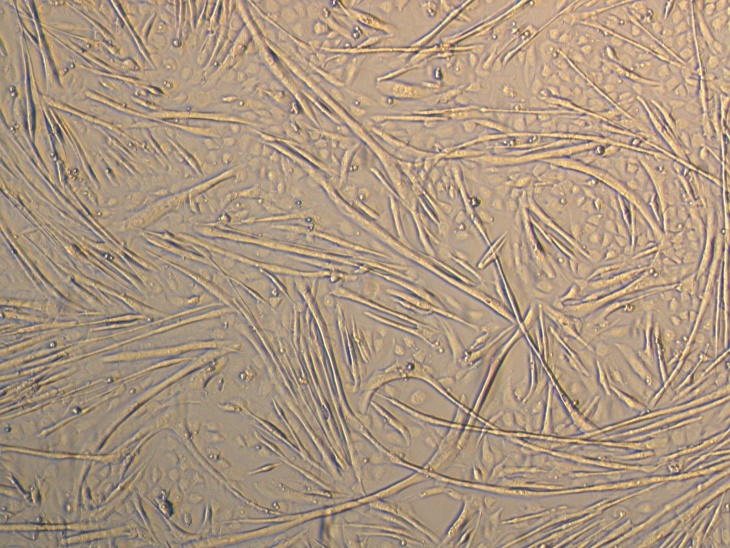


**C2C12, 28˚C, 5 days + 24h, 10x**


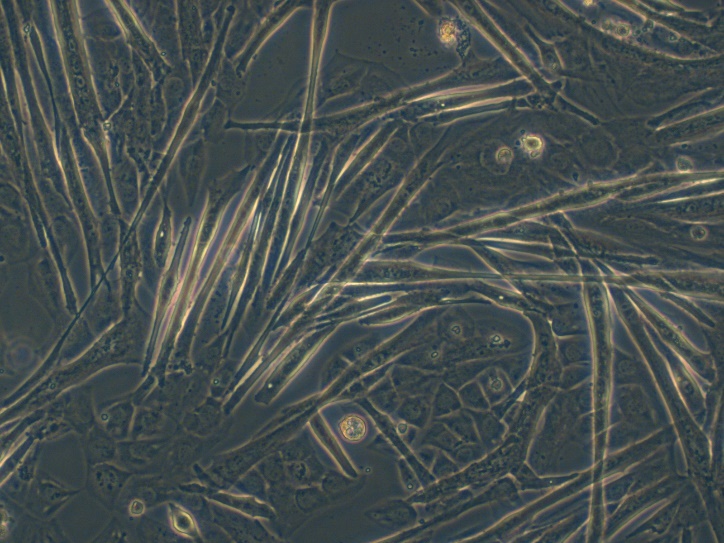

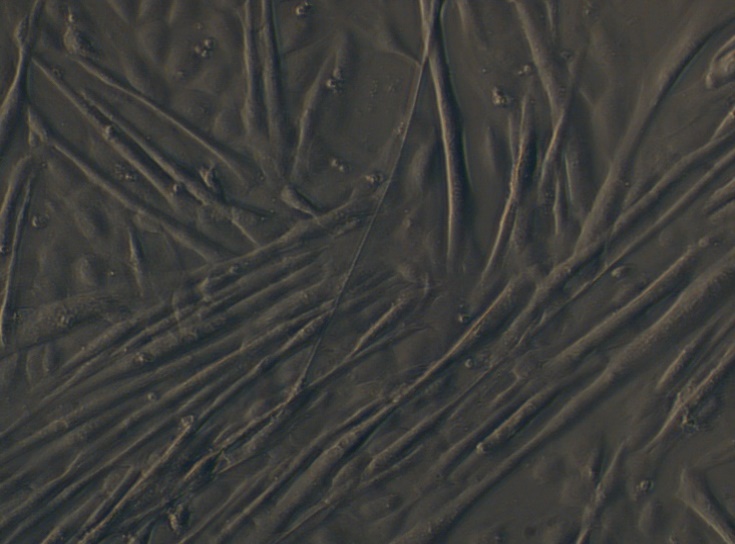


**C2C12, 28˚C, 5 days + 24h, 4x**


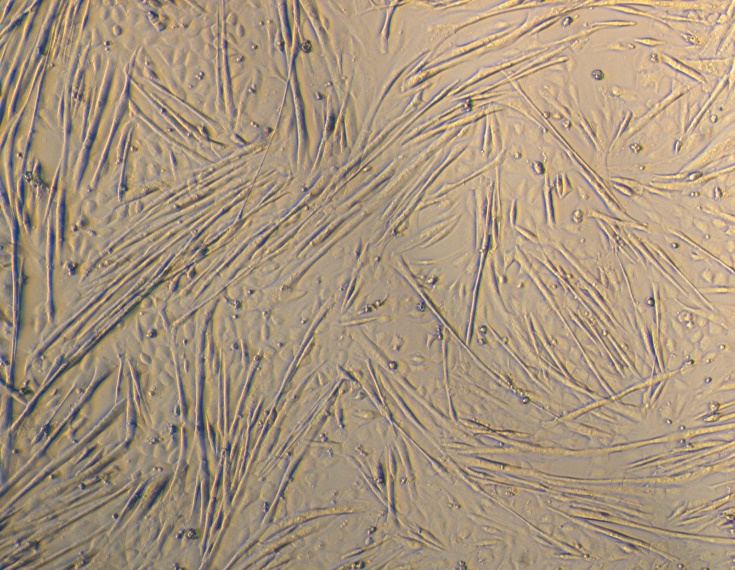

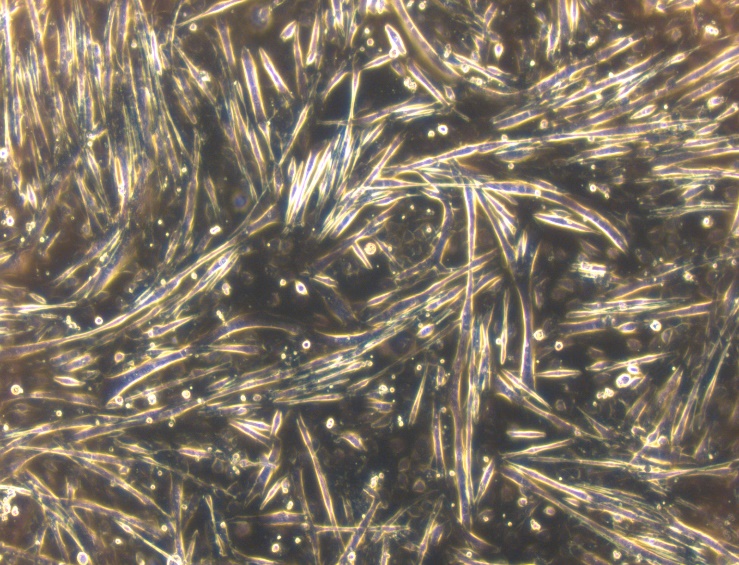


**3 Ponceau S staining images for cellular protein detection**

**FIGURE 2A, RBM3 detection in C2C12 at 6h, Experiment #1**

**
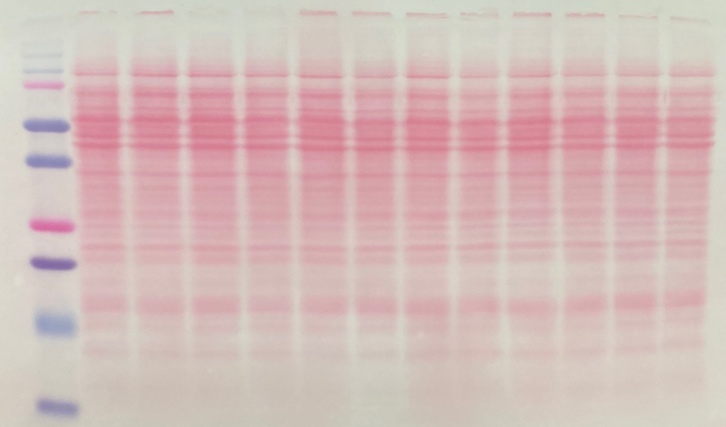
**

**FIGURE 2A, RBM3 detection in C2C12 at 6h, Experiment #2**

**
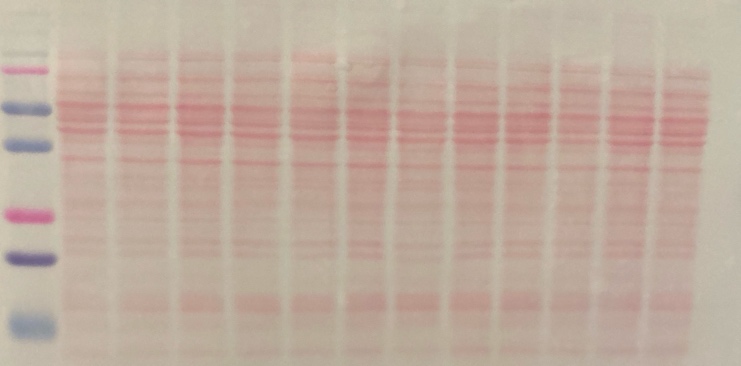
**

**FIGURE 2A, RBM3 detection in C2C12 at 24h, Experiment #1**

**
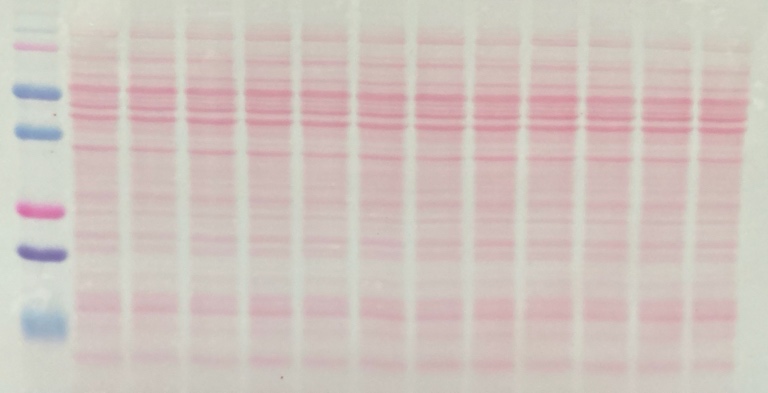
**

**FIGURE 2A, RBM3 detection in C2C12 at 24h, Experiment #2**

**
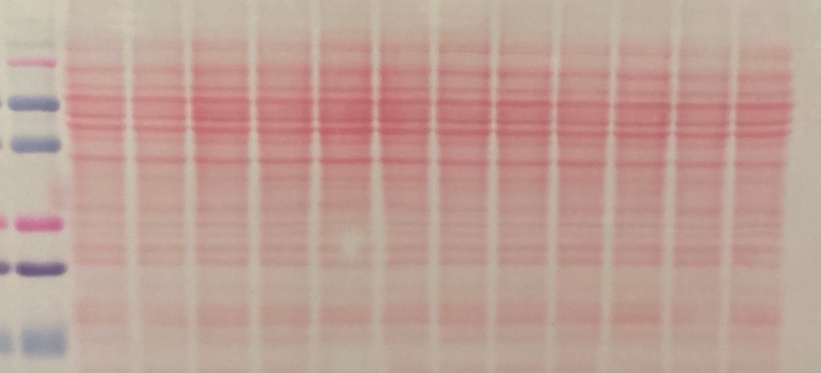
**

**FIGURE 2A, RBM3 detection in C2C12 at 6h and 24h, Experiment #3 Gel #1**


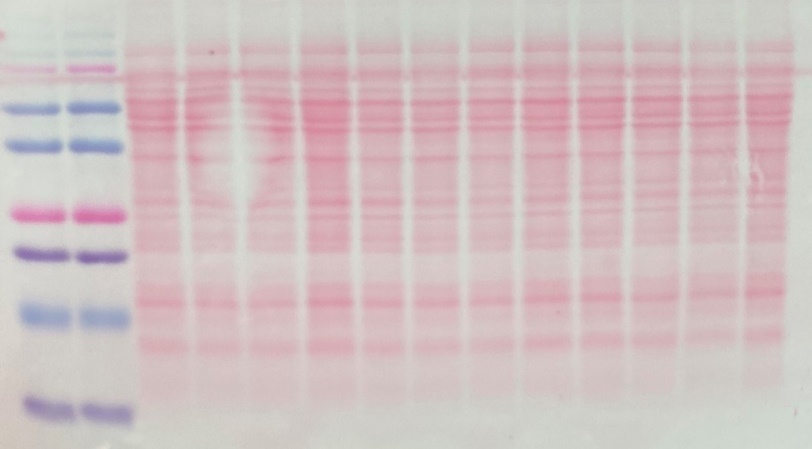


**FIGURE 2A, RBM3 detection in C2C12 at 6h and 24h, Experiment #3 Gel #1**


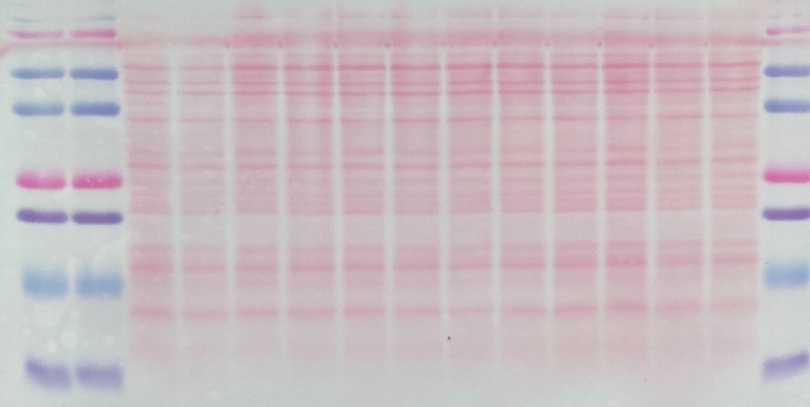


**FIGURE 3C, THBS-1 detection in C2C12 at 24h**

**
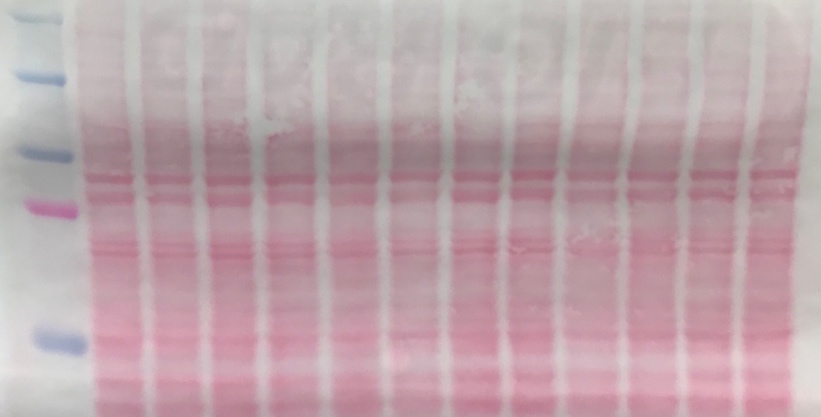
**

**FIGURE 3A, THBS-1 detection in C2C12 secreted fraction at 24h, Experiment #1**


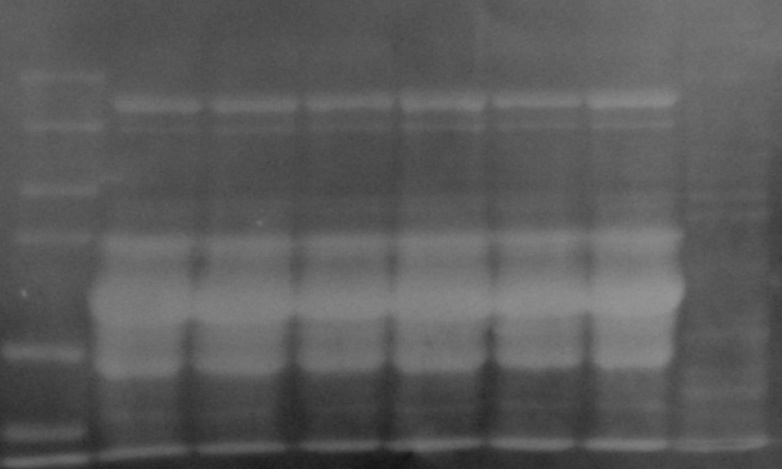


**FIGURE 3A, THBS-1 detection in C2C12 secreted fraction at 24h, Experiment #2**


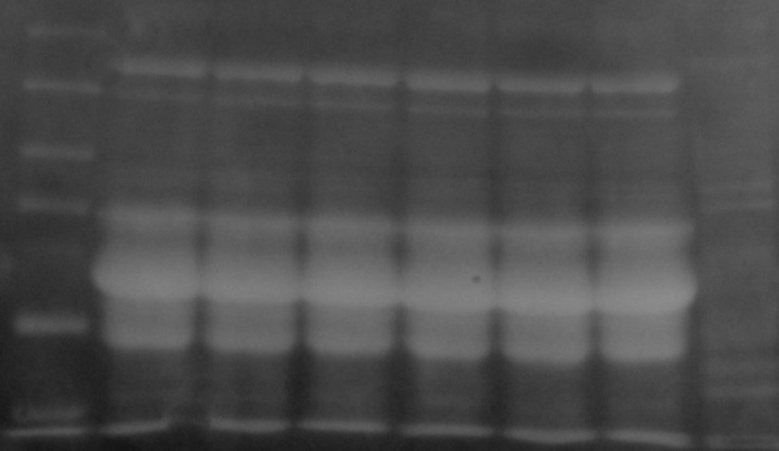


**FIGURE 3A, THBS-1 detection in C2C12 secreted fraction at 24h, Experiment #3**


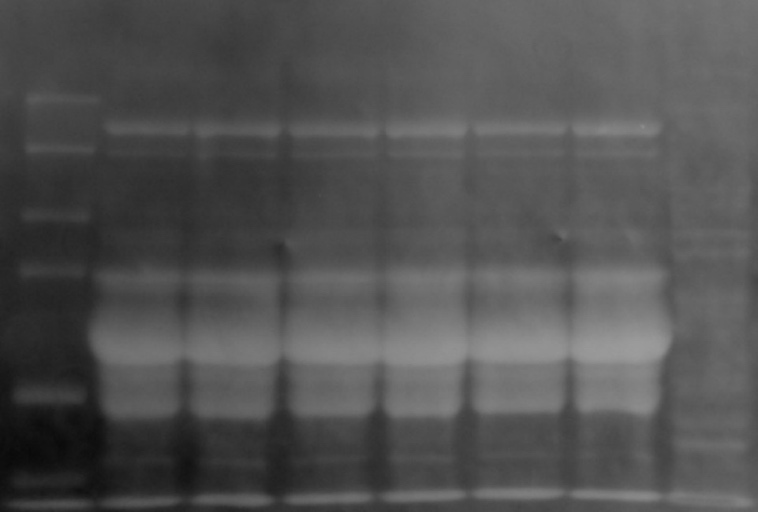


**FIGURE 4A, THBS-1 detection in C2C12 at 6h and 24h, Experiment #1, Gel #2**

**
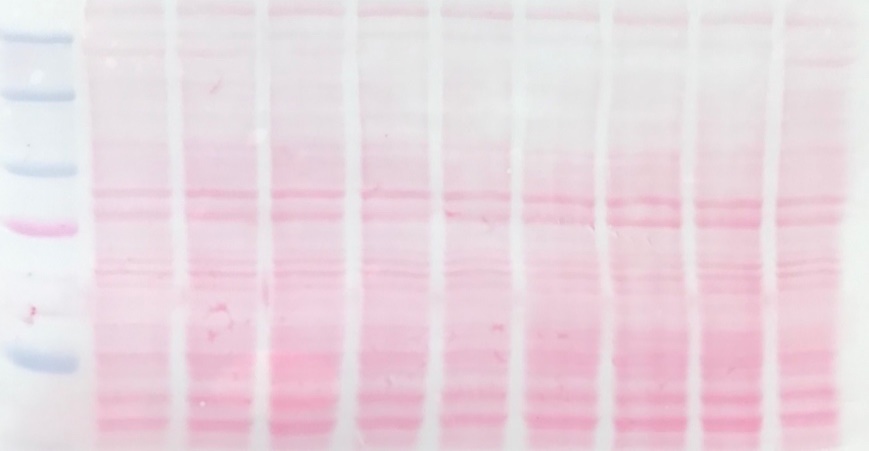
**

**FIGURE 4A, THBS-1 detection in C2C12 at 6h and 24h, Experiment #2, Gel #3**

**
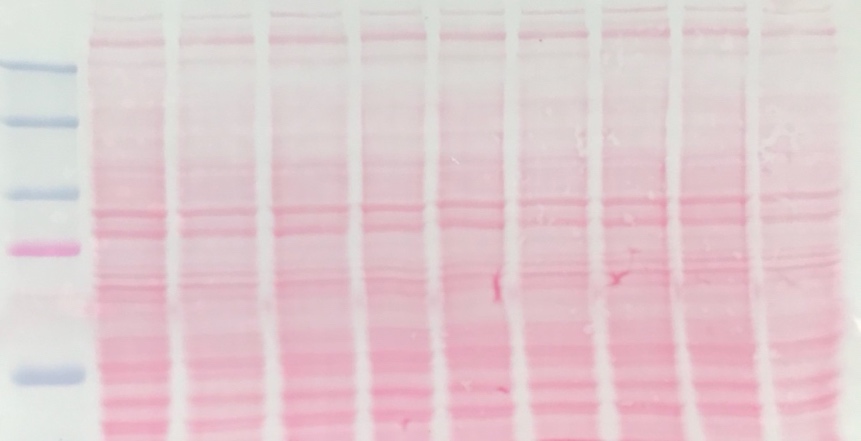
**

**FIGURE 4A, THBS-1 detection in C2C12 at 6h and 24h, Experiment #3, Gel #3**

**
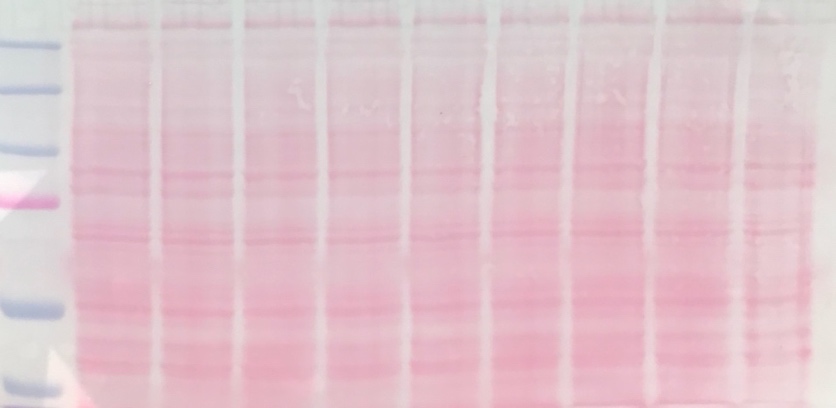
**

**FIGURE 4A, THBS-1 detection in C2C12 at 6h and 24h, Representative (Gel #1)**

**
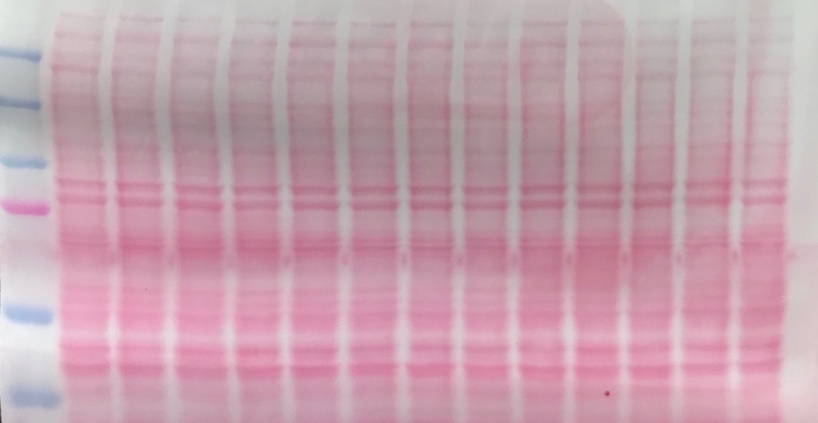
**

**FIGURE 4C, THBS-1 detection in C2C12 (Rewarming), Experiment #1, Gel #2**

**
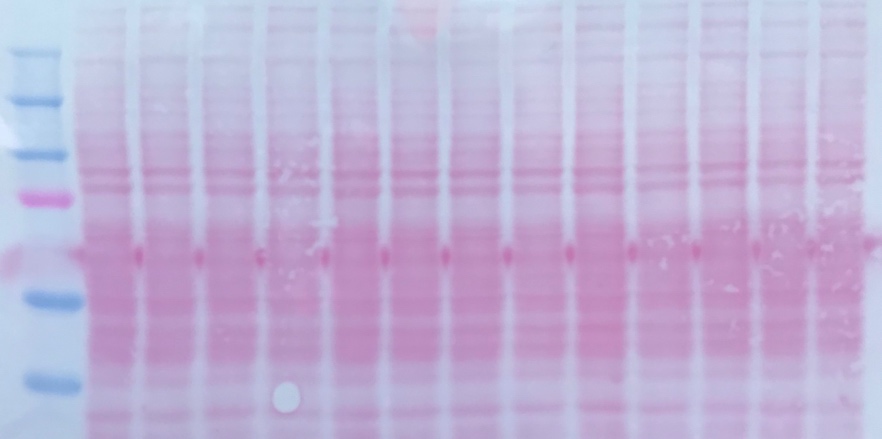
**

**FIGURE 4C, THBS-1 detection in C2C12 (Rewarming), Experiment #2, Gel #1**

**
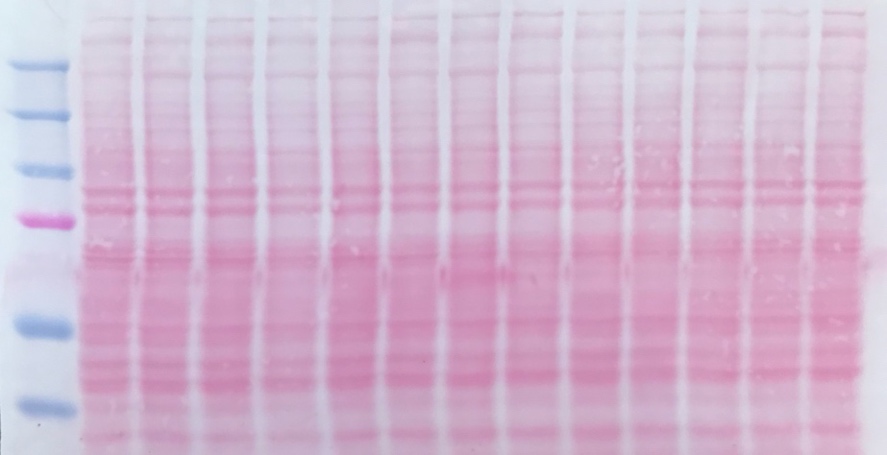
**

**FIGURE 7, RBM3 detection in SMECs, Experiment #1, 24h**

**
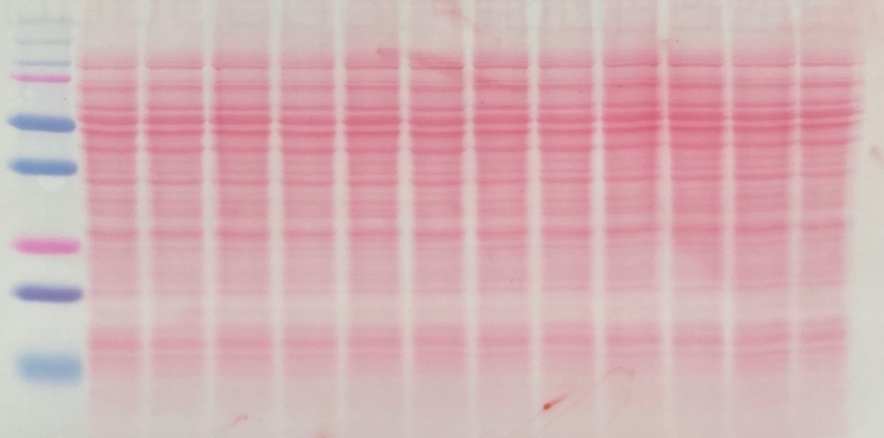
**

**FIGURE 7, RBM3 detection in SMECs, Experiment #2, 24h**

**
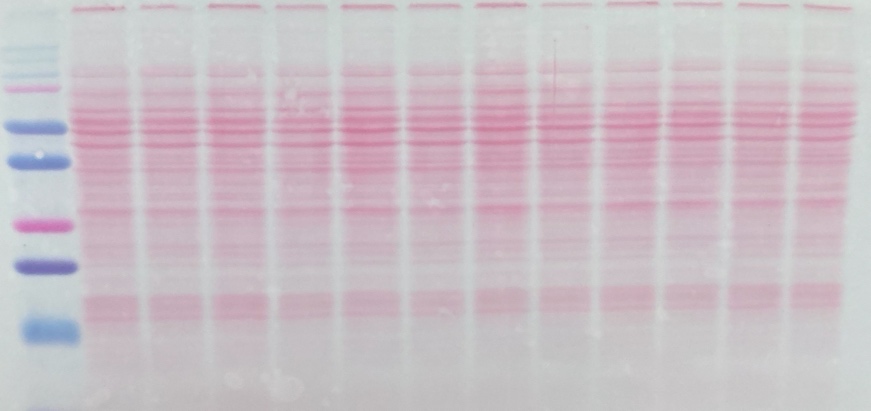
**

**FIGURE 7, RBM3 detection in SMECs, Experiment #3, 24h**


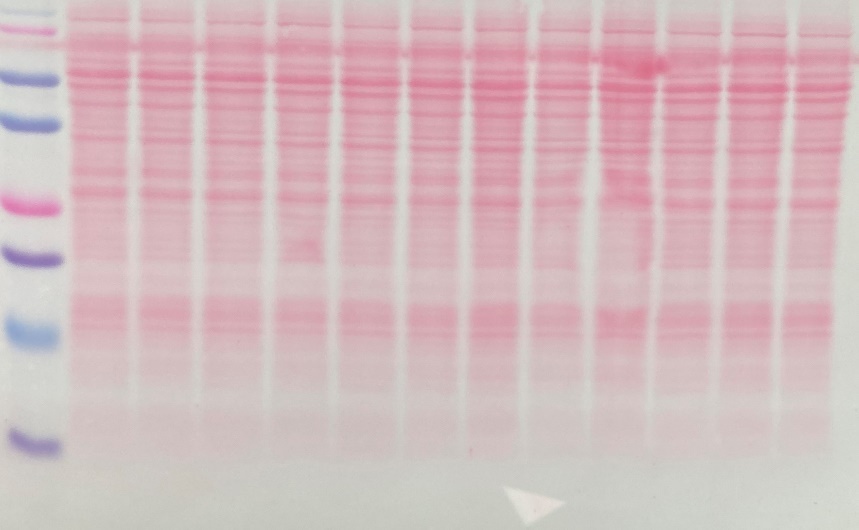


**FIGURE 9A, VEGFR2 detection in SMECs, Experiment #1**

**
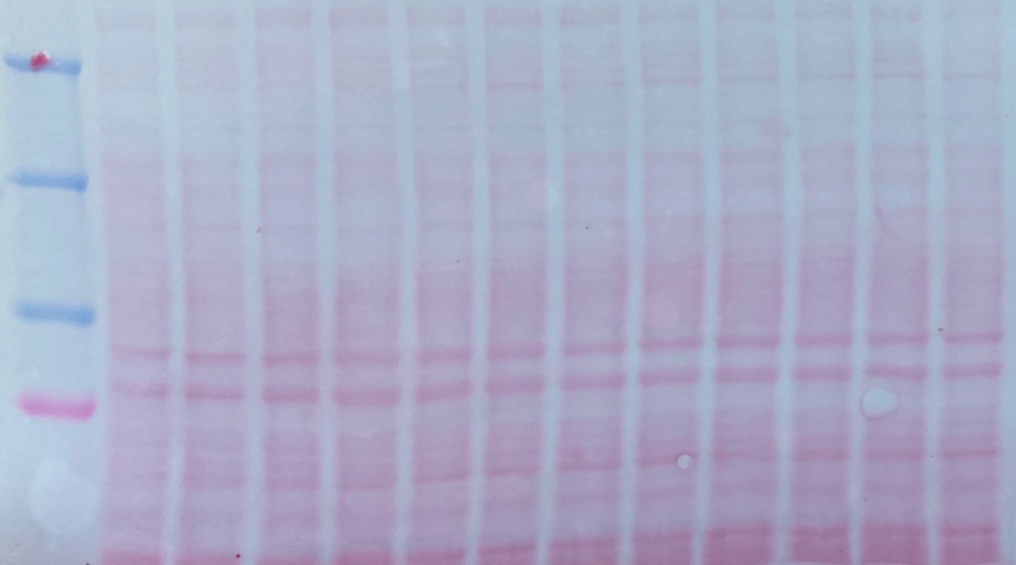
**

**FIGURE 9A, VEGFR2 detection in SMECs, Experiment #2**

**
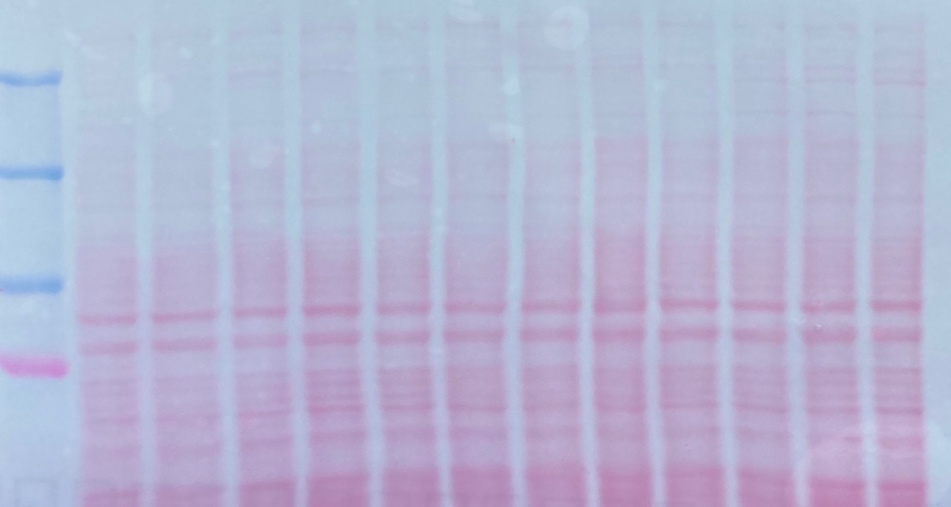
**

**FIGURE 9A, VEGFR2 detection in SMECs, Experiment #3**

**
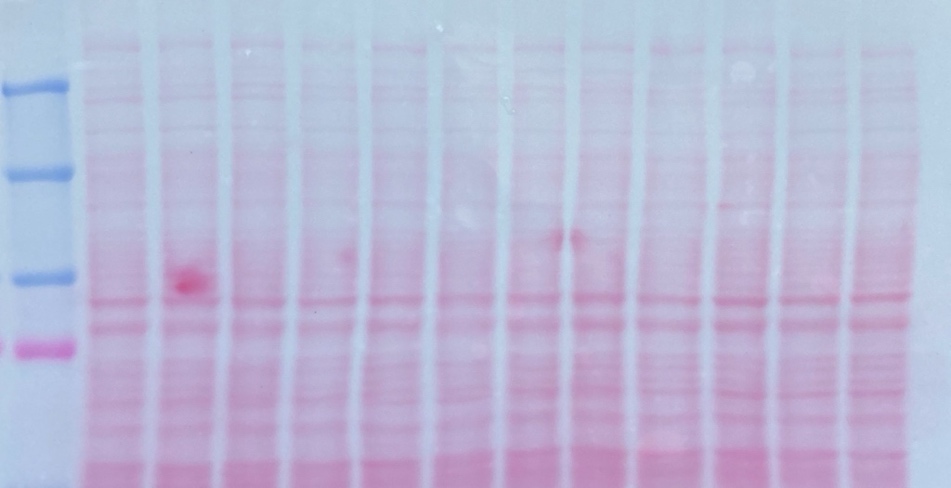
**

**FIGURE 9C, Y1175 detection in SMECs, Experiment #1**

**
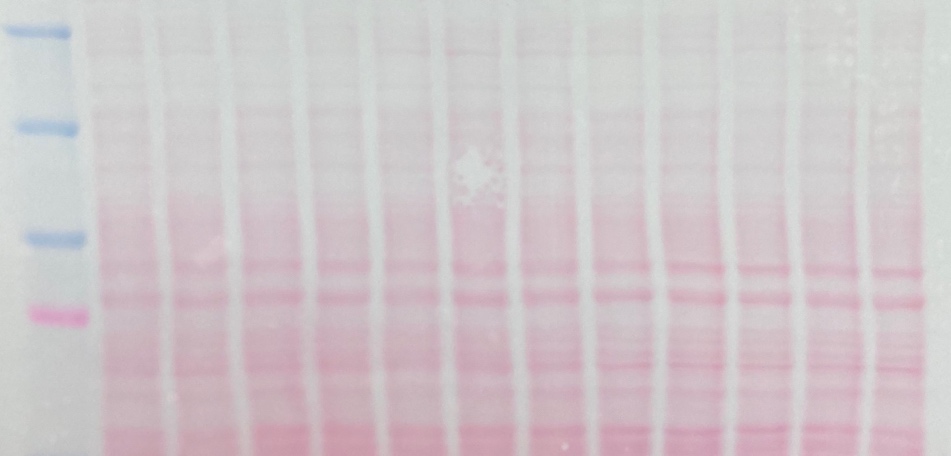
**

**FIGURE 9C, Y1175 detection in SMECs, Experiment #2**

**
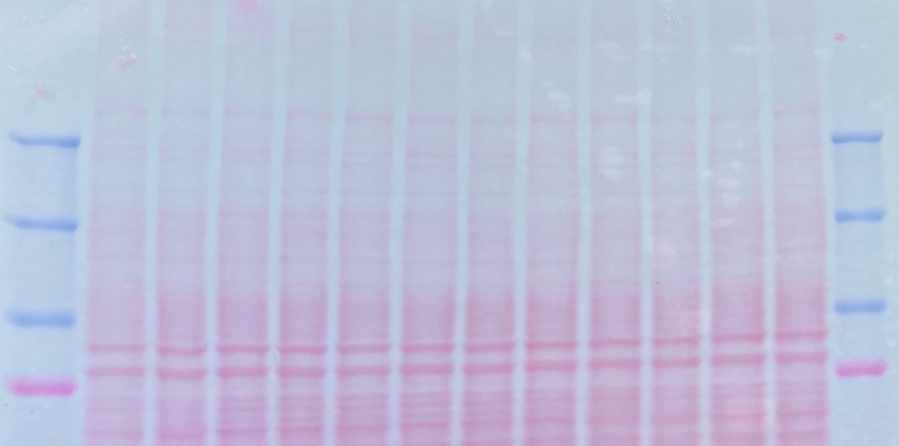
**

**FIGURE 9C, Y1175 detection in SMECs, Experiment #3**

**
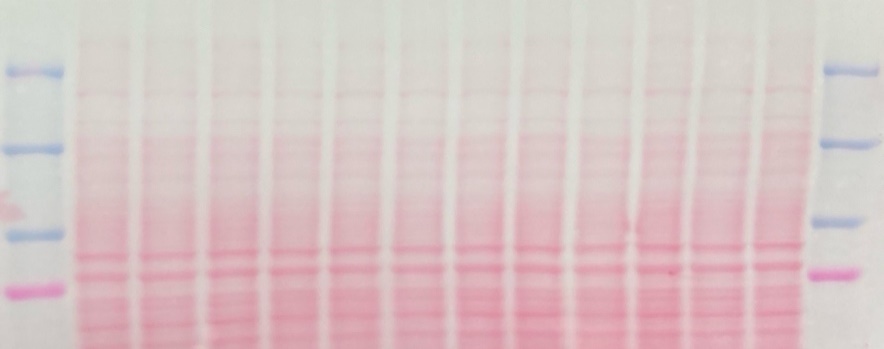
**

**FIGURE 11A, RBM3 detection in vastus lateralis fragments, Gel #1**

**
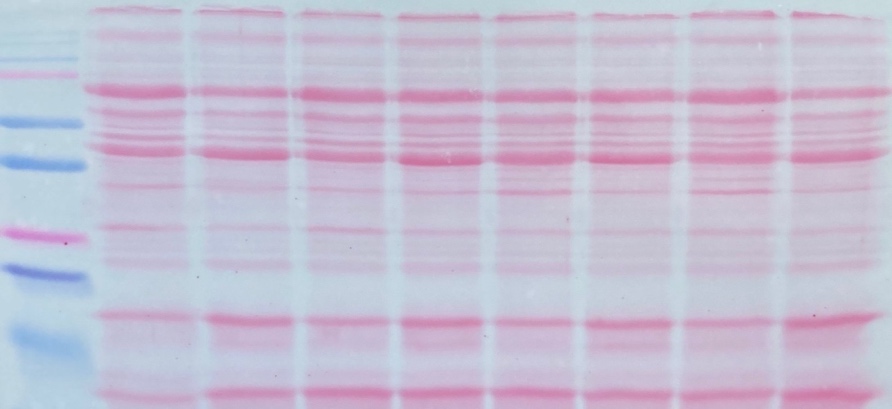
**

**FIGURE 11B, THBS-1 detection in vastus lateralis fragments, Gel #1**

**
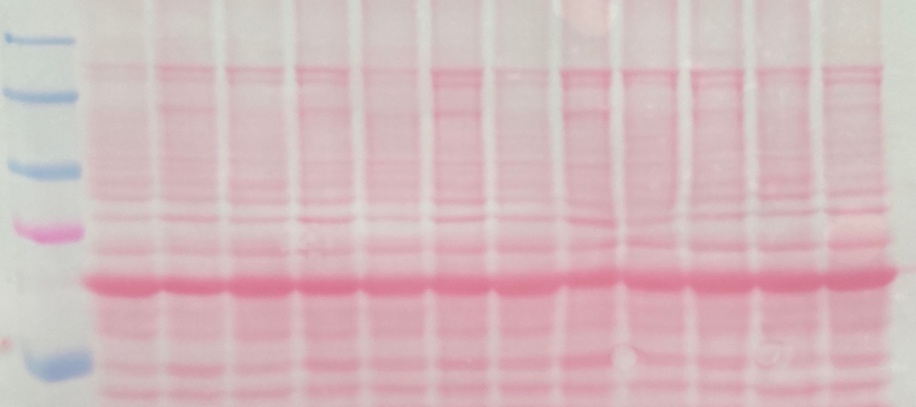
**

**4 Uncropped images from all western blots**

**Figure 2A:** C2C12 myotubes exposed to cold stress **(28)** or control **(37)** conditions for **6h** and **24h**. RBM3 expression determined in cell lysate. RBM3 protein immunoblotted with clone EPR6061. B-actin used as a loading control, clone C4. n=3 independent experiments **(EXP1,2,3)** with 6 samples **(1-6)** per condition.

**LOADING (SAME FOR EXP1,2)**

| **37-6h-1** | **28-6h-1** | **37-6h-2** | **28-6h-2** | **37-6h-3** | **28-6h-3** | **37-6h-4** | **28-6h-4** | **37-6h-5** | **28-6h-5** | **37-6h-6** | **28-6h-6** |
| --- | --- | --- | --- | --- | --- | --- | --- | --- | --- | --- | --- |

**EXP1-6h**

**RBM3 EPR6061**





**B-actin C4**


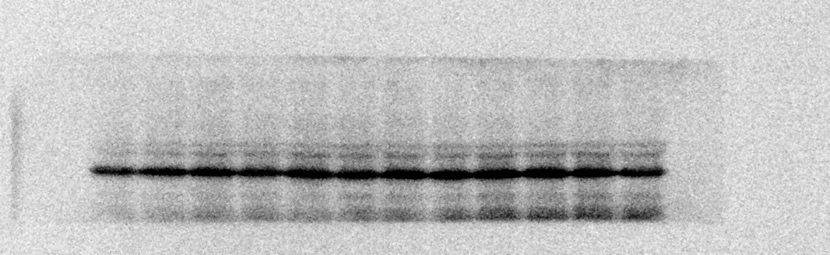


**EXP2-6h**

**RBM3 EPR6061**





**B-actin C4**


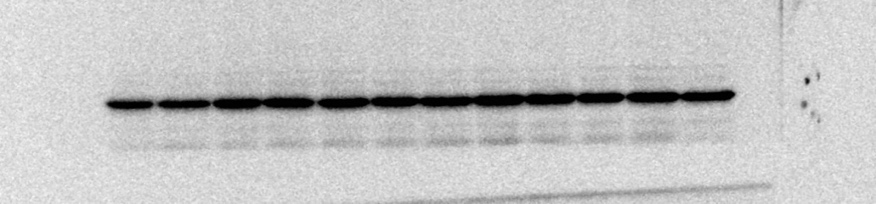


**LOADING (SAME FOR EXP1,2)**

| **37-24h-1** | **28-24h-1** | **37-24h-2** | **28-24h-2** | **37-24h-3** | **28-24h-3** | **37-24h-4** | **28-24h-4** | **37-24h-5** | **28-24h-5** | **37-24h-6** | **28-24h-6** |
| --- | --- | --- | --- | --- | --- | --- | --- | --- | --- | --- | --- |

**EXP1-24h**

**RBM3 EPR6061**


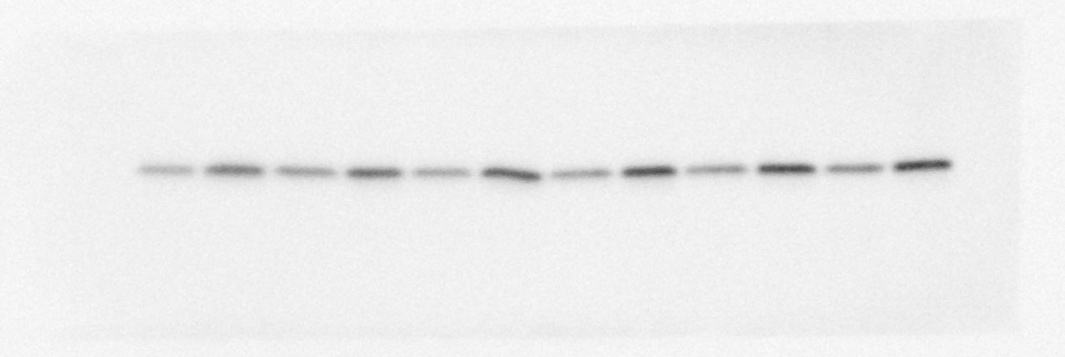


**B-actin C4**


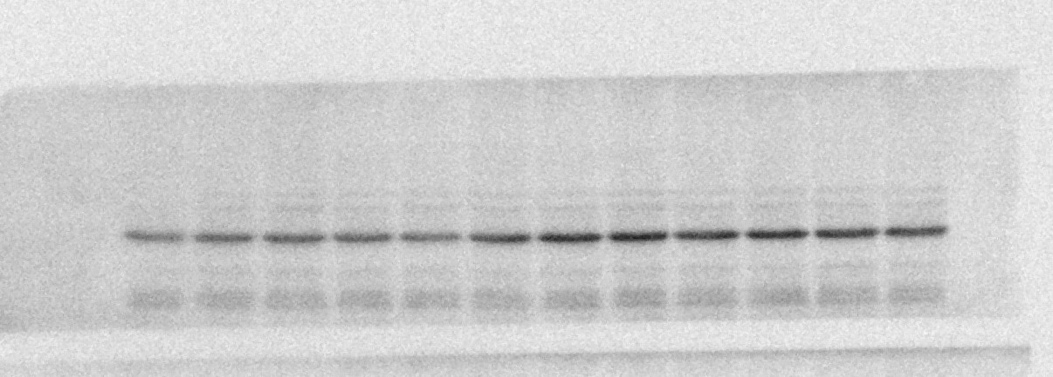


**EXP2-24h**

**RBM3 EPR6061**


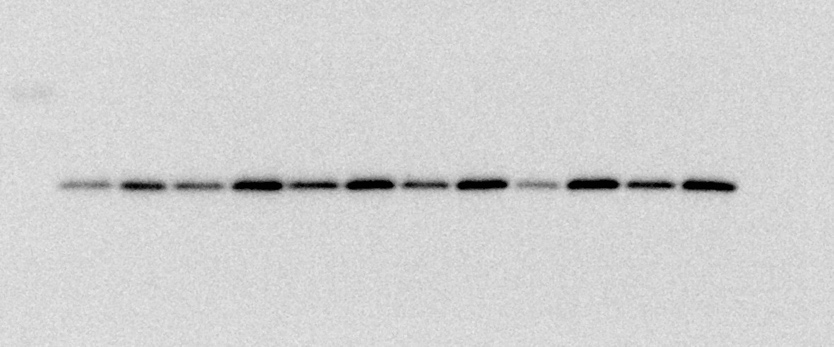


**B-actin C4**


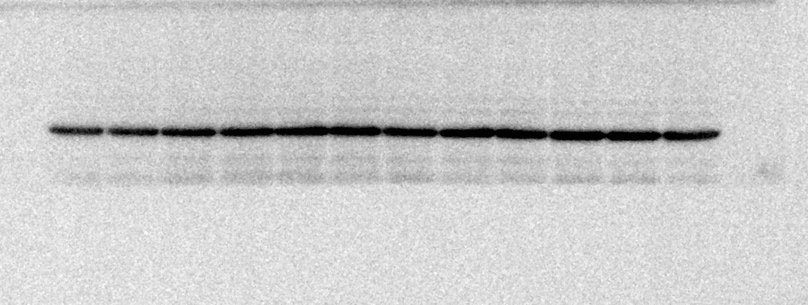


**EXP3**

**LOADING**

**GEL#1(TOP)**

| **37-6h-1** | **28-6h-1** | **37-24h-1** | **28-24h-1** | **37-6h-2** | **28-6h-2** | **37-24h-2** | **28-24h-2** | **37-6h-3** | **28-6h-3** | **37-24h-3** | **28-24h-3** |
| --- | --- | --- | --- | --- | --- | --- | --- | --- | --- | --- | --- |

**GEL#2(BOTTOM)**

| **37-6h-4** | **28-6h-4** | **37-24h-4** | **28-24h-4** | **37-6h-5** | **28-6h-5** | **37-24h-5** | **28-24h-5** | **37-6h-6** | **28-6h-6** | **37-24h-6** | **28-24h-6** |
| --- | --- | --- | --- | --- | --- | --- | --- | --- | --- | --- | --- |

**RBM3 EPR6061**


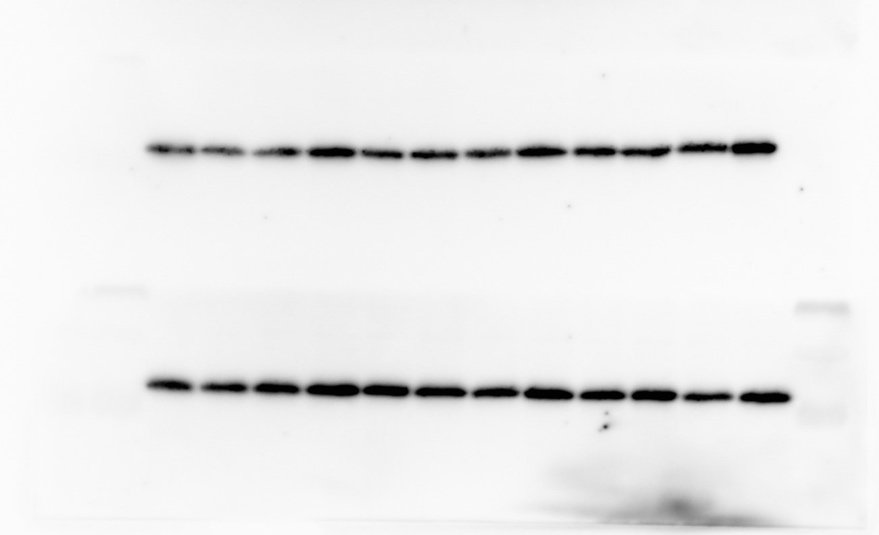


**B-actin C4**


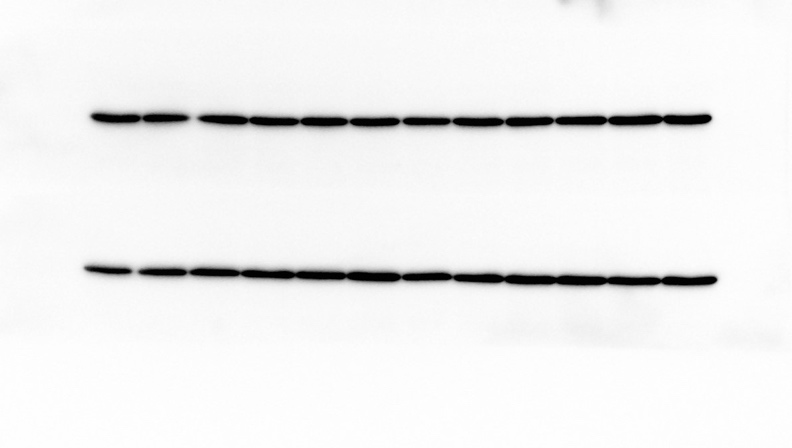


**Figure 3A:** C2C12 myotubes exposed to cold stress **(28)** or control **(37)** conditions for 24h. Cell media analyzed for secreted THBS1 protein expression. THBS1 immunoblotted with clone A6.1. Ponceau S. Stain used as a loading control. n=3 independent experiments **(EXP)** with n=3 samples **(1-3)** per condition.

**LOADING**

**GEL #1 (EXP1)**

| **37-24h-1** | **28-24h-1** | **37-24h-2** | **28-24h-2** | **37-24h-3** | **28-24h-3** | **C2C12**  **Intracellular** |
| --- | --- | --- | --- | --- | --- | --- |

**GEL #2 (EXP2)**

| **37-24h-1** | **28-24h-1** | **37-24h-2** | **28-24h-2** | **37-24h-3** | **28-24h-3** | **C2C12**  **cell lysate** |
| --- | --- | --- | --- | --- | --- | --- |

**GEL #3 (EXP3)**

| **37-24h-1** | **28-24h-1** | **37-24h-2** | **28-24h-2** | **37-24h-3** | **28-24h-3** | **C2C12**  **cell lysate** |
| --- | --- | --- | --- | --- | --- | --- |

**THBS1 A6.1 GEL #1 (TOP) and GEL #2 (BOTTOM)**





**THBS1 A6.1 GEL #3**


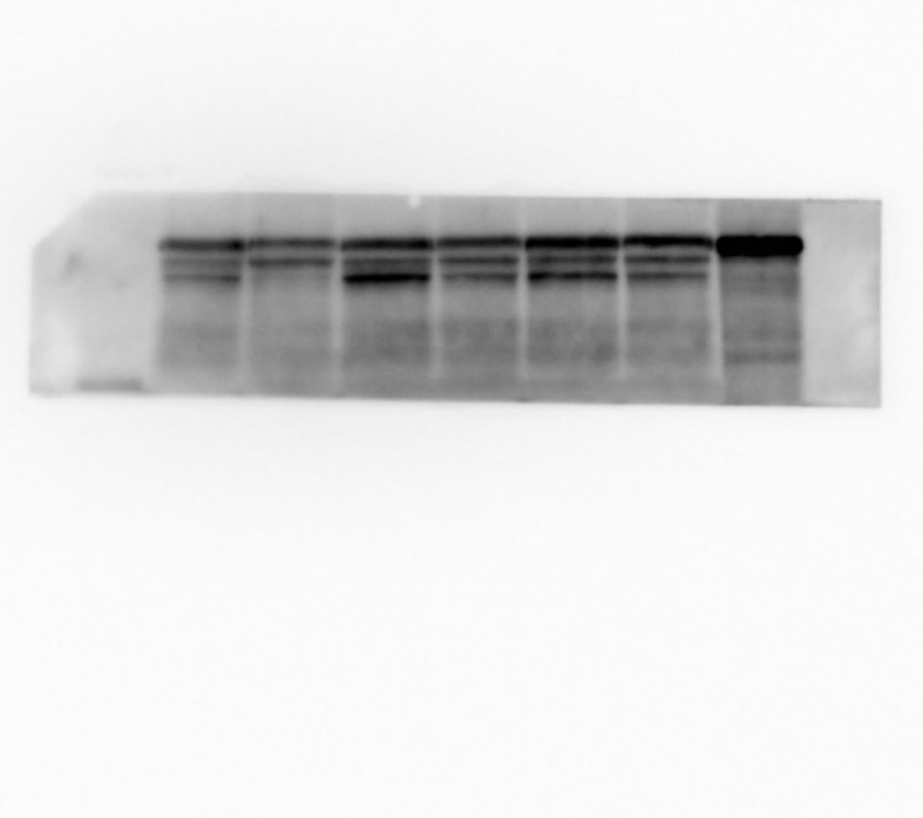


**Ponceau S GEL #1**





**Ponceau S GEL #2**





**Ponceau S GEL #3**





**FIGURE 3A CONTINUED EXP4**

C2C12 myotubes exposed to cold stress **(28)** or control **(37)** conditions for **24h**. THBS1 protein expression in cell lysate immunoblotted with A6.1 clone, Ponceau S stain used as loading control. n=1 independent experiments with 6 samples **(1-6)** per condition.

**LOADING:**

**GEL#1 (TOP)**

| **37-24h-1** | **28-24h-1** | **37-24h-2** | **28-24h-2** | **37-24h-3** | **28-24h-3** | **C2C12**  **cell lysate** |
| --- | --- | --- | --- | --- | --- | --- |

**GEL #2 (BOTTOM)**

| **37-24h-4** | **28-24h-4** | **37-24h-5** | **28-24h-5** | **37-24h-6** | **28-24h-6** | **C2C12**  **cell lysate** |
| --- | --- | --- | --- | --- | --- | --- |

**THBS1 A6.1, Gel #1 (TOP), Gel #2 (BOTTOM)**





**Ponceau S. Stain GEL #1**





**Ponceau S. Stain GEL #2**





**Figure 3B:** Qualitative assessment of THBS1 protein expression in C2C12 cell lysate, C2C12 cell media, Differentiation media, Horse Serum and DMEM. THBS1 immunoblotted with clone A6.1. n=1 independent experiment.

**LOADING**

| **C2C12 cell lysate** | **C2C12 cell media** | **C2C12 cell lysate** | **Differentiation media** | **Horse Serum** | **DMEM** |
| --- | --- | --- | --- | --- | --- |

**TOP GEL USED IN MANUSCRIPT, A6.1 INVITROGEN THBS1**





**Figure 3C:** C2C12 myotubes exposed to cold stress **(28)** or control **(37)** conditions for 24h. Cellular expression of THBS1 immunoblotted with clone A6.1. B-actin used as a loading control. n=1 independent experiments with 6 samples **(1-6)** per condition.

**LOADING:**

| **37-24h-1** | **28-24h-1** | **37-24h-2** | **28-24h-2** | **37-24h-3** | **28-24h-3** | **37-24h-4** | **28-24h-4** | **37-24h-5** | **28-24h-5** | **37-24h-6** | **28-24h-6** |
| --- | --- | --- | --- | --- | --- | --- | --- | --- | --- | --- | --- |

**THBS1 A6.1**





**B-actin C4**


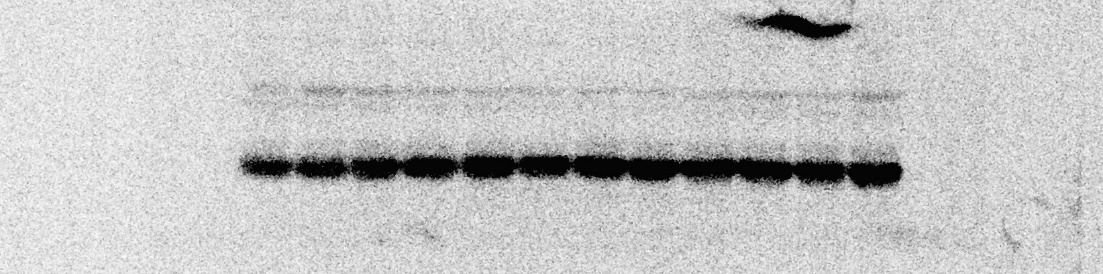


**Figure 4A:** C2C12 myotubes exposed to cold stress **(28)** or control **(37)** conditions for 6h and 24h. Cellular THBS1 protein expression immunoblotted by clone A6.1. B-actin used as a loading control, clone C4. n=3 independent experiments **(EXP)** with 6 samples **(1-6)** per condition **(3 Gels per experiment)**. n=1 representative blot with 6 samples **(1-6)** per condition **(2 gels)**.

**LOADING (SAME FOR EXP1, EXP2, EXP3).**

**GEL #1 (TOP)**

| **37-6h-1** | **28-6h-1** | **37-24h-1** | **28-24h-1** | **37-6h-2** | **28-6h-2** | **37-24h-2** | **28-24h-2** | **Pool** |
| --- | --- | --- | --- | --- | --- | --- | --- | --- |

**GEL #2 (MIDDLE)**

| **37-6h-3** | **28-6h-3** | **37-24h-3** | **28-24h-3** | **37-6h-4** | **28-6h-4** | **37-24h-4** | **28-24h-4** | **Pool** |
| --- | --- | --- | --- | --- | --- | --- | --- | --- |

**GEL #3 (BOTTOM)**

| **37-6h-5** | **28-6h-5** | **37-24h-5** | **28-24h-5** | **37-6h-6** | **28-6h-6** | **37-24h-6** | **28-24h-6** | **Pool** |
| --- | --- | --- | --- | --- | --- | --- | --- | --- |

**EXP1**

**THBS1 A6.1**





**B-actin C4**





**EXP 2**

**THBS1 A6.1**





**B-actin C4**





**EXP3**

**THBS1 A6.1 GEL #1 (BOTTOM)**





**B-actin C4 GEL #1**


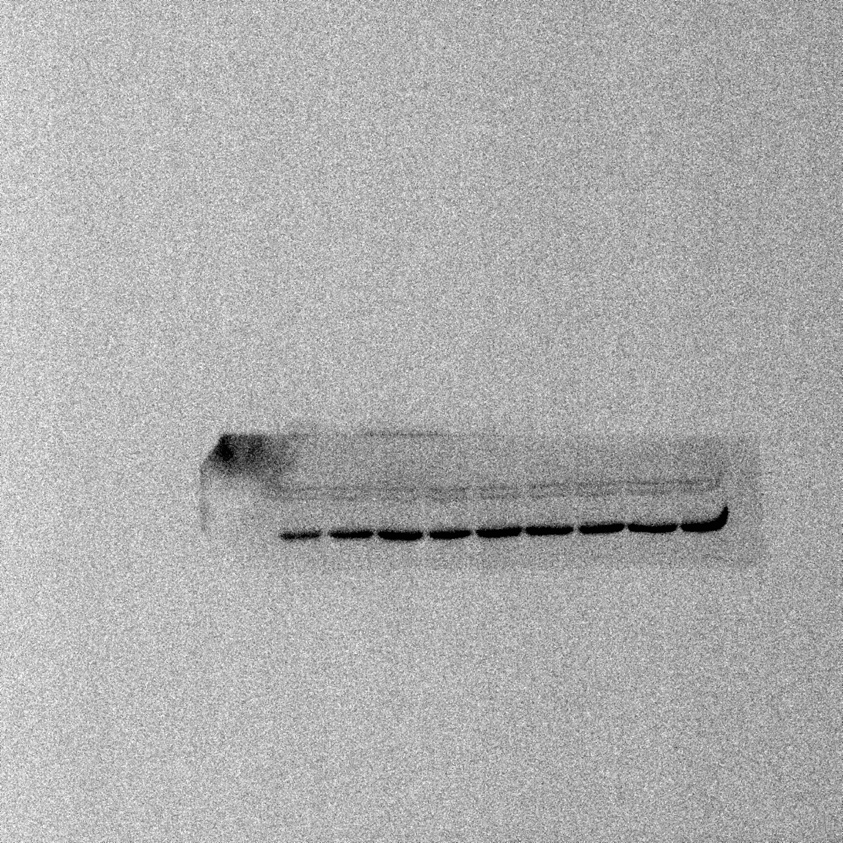


**THBS1 A6.1 GEL #2 (MIDDLE) GEL #3 (BOTTOM)**





**B-actin C4 GEL #2 (MIDDLE) GEL #3 (BOTTOM)**


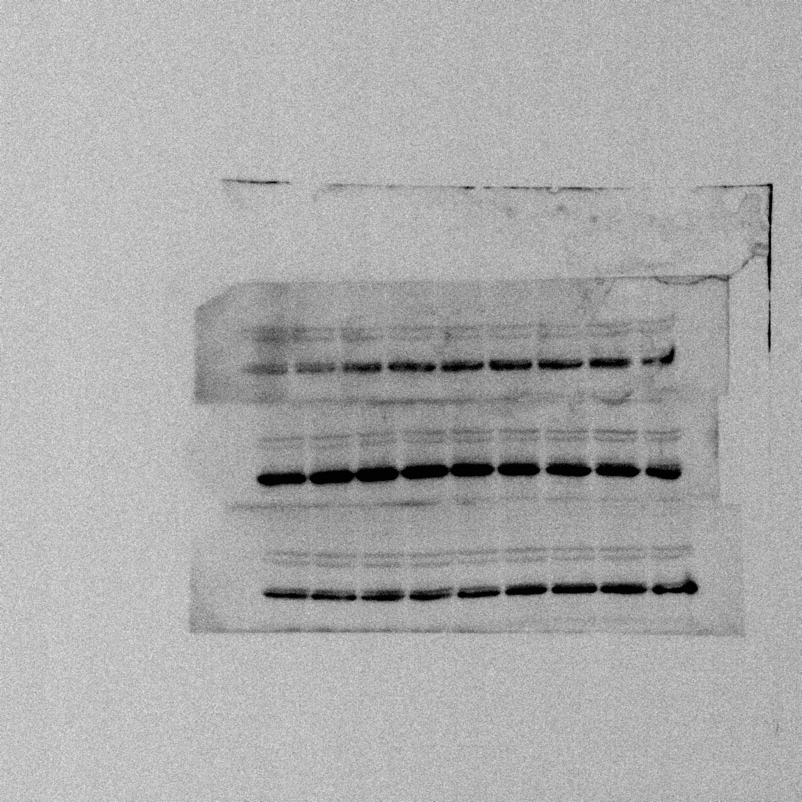


**REPRESENATIVE BLOT (NOT EXP 1,2,3,)**

**LOADING**

**GEL #1 (TOP)**

| **37-6h-1** | **28-6h-1** | **37-24h-1** | **28-24h-1** | **37-6h-2** | **28-6h-2** | **37-24h-2** | **28-24h-2** | **37-6h-3** | **28-6h-3** | **37-24h-3** | **28-24h-3** | **Pool** |
| --- | --- | --- | --- | --- | --- | --- | --- | --- | --- | --- | --- | --- |

**GEL #2 (BOTTOM)**

| **37-6h-4** | **28-6h-4** | **37-24h-4** | **28-24h-4** | **37-6h-5** | **28-6h-5** | **37-24h-5** | **28-24h-5** | **37-6h-6** | **28-6h-6** | **37-24h-6** | **28-24h-6** | **Pool** |
| --- | --- | --- | --- | --- | --- | --- | --- | --- | --- | --- | --- | --- |

**THBS1 A6.1**





**B-actin C4**





**Figure 4C:** C2C12 myotubes exposed to cold stress **(28)**, control **(37)**, re-warming **(RW)** and re-cooling **(RC)** conditions. Cellular THBS1 protein expression by immunoblotting with clone A6.1. B-actin used as a loading control, Clone C4. n=2 independent experiments **(EXP)** with 6 samples **(1-6)** per condition.

**LOADING (SAME FOR EXP1,2)**

**GEL #1 (TOP)**

| **37-12h-1** | **28-12h-1** | **RW-1** | **RC-1** | **37-12h-2** | **28-12h-2** | **RW-2** | **RC-2** | **37-12h-3** | **28-12h-3** | **RW-3** | **RC-3** | **Pool** |
| --- | --- | --- | --- | --- | --- | --- | --- | --- | --- | --- | --- | --- |

**GEL #2 (BOTTOM)**

| **37-12h-4** | **28-12h-4** | **RW-4** | **RC-4** | **37-12h-5** | **28-12h-5** | **RW-5** | **RC-5** | **37-12h-6** | **28-12h-6** | **RW-6** | **RC-6** | **Pool** |
| --- | --- | --- | --- | --- | --- | --- | --- | --- | --- | --- | --- | --- |

**EXP1**

**THBS1 A6.1**





**B-actin C4**





**EXP2**

**THBS1 A6.1**





**B-actin C4**





**Figure 7A:** RBM3 cellular protein expression in SMECs exposed to cold stress **(28)** or control **(37)** conditions for **24h**. RBM3 immunoblotted with clone EPR6061. B-actin used as a loading control, clone C4. n=3 independent experiments **(EXP)** with 6 samples **(1-6)** per condition.

**LOADING (SAME FOR EXP1,2,3)**

| **37-24h-1** | **28-24h-1** | **37-24h-2** | **28-24h-2** | **37-24h-3** | **28-24h-3** | **37-24h-4** | **28-24h-4** | **37-24h-5** | **28-24h-5** | **37-24h-6** | **28-24h-6** |
| --- | --- | --- | --- | --- | --- | --- | --- | --- | --- | --- | --- |

**EXP1**

**RBM3 EPR6061**





**B-actin C4**





**EXP2**

**RBM3 EPR6061**


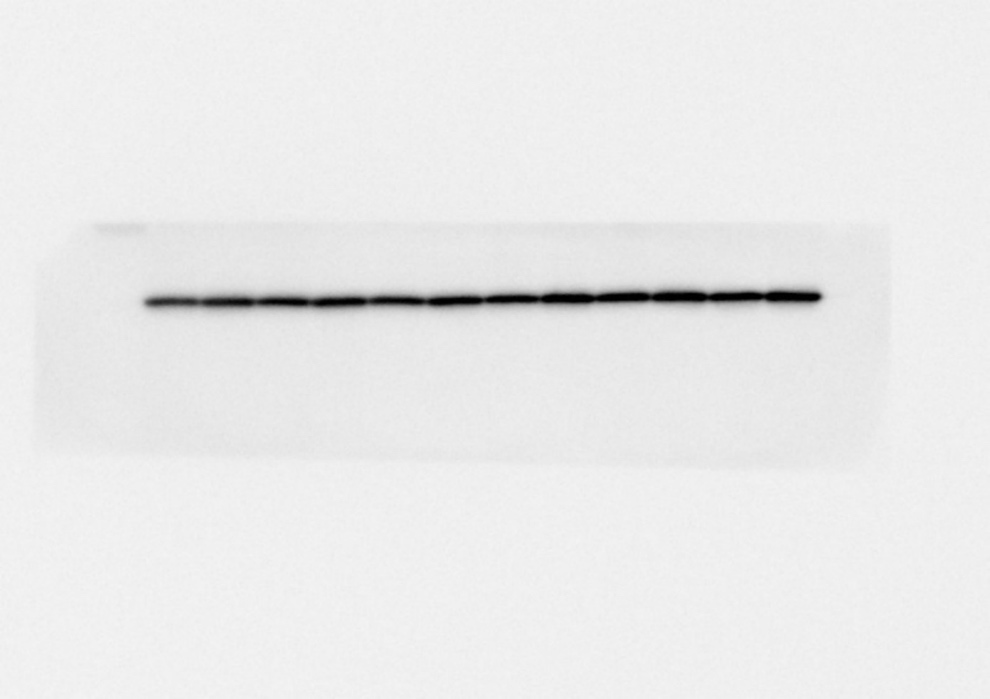


**B-actin C4**





**EXP3**

**RBM3 EPR6061**


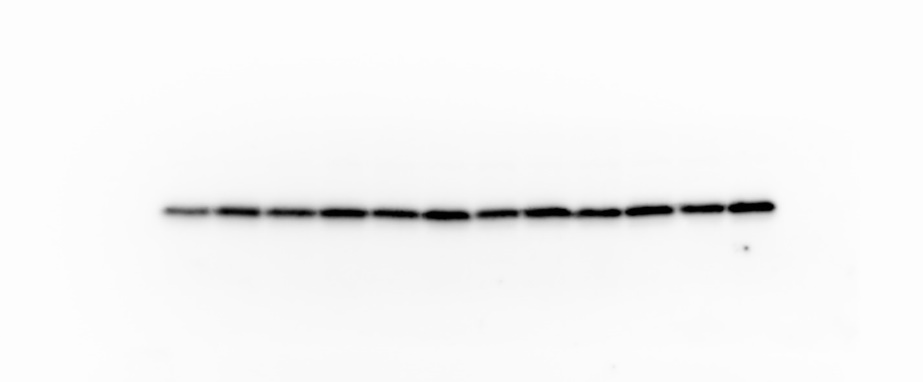


**B-actin C4**


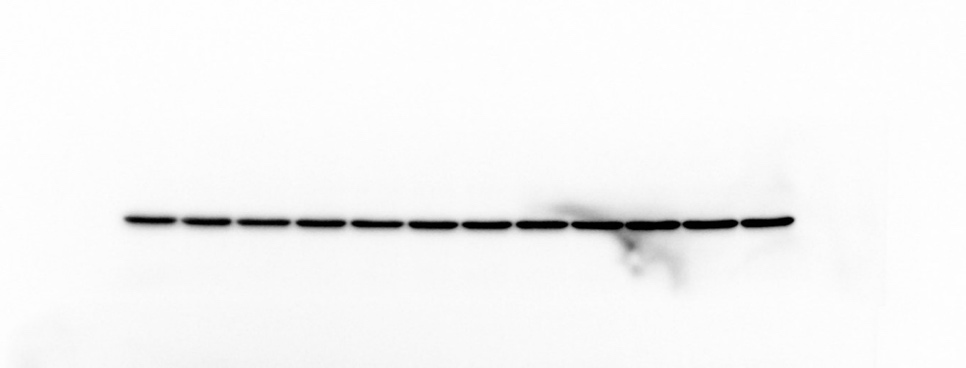


**Figure 9A:** VEGFR2 cellular expression in SMECs exposed to cold stress **(28)** or control **(37)** conditions for **24h** and stimulated with or without recombinant VEGF-A **(mVEGF-A165)**. VEGFR2 immunoblotted with clone D5B1. A/B-tubulin used as a loading control, polyclonal. n=3 independent experiments **(EXP)** with 3 samples **(1-3)** per condition.

**LOADING (SAME FOR EXP 1,2,3)**

| **37-24h-1** | **28-24h-1** | **37-24h-1** | **28-24h-1** | **37-24h-2** | **28-24h-2** | **37-24h-2** | **28-24h-2** | **37-24h-3** | **28-24h-3** | **37-24h-3** | **28-24h-3** |
| --- | --- | --- | --- | --- | --- | --- | --- | --- | --- | --- | --- |
| **-VEGFA** | **-**  **VEGFA** | **+**  **VEGFA** | **+**  **VEGFA** | **-**  **VEGFA** | **-**  **VEGFA** | **+**  **VEGFA** | **+**  **VEGFA** | **-**  **VEGFA** | **-**  **VEGFA** | **+**  **VEGFA** | **+**  **VEGFA** |

**EXP1**

**VEGFR2 D5B1**

**Tubulin Polyclonal**

**EXP2**

**VEGFR2 D5B1**

**Tubulin Polyclonal**

**EXP3**

**VEGFR2 D5B1**

**Tubulin Polyclonal**

**Figure 9C:** p-VEGFR2-Y1175 cellular expression in SMECs exposed to cold stress **(28)** or control **(37)** conditions for **24h** and stimulated **with or without recombinant VEGF-A** (mVEGF-A165). p-VEGFR2-Y1175 immunoblotted with clone D5B11. A/B-tubulin used as a loading control, polyclonal. n=3 independent experiments **(EXP)** with 3 samples **(1-3)** per condition.

**SMEC intracellular p-VEGFR2-Y1175 EXP1**

**LOADING (SAME FOR EXP1,2,3)**

| **37-24h-1** | **28-24h-1** | **37-24h-1** | **28-24h-1** | **37-24h-2** | **28-24h-2** | **37-24h-2** | **28-24h-2** | **37-24h-3** | **28-24h-3** | **37-24h-3** | **28-24h-3** |
| --- | --- | --- | --- | --- | --- | --- | --- | --- | --- | --- | --- |
| **-VEGFA** | **-**  **VEGFA** | **+**  **VEGFA** | **+**  **VEGFA** | **-**  **VEGFA** | **-**  **VEGFA** | **+**  **VEGFA** | **+**  **VEGFA** | **-**  **VEGFA** | **-**  **VEGFA** | **+**  **VEGFA** | **+**  **VEGFA** |

**EXP1**

**p-VEGFR2-Y1175 D5B11**

**Tubulin Polyclonal**

**EXP2**

**p-VEGFR2-Y1175 D5B11**

**Tubulin Polyclonal**

**EXP3**

**p-VEGFR2-Y1175 D5B11**

**Tubulin Polyclonal**

**Figure 11A:** RBM3 cellular protein expression in ex-vivo muscle incubation assay (EMI) vastus lateralis muscle fragments exposed to cold stress **(28)** or control **(37)** conditions for 24h. RBM3 immunoblotted with clone EPR6061. B-actin used as a loading control, clone C4. n=1 independent experiment with 7 samples **(1-7)** per condition.

**LOADING**

**GEL #1 (TOP)**

| **37-24h-1** | **28-24h-1** | **37-24h-2** | **28-24h-2** | **37-24h-3** | **28-24h-3** | **37-24h-4** | **28-24h-4** |
| --- | --- | --- | --- | --- | --- | --- | --- |

**GEL #2 (BOTTOM)**

| **37-24h-5** | **28-24h-5** | **37-24h-6** | **28-24h-6** | **37-24h-7** | **28-24h-7** |
| --- | --- | --- | --- | --- | --- |

**RBM3 EPR6061**

**B-actin C4**

**Figure 11B:** THBS1 cellular protein expression in EMI samples exposed to cold stress **(28)** or control **(37)** conditions for 24h. THBS1 immunoblotted with clone D7EF5. B-actin used as a loading control, clone C4. n=1 independent experiment with 6 samples **(1-6)** per condition.

**LOADING**

| **37-24h-1** | **28-24h-1** | **37-24h-2** | **28-24h-2** | **37-24h-3** | **28-24h-3** | **37-24h-4** | **28-24h-4** | **37-24h-5** | **28-24h-5** | **37-24h-6** | **28-24h-6** |
| --- | --- | --- | --- | --- | --- | --- | --- | --- | --- | --- | --- |

**THBS1 D7EF5**

**B-actin C4**
